# Robust nucleation control via crisscross polymerization of highly coordinated DNA slats

**DOI:** 10.1038/s41467-021-21755-7

**Published:** 2021-03-19

**Authors:** Dionis Minev, Christopher M. Wintersinger, Anastasia Ershova, William M. Shih

**Affiliations:** 1 John A. Paulson School of Engineering and Applied Sciences, Harvard University, Cambridge, MA USA; 2 Wyss Institute for Biologically Inspired Engineering at Harvard University, Boston, MA USA; 3 Department of Cancer Biology, Dana-Farber Cancer Institute, Boston, MA USA; 4 Department of Biological Chemistry and Molecular Pharmacology, Harvard Medical School, Boston, MA USA

**Keywords:** Nanostructures, DNA nanostructures

## Abstract

Natural biomolecular assemblies such as actin filaments or microtubules can exhibit all-or-nothing polymerization in a kinetically controlled fashion. The kinetic barrier to spontaneous nucleation arises in part from positive cooperativity deriving from joint-neighbor capture, where stable capture of incoming monomers requires straddling multiple subunits on a filament end. For programmable DNA self-assembly, it is likewise desirable to suppress spontaneous nucleation to enable powerful capabilities such as all-or-nothing assembly of nanostructures larger than a single DNA origami, ultrasensitive detection, and more robust algorithmic assembly. However, existing DNA assemblies use monomers with low coordination numbers that present an effective kinetic barrier only for slow, near-reversible growth conditions. Here we introduce crisscross polymerization of elongated slat monomers that engage beyond nearest neighbors which sustains the kinetic barrier under conditions that promote fast, irreversible growth. By implementing crisscross slats as single-stranded DNA, we attain strictly seed-initiated nucleation of crisscross ribbons with distinct widths and twists.

## Abstract (web summary)

For programmable DNA self-assembly, it is desirable to suppress spontaneous nucleation to enable all-or-nothing assembly of nanostructures far larger than a single DNA origami. Here the authors introduce crisscross polymerization of elongated slat monomers that engage beyond nearest neighbors, providing strictly seed-initiated nucleation of crisscross ribbons with distinct widths and twists.

## Introduction

DNA tiles or bricks have been shown to self-assemble non-periodic nanostructures much larger than what has been demonstrated thus far with any single DNA-origami scaffold while maintaining an excess of components during the assembly reaction^1–4^. This is enabled by folding in a cooperative regime, where nucleation is relatively slow and growth is comparatively fast. Rate-limiting spontaneous nucleation can be relied upon to initiate growth, however, control over the copy number of structures then will be limited. For periodic assembly, rate-limiting nucleation will lead to a wide distribution of sizes, which is disadvantageous when a relatively monodisperse product is desired. Introduction of seeds could provide a burst of controlled nucleation, nevertheless seed-independent nucleation will coincide under conditions that favor rapid growth (i.e., high concentrations of monomers and well below the reversible temperature), such that a subpopulation of smaller or incomplete assemblies will arise. In the case of algorithmic assembly, where each seed kinetically triggers a particular pattern of tile accretion, contamination from unseeded growths can be especially problematic^5–12^.

Enforcing seed-dependence in self-assembly requires balancing two opposing design criteria. A large kinetic barrier to prevent spontaneous nucleation (also called “spurious nucleation”) of monomers must be in place, while added seeds must readily bypass that barrier to yield rapid growth. Barriers can be constructed by kinetic trapping of monomers into inactive states (see Supplementary Discussion 1 for more discussion), and/or by making stable capture of incoming monomers contingent on the cooperative binding of two or more previously acquired neighbors (i.e., half the monomer coordination number)^1–38^. In the latter case—which we refer to here as joint-neighbor capture of monomers—a large coordination number would allow two prized characteristics of self-assembly that otherwise would be mutually exclusive for such systems: near-complete suppression of spontaneous nucleation^39,40^ along with rapid, irreversible growth from introduced seeds. Natural examples of assemblies utilizing joint-neighbor capture of incoming monomers include actin filaments and microtubules^24,25^, while synthetic examples include DNA tiling^1–12^ and living crystallization-driven self-assembly^30–32^. Most biological and synthetic assemblies are composed of monomers that bond only with nearest neighbors, which limits the monomer coordination number. Consequently, assembly in many of these cases proceeds with seed-dependence only for slow, near-reversible growth conditions.

We introduce crisscross slats as an architecture where slat monomers engage beyond just nearest neighbors to achieve any potential coordination number. We establish a theoretical comparison showing that crisscross assembly sustains kinetic barriers under fast, irreversible reaction conditions far better than existing DNA tile systems. We implement this architecture with single-stranded DNA (ssDNA) slats that polymerize from introduced DNA-origami or ssDNA seeds into monodisperse ribbons with average lengths in excess of 4 µm. Near-complete conversion of seeds into ribbons is achieved, with the number of seeds precisely controlling the ribbon copy number. Robust kinetic control of nucleation is sustained over a range of divalent-cation and free-slat concentrations at up to 10 °C below the reversible temperature for slat binding. Finally, we show the formation of ribbons with programmable twists and coils that can be assembled into tubes of different diameters.

## Results

### Theory

To explore how a monomer’s coordination number can be incremented to maintain the kinetic barrier to spontaneous nucleation while using irreversible growth conditions, we modeled the free energies of assemblies using different monomer designs with the kinetic Tile Assembly Model (kTAM)^6,8,11^. The half coordination number *n* for a given monomer is defined as the number of bonds it must make to be attached to an assembly using near-reversible reaction conditions (*ε* → 0). The barrier height to spurious nucleation corresponds to the free energy of the critical nucleus. Under irreversible growth conditions (i.e., *ε* > 0), the free energy of a critical nucleus is diminished by the excess bond energy, which is proportional to *ε*/*n* (Supplementary Discussion 2 and Supplementary Figs. 1–10). Therefore it is desirable to conceive of highly coordinated monomers and assemblies that minimize the excess bond energy.

**Fig. 1:**
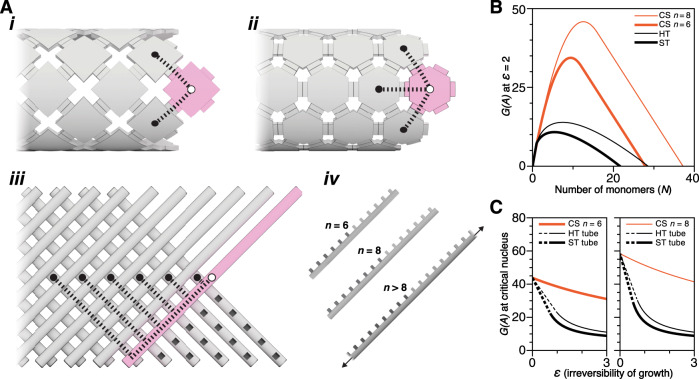
Monomers with a higher coordination number (2*n*) have more sustained kinetic barriers to spontaneous nucleation under increasingly irreversible growth conditions, with a theoretical comparison of square-tile (ST) nanotubes, hexagonal-tile (HT) nanotubes, and crisscross-slat (CS) ribbons that have identical free energies *G(A)* for nucleation at near-reversible *ε* → 0 conditions. **A** Under near-reversible-growth conditions, incoming ST (i) and HT (ii) bind two or three nearest neighbors respectively to elongate tubes, whereas incoming CS_*n*=6_ (iii) bind to six slats to elongate ribbons. (iv) Slats may be arbitrarily extended to any positive integer *n* to increase coordination number. **B** Free energy *G(A)* versus the number of *N* monomers in an assembly is larger for CS at irreversible *ε* = 2 conditions (i.e., 10^*ε*^ = 100:1 growth:shrinkage, “high” 1 µM monomer). **C** Larger free energies of nucleation *G(A)* for the slats are sustained at irreversible, fast-growth, high *ε* conditions compared to the tiles, with 1 µM monomer concentration. Dotted versus solid lines for ST and HT indicate where the critical nucleus transitions from a fixed size with a constant number of monomers to where the nucleus contains increasingly fewer monomers.

We first considered lower-coordination square tile (ST) and hexagonal-tile (HT) systems, where *n* = 2 and 3 respectively (Fig. 1Ai–ii). We modeled ST and HT nanotubes of comparable circumferences, i.e., the free energies of nucleation match for near-reversible growth (Supplementary Table 1). We set a “high” 1 µM concentration of each monomer (Supplementary Discussion 2.1) as a desirable benchmark to achieve seed-dependent assembly; prior experimental implementation of ssDNA ST nanotubes did not satisfactorily control spontaneous nucleation at this concentration (Supplementary Information A, Section S5.1.1 and Fig. S27A from Woods et al.^12^). The modeled free energies of assemblies where growth is fast and irreversible (*ε* = log_10_(100) = 2, rate of tile attachment is 100 times larger than the rate of tile detachment) are plotted in Fig. 1B (see also Supplementary Fig. 1B) with black lines. The barrier for HT is marginally higher than that for ST (i.e., 13.9 versus 10.8 energy units), thus the expected relative rate of spontaneous nucleation is about three orders-of-magnitude slower (i.e., 10^−(13.9−10.8)^) for HT than for ST under these growth conditions. Free energies of nucleation versus *ε* are plotted in Fig. 1C, where the kinetic barrier drops rapidly as growth conditions are increasingly irreversible. We conclude that the additional coordination site with HT versus ST monomers is insufficient to maintain large kinetic barriers for fast, irreversible growth conditions (Supplementary Discussion 2.1).

**Fig. 2:**
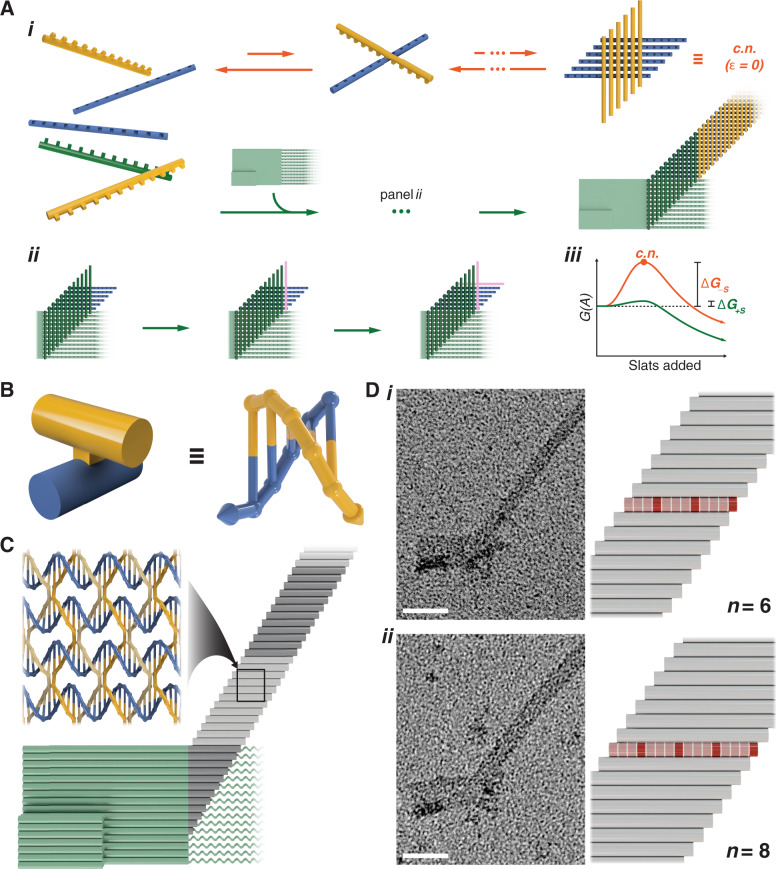
Crisscross polymerization as implemented with ssDNA slats. **A**i Ribbon assembly of abstract v6 x-slats (blue) and y-slats (gold). Upper pathway shows transient association of free slats, where the critical nucleus (c.n.) requires a multitude of unfavorable slat additions. Lower pathway uses a seed (light green) to capture twelve nuc-*y*-slats (dark green) along with six *x*-slats. **A**ii, Subsequent slats (magenta) are recruited in alternating perpendicular fashion for ribbon growth. **A**iii, Qualitative energy landscape for unseeded (orange) versus seeded (green) nucleation and growth of slats using irreversible growth conditions. **B** Each binding domain in the abstract model is a half turn of DNA. **C** Crisscross polymerization of ssDNA slats (blue *x*- and gold *y*-slats) as triggered by a DNA-origami seed (light green). Straight cylinders represent double helices. **D** Left are negative-stain TEM images of v6 (*n* = 6) and v8 (*n* = 8) ribbons, in (i) and (ii), respectively. Right models show width differences, where each boxed cell is a single half-turn domain. Light-red or dark-red cells correspond to either five or six base-pair domains. Scale bars are 50 nm.

To determine whether kinetic barriers to nucleation are sustained under fast, irreversible growth conditions using higher monomer coordination numbers, we conceived of crisscross slats (CS). CS can be extended arbitrarily to achieve any desired coordination number and are composed of a linear array of 2*n* weak-binding sites that polymerize into ribbons. Each binding site is specific to a single conjugate site on one of 2*n* distinct slats that are routed perpendicularly in another layer (Fig. 1Aiii–iv). There are two classes of slats: *y*-slats and *x*-slats in the top and bottom layers, respectively. Specificities are arranged so that alternating *y*-slats and *x*-slats add sequentially to the ribbon end by securing *n* consecutive cross-binding interactions, as shown by the magenta slat in Fig. 1Aiii (also see Supplementary Fig. 7). Slats achieve their coordination number by reaching beyond nearest neighbors (with respect to the center of mass of each slat), such as depicted for *n* = 6 in Fig. 1Aiii. Under near-reversible growth conditions, the critical nucleus is composed of *n* *y*-slats and *n* *x*-slats (Fig. 2Ai and Supplementary Fig. 8).

In Fig. 1C, the free energies of nucleation versus *ε* for CS are plotted in orange. We note that for each comparison, CS ribbons have the same free energies of nucleation as the aforementioned ST and HT nanotubes under near-reversible growth conditions (Supplementary Discussion 2). Importantly, the kinetic barrier for nucleation of CS diminishes at a much slower rate with rising *ε* values for either *n* = 6 or *n* = 8 slats (shown by the orange lines), in contrast to the depleted barriers for either ST or HT nanotubes (see also Supplementary Discussion 2 and Fig. S5). Indeed, in Fig. 1B (see also Supplementary Fig. 1B) the kinetic barriers for fast, irreversible growth (i.e., *ε* = 2) are 34.4 and 45.8 energy units for *n* = 6 and *n* = 8 slats respectively, which is much larger than either HT or ST. The expected relative spontaneous nucleation rates for *n* = 6 and *n* = 8 CS are 23.6 (i.e., 10^−(34.4−10.8)^) and 35 (i.e., 10^−^^(45.8−10.8)^) orders-of-magnitude slower compared to ST at this growth condition. Thus, the results from the kTAM comparison suggest that the higher coordination numbers in CS greatly lessen the degree to which the excess bond energy diminishes the kinetic barriers under irreversible reaction conditions. As such, we used the kTAM results as a qualitative motivation to create and test CS experimentally (Supplementary Discussion 2).

To rapidly bypass the kinetic barrier, polymerization of ribbons from the slats is triggered using a seed (light green) (Fig. 2Ai). Periodic polymerization of unique repeating *y*-slats (gold) and *x*-slats (blue) is initiated from the seed that must first recruit 2*n* unique nucleating *y*-slats (i.e., nuc-*y*-slats, dark green) and the first *n* *x*-slats (blue) with the appropriate geometrical arrangement (Fig. 2Aii).

### Crisscross implementation with ssDNA slats

We sought the most compact slat architecture to satisfy the requirements of crisscross polymerization as described above, and also enable economical use of the highest concentrations of monomers for the fastest growth. DNA has proven to be a versatile material for the self-assembly of nanostructures^41–45^. Here we report that an ssDNA, conceptualized as a linear array of half-duplex domains (i.e., 5 or 6 nucleotides (nt)), can serve well as a crisscross slat (Fig. 2A, B). According to our design, multiple ssDNA slats can assemble into crisscross ribbons comprised of staggered parallel double helices connected by antiparallel crossovers that occur every half turn (Fig. 2C). Single-stranded slats are designed to alternate threading over and under consecutive cross-binding partners, instead of passing entirely above or below as depicted in our abstract model of crisscross (cf. Fig. 2A versus Fig. 2C). Base stacking is designed to propagate along the *x* axis (i.e., parallel to the helices of the seed as in Fig. 2C), such that *x*-slats never cross over and therefore follow a right-handed helical path, whereas *y*-slats cross over every half turn and therefore follow a left-handed helical path (Fig. 2C). For this study, we experimentally examined crisscross growth from “v6” slats (CS_*n*=6_, Fig. 2Di) versus “v8” slats (CS_*n*=8_, Fig. 2Dii) (Supplementary Figs. 11 and 12).

We assembled a DNA-origami seed that presents single-stranded scaffold loops pre-organized to cooperate in recruiting the nuc-y-slats (i.e., 12 unique nuc-*y*-slat sequences for v6 and 16 for v8) needed to initiate the growth of periodic ribbons (Fig. 2C, and Supplementary Fig. 13). We designed multiple DNA slat sequence variants for v6 and v8 (Supplementary Table 2 and Supplementary Data S1). For v8, we also exploited symmetry for unique repeating sets of only *n* pairs of *x*- and *y*-slats sequences, or even only *n*/2 pairs, useful for reducing material costs (Supplementary Table 3).

### Characterization of ssDNA slat assembly

**Fig. 3:**
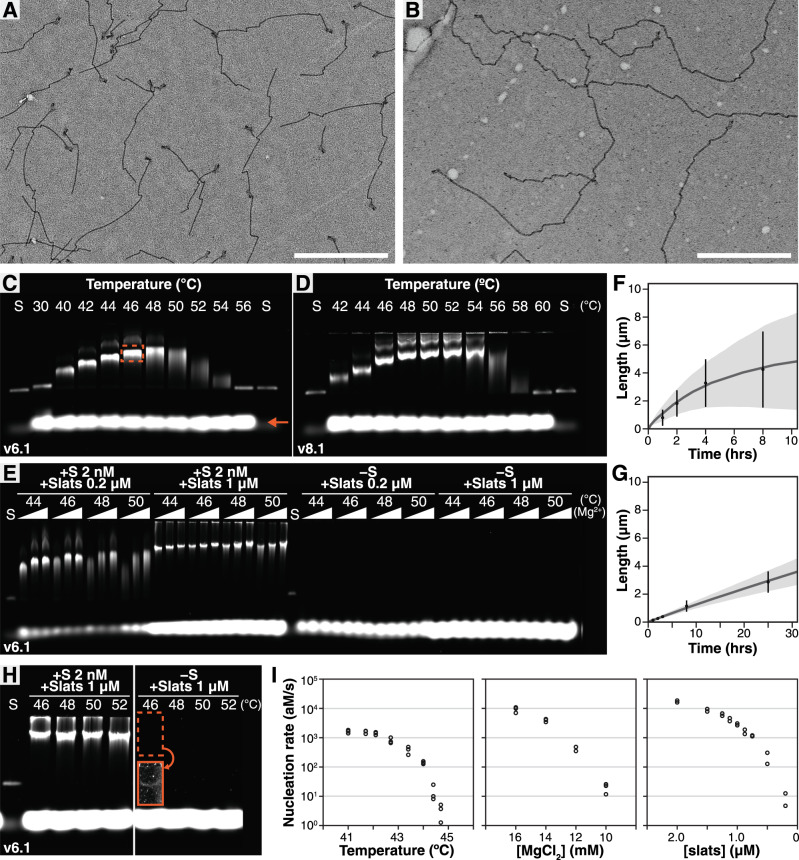
Characterization of DNA slat assembly with and without the seed. Negative-stain TEM images of v6.1 (**A** 2 nM seed, 1 µM each slat, 16 mM MgCl_2_, and 50 °C for 1 h) and v8.2 (**B** 2 nM seed, 0.5 µM each slat, 16 mM MgCl_2_, and 50 °C for ~16 h) seeded ribbons, with 1 µm scale bars. **C**, **D** Agarose gels of seeded v6.1 and v8.1 ribbons using 14 mM MgCl_2_, 0.2 µM each slat, and 2 nM origami seed. Dotted orange boxes are ribbons, orange arrow points to unpolymerized slats (each slat is in 100-fold excess to the seed in the reaction), and “S” is the origami seed. **E** Agarose gel of v6.1 slats incubated ~16 h (0.2 or 1 µM each slat and 2 nM origami seed), with the Δ showing 12, 14, or 16 mM MgCl_2_. **F**, **G** Length of seeded v6.1 ribbons versus time from TEM for a fast-growth high temperature and a slow-growth low temperature, respectively. Data points are the mean ± SD length (**F** *N*_1h_ = 163, *N*_2h_ = 130, *N*_4h_ = 141, *N*_8h_ = 140; **G** *N*_1h_ = 155, *N*_2h_ = 168, *N*_3h_ = 154, *N*_8h_ = 161, *N*_25h_ = 151; with error bars obscured by data points). Gray interpolating lines are mean lengths from the stochastic model (*N* = 150 ribbons, with shaded range ±SD). **H** Extended 100 h incubation of v6.1 slats. **I** Rate of spontaneous nucleation versus reaction parameters for v6.1. Results are representative of three independent experiments for temperature and MgCl_2_, and two independent experiments for slat concentrations.

We verified that seeds nucleated crisscross assembly with either v6 or v8 slats by observing ribbons with negative-stain transmission electron microscopy (TEM, Figs. 2D, 3A, B and Supplementary Figs. 14–16). We hypothesize that the kinked appearance of the ribbons is due to the trapping of twisted configurations on the TEM grids, consistent with the observation that ribbons programmed with higher bp/turn (e.g., 11 bp/turn versus 10.5 bp/turn) exhibit a very high degree of kinking. We used agarose gel electrophoresis to survey how temperature and divalent-cation (i.e., Mg^2+^) concentration influenced qualitative distributions of ribbon lengths. With slats at 0.2 µM each and the seed at 2 nM, we grew ribbons for 16 h using various v6 (v6.1, v6.2, v6.3; Fig. 3C and Supplementary Figs. 17, 18) or v8 slat sequence variants (v8.1, v8.2; Fig. 3D and Supplementary Figs. 19, 20). We define “optimal” growth conditions as those producing the longest ribbons with the most uniform length distributions, as evidenced by a tight, slowly migrating band on an agarose gel with respect to the origami seed “S” band (e.g., the orange boxed band in Fig. 3C). We also observe a minor population of a more slowly migrating species (i.e., higher on the gel) that resemble entangled ribbons after excision from the gel and imaging via TEM. The v6 slats in 12, 14, or 16 mM MgCl_2_ formed ribbons optimally at 44–50 °C, whereas v8 slats grew optimally at temperatures 48–54 °C. Monodispersity and total lengths of ribbons at a given condition differed among sequence variants, such as v6.1 and v6.3, which generally grew longer and more evenly than v6.2 (Supplementary Table 2). The copy number of filaments was controlled precisely by the number of starting seeds added to the reaction (Supplementary Fig. 21). Adding blocking strands sequestering one or more of the nuc-y-slats led to termination of assembly under certain growth conditions (Supplementary Fig. 22).

To measure the prevalence of spontaneously nucleated ribbons at the optimal growth conditions, we incubated slats overnight with no seed (44–50 °C, 0.2 or 1 µM each slat, 12–16 mM MgCl_2_, ~16 h growth). We estimated our limit of detection at ~65 pg per agarose gel lane, corresponding to a half-million 5 µm ribbons (assuming ~3300 slats contributing 1.5 nm extension each), or ~200 fM for a typical 4 µL reaction volume (Supplementary Fig. 23). Unseeded ribbons were undetectable on agarose gels for v6.1 and v6.2 (Fig. 3E and Supplementary Fig. 24). However, they were observed for select conditions for v6.3, indicating that sequence differences can influence nucleation behavior (Supplementary Figs. 25 and 26). Furthermore, v8.1 and v8.2 demonstrated no observable spontaneously nucleated ribbons (Supplementary Fig. 27). We experimentally ascertained that the reversible temperature for binding of the v6.1 and v6.2 slats is ~56 °C at 16 mM MgCl_2_ and 1 µM each slat (Supplementary Fig. 28). Remarkably, spontaneously nucleated ribbons were not observed following overnight incubation using reaction parameters conducive to rapid, irreversible growth of v6.1, v6.2, v8.1, and v8.2 slats. With v6.1 and v6.2 in particular, we further observed strictly seeded growth with 1 µM each slat using rapid, irreversible optimal growth conditions ~10 °C below the reversible temperature, where spontaneously nucleated ribbons were undetectable, i.e., below the 200 fM gel-detection limit.

Next, we measured the kinetics of slat assembly under different experimental conditions by measuring ribbon lengths in TEM micrographs (Supplementary Figs. 29–32). We characterized v6.1 with the optimized growth conditions noted above (i.e., 50 °C, 16 mM MgCl_2_, 1 µM each slat; Fig. 3F and Supplementary Discussion 3.1, Supplementary Fig. 29). A one-hour incubation yielded ribbons with a mean length of ~800 nm, and after 8 h the mean length exceeded 4 µm. We also examined the growth of v6.1 at a lower temperature and higher MgCl_2_ concentration outside the optimal range (40 °C, 20 mM MgCl_2_, 1 µM each slat; Fig. 3G and Supplementary Discussion 3.1, Supplementary Fig. 30). The rate of ribbon growth was much slower, with a mean length of ~140 nm after 1 h and a mean length of only ~3 µm after 24 h. In contrast to the faster growth noted above, these suboptimal slow-growing conditions were also especially prone to spontaneous nucleation of ribbons (Supplementary Discussion 3.2 and Supplementary Fig. 33). To account for how an interplay of nucleation, extension, and termination might describe our observations, we developed a stochastic model and derived an analytical solution for DNA slat assembly. By fitting parameters corresponding to a linear growth rate, nucleation rate, stalling probability, and termination probability, we found that our model is in general agreement with length measurements obtained from TEM images and gel data for both optimal and suboptimal growth conditions, with faster growth and termination kinetics at higher temperatures resulting in the non-linear growth profiles observed (Fig. 3F, G and Supplementary Discussion 3.1–5, Supplementary Figs. 29–35). Using the TEM length distribution data, we calculated polydispersity indices for seeded growth of different design versions and experimental conditions to fall in the range of 1.07–1.53 (Supplementary Discussion 3.1 and Supplementary Figs. 29–32). We expect a narrowing of polydispersity to be achievable in the future through further exploration of DNA slat sequences (Supplementary Table S2).

To challenge the robustness of the DNA slats to spontaneous nucleation, we incubated v6.1 and v6.2 for an extended 100 h period. We used the optimal growth temperatures (46–52 °C, 16 mM MgCl_2_, 1 µM each slat) and observed no unseeded ribbons for v6.2 (Supplementary Fig. 36). For v6.1, no unseeded ribbons were seen except for a faint band near the gel-detection limit at the lowest temperature tested, corresponding to ~200 fM ribbons or a spontaneous nucleation rate of ~0.6 aM/s (Fig. 3H, see boxed brightness and contrast adjusted gel section in orange at 46 °C). This rate is ~500-fold lower than previously reported for DNA tile assembly observed using slower, near-reversible growth conditions where a lower 0.2 µM per tile concentration was used, versus 1 µM as tested here^26^. We emphasize that spontaneous nucleation of the DNA slats is substantially lower (i.e., unobservable on agarose gel) when they are assembled using more desirable conditions yielding faster growth (see Supplementary Discussion 2.2 for further comparison to tiles). These gel results are consistent with expectations from the stochastic model as outlined in Supplementary Discussion 3.3. Although the rate of spontaneous nucleation was too low to study the extent to which it may exist at the optimal growth temperatures, we examined it further at lower temperatures where greater amounts of spontaneously nucleated ribbons were observed. We incubated v6.1 at suboptimal growth temperatures (34–44 °C, 16 mM MgCl_2_, 1 µM each slat, ~16 h growth) and observed increased occurrence of unseeded ribbons, albeit with progressively slower growth as the temperature was lowered (Supplementary Fig. 37A). We used unseeded two-step nucleation and growth protocol, where nucleation was induced at a lower temperature followed by a higher temperature favoring growth to make the ribbons attain similar lengths, to quantitatively assess spontaneous nucleation (Supplementary Fig. 37B–C). Increasing temperature (i.e., from 41.0 to 44.7 °C), decreasing the concentration of slats (i.e., from 2.0 to 0.2 µM), or decreasing MgCl_2_ (i.e., from 16 to 10 mM) could each individually decrease the rate of formation of spontaneous nucleation ~1000 fold (Fig. 3I and Supplementary Fig. 38). Furthermore, we found that v6.2 was particularly resistant to spontaneous nucleation with ~100-fold lower nucleation rate compared to v6.1 at any given temperature (Supplementary Fig. 39, also see Supplementary Table 2 where sequence differences are explained). We hypothesize that the changes to experimental parameters as explored at suboptimal conditions might be applied to lessen spontaneous nucleation at faster growth conditions—however, the low number of spontaneously nucleated ribbons in question is not observable using the detection limit of agarose gels as explored in this work.

To illustrate how to crisscross polymerization could be seeded from an unstructured ssDNA (i.e., not a pre-formed DNA-origami) and therefore be used for detection of a real-world analyte, we designed a set of six nuc-*x*-slats that folds a 190 nt segment of a ssDNA viral genome (M13 in this case); this structure then can bind six *y*-slats and thereby form a nucleus for periodic v6 ribbon assembly (Supplementary Fig. 40). For smaller analytes with insufficient binding energy to recruit six nuc-*x*-slats to high local concentration, linking crisscross growth to target detection will likely require a different approach. Further discussion of crisscross in the context of other enzyme-free detection methods can be found in Supplementary Discussion 1.

### Tubular structures

**Fig. 4:**
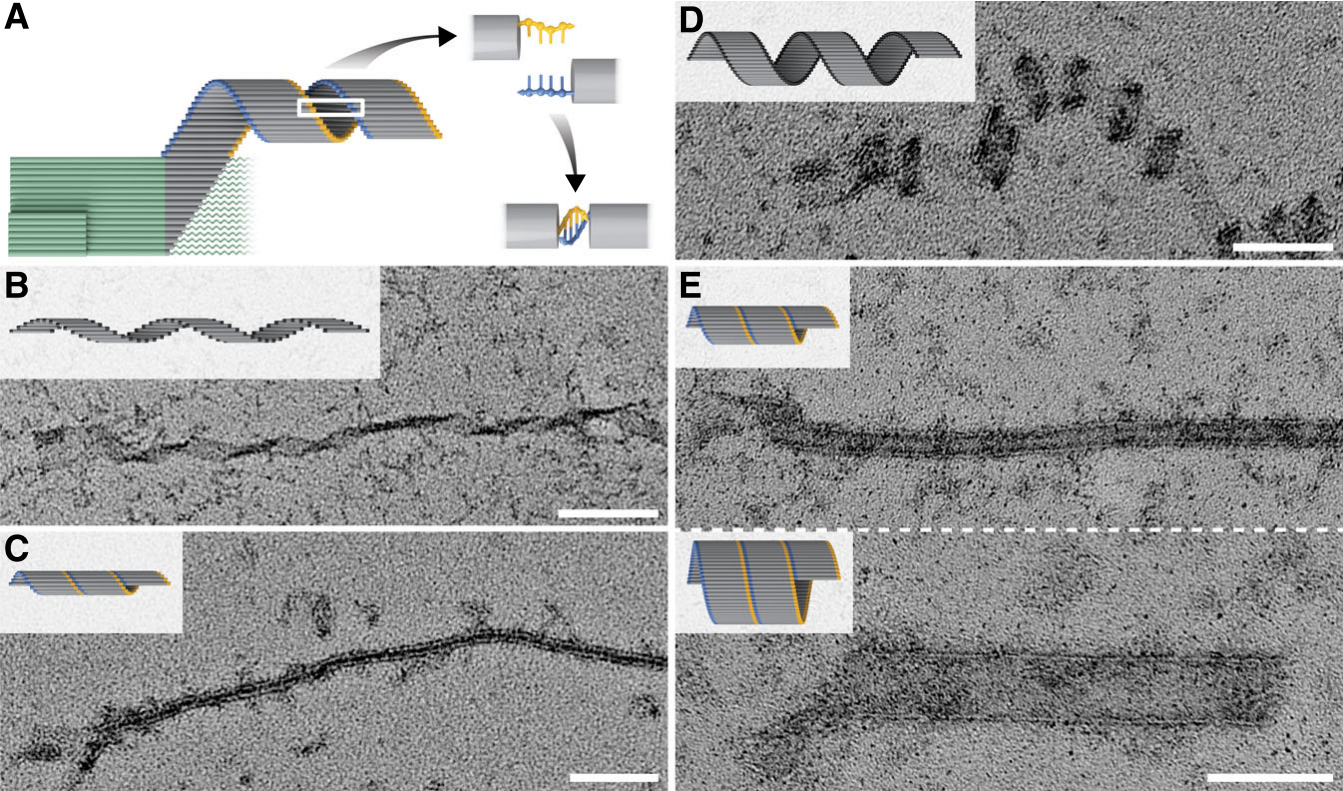
Formation of tubes and coiled ribbons. **A** Seeded growth of a coiled ribbon. Coiled ribbons can be closed with sticky-end overhangs, as indicated with gold and blue. **B** v8.3 slats without sticky ends form open, tightly coiled ribbons. **C** v8.3 slats with 3 nt sticky ends form closed tubes of constant diameter. **D** v8.1 slats without sticky ends form open and loosely coiled ribbons (note the difference from the coiled ribbon in **B**). **E** v8.1 slats, with 4 nt sticky ends, form closed tubes of varying diameters in a single reaction. Scale bars are 100 nm.

A diversity of ribbon shapes could prove useful for various applications such as multiplexed detection and nanofabrication. Therefore we further generalized crisscross morphology by designing self-assembly of CS ribbons that coil into tubes (Fig. 4). To achieve this, we tested v8 slat variants where either the pattern of 5 and 6 nt domains is shifted, or the number of base pairs per turn for each four-unit set of domains is increased (i.e., from 10.5 bp/turn to 11 bp/turn; Supplementary Fig. 41). Shifting the arrangement of the domains in v8.1 slats, while maintaining a 10.5 bp/turn reciprocal twist, led to loose coiling of the ribbons (Fig. 4D and Supplementary Fig. 42B). Underwinding the DNA to 11.0 bp/turn in v8.3 slats led to more tightly coiled ribbons (Fig. 4B and Supplementary Fig. 42C). We added short sticky ends to the slats to increase the propensity of closure of the coiled sheets into tubes of varying diameters (Fig. 4C, E and Supplementary Figs. 43, 44). Interestingly, v8.3 slats exhibited an especially rapid rate of addition (estimated second-order rate constant 10^6^ M^−1^ s^−1^ for v8.3 versus 0.5 × 10^6^ M^−1^ s^−1^ for v6.1; Supplementary Fig. 45 and Supplementary Table 4) and resulted in a constant narrow-tube diameter, while maintaining no observable spurious nucleation under optimal growth conditions (Supplementary Fig. 46).

## Discussion

Crisscross polymerization combines the unbounded size of DNA brick or tile nanoconstructions with the fast-folding and copy-number control of DNA-origami by delivering robust nucleation control over a wide range of temperatures and slat concentrations. Similar to past developments with other motifs in structural DNA nanotechnology, crisscross assembly with ssDNA slats should prove adaptable to 2D and 3D growth architectures^1,2,41,42^. Because of the generalizability of crisscross from a theoretical perspective, we foresee that crisscross will prove amenable to being adapted to monomers other than ssDNA, wherever linear arrangements of weak, specific binding sites can be synthesized. We envision that crisscross assembly will be broadly enabling for all-or-nothing formation of two- and three-dimensional microstructures with addressable nanoscale features, algorithmic self-assembly, and zero-background signal amplification in diagnostic applications requiring extreme sensitivity.

## Methods

### DNA slats

DNA slat sequences were designed using custom Python scripts; base-pairing energies were assessed using Unafold^46^. All sequences were designed to have minimal self-structure. The v6.1 slats were designed to have stronger base-stacking in the *y* direction than in the *x* direction, as assessed using Unafold. The v6.3 and v8.1 slats were designed to have stronger base-stacking in the *y* direction than in the *x* direction, based on stacking energies reported in Protozanova et al.^47^. Note about poly-thymidine (poly-T) brush passivation for ribbon growth: Poly-T brushes on the 5′ and/or 3′-ends of select slats in a given slat sequence design were necessary to passivate the ribbons. Ribbons formed from slats where brushes were not added tended to aggregate into larger clumps as noted by TEM (see v8.7 in Supplementary Fig. 16 as an example). We hypothesize that ribbons with no poly-T brushes associate non-specifically with one another by blunt end stacking. In all of the v6 slat designs, the poly-T brush was designed into the commercially purchased slat sequence as noted in Supplementary Table 5. For v8.1 and v8.2 slat designs that formed ribbons, we enzymatically added 3′-end poly-T brushes as described in the methods below and in Supplementary Table 5. For other v8 slats used to form tubes, aggregation was generally less prevalent because the designed twist/tube morphology concealed the aggregation-prone edges of the ribbon within the assembled tube.

### DNA slats denaturing polyacrylamide gel electrophoresis (PAGE) purification

Unpurified dehydrated DNA slat oligonucleotides were purchased from Integrated DNA Technologies (IDT) at 10 or 100 nmol scales. SequaGel UreaGel System (National Diagnostics, EC-833) reagents were used to prepare 15% denaturing PAGE gels in empty plastic 1.5 mm mini-gel cassettes (Invitrogen Novex™, NC2015). Each unpurified DNA slat was rehydrated at 100 or 700 μM (assuming 70 nmol/well for 100 nmol dry IDT oligo order) in Milli-Q water, slats were combined into pools (for nuc-*y*-slats, *x*-slats, and *y*-slats, respectively), and pools were mixed 1:1 by volume with 95% formamide, 0.025% (w/v) bromophenol blue, 0.025% (w/v) xylene cyanol, and 5 mM EDTA. Samples were loaded into the gels and run at 200–300 V for ~40–50 min until the bromophenol blue dye front had run off the gel. Bands were identified on the gels by shadowing with UV light so that bands for each pool could be excised with a razor blade. Gel slices were crushed in a 2 mL round-bottom tube with a pestle, soaked at room temperature on a shaking incubator at 1500 rpm in an excess of 1X TE buffer (5 mM Tris and 1 mM EDTA) overnight, at which point waste acrylamide was separated from the aqueous slats solution with Freeze N’ Squeeze spin columns (Bio-Rad, 732-6166). The DNA slats were further purified by precipitation in isopropanol, two washes in cold 70% ethanol, and final resuspension in water, with the volume sufficient to attain an approximate concentration of 10 μM per each slat in the pool. A Nanodrop 2000c Spectrophotometer (ThermoScientific™) was used to determine the final concentration. DNA slats v6.2 in Supplementary Fig. 32 were double PAGE purified (i.e., performing the PAGE purification noted above twice). All DNA slat sequences are reported in Supplementary Data 1.

### Agarose gel electrophoresis

Gel characterization of the origami seed or assembled slat filaments was performed using either the ThermoScientific™ Owl™ EasyCast™ B2 or D3-14 electrophoresis system. UltraPure agarose (Life Technologies, 16500500) was melted in 0.5X TBE (45 mM Tris, 45 mM boric acid, 0.78 mM EDTA, 12–16 mM MgCl_2_ to match amount in slats experiment) to a concentration of 0.5–2.0 % (w/v). The 0.5–1% gels were typically used to characterize larger assemblies of slats, whereas 2% gels for smaller structures such as the origami seed. The molten agarose was cooled to 65 °C and gel stain was added. All slat assembly reactions were characterized with 10,000× SYBR-Gold (ThermoFisher, S-11494) gel stain added to a final concentration of ~0.183× (i.e., 3 µL per 160 mL molten agarose). Origami seed folding was characterized with 6.25 × 10^−5^ % (w/v) ethidium bromide (i.e., 10 µL per 160 mL molten agarose; Bio-Rad, 1610433). Gels were covered with aluminum foil during solidification and running to lessen exposure to ambient light. DNA assemblies were mixed in an excess of agarose gel loading buffer (5 mM Tris, 1 mM EDTA, 30% w/v glycerol, 0.025% w/v xylene cyanol, 12–16 mM MgCl_2_; with typically 10 µL loading buffer added to 4–10 µL of each assembly). The mixed samples were loaded onto the gel and separated for 3–4 h at 60 V at room temperature. Control samples for size and densitometry included one or both of the following: first, 0.5–11.2 fmol of folded DNA-origami seed; or second, 0.00625–0.5 μg of Gene Ruler 1 kb Plus DNA Ladder (ThermoScientific™ SM1331). Gel images were captured on a GE Typhoon FLA 9500 fluorescent imager using the SYBR-Gold parameters as given in the Typhoon FLA 9500 Control Software (Version 1.1 Build 1.1.0.187). The photo-multiplier tube (PMT) was set to 300–500 V, with the value varied depending on the amount of sample loaded. Densitometry to quantify relative assembly of DNA bands was performed with ImageJ (v2.0.0-rc-69/1.52i). Background subtraction with a rolling ball radius of 30–60 pixels was performed on linear TIFF images. The GelAnalyzer plugin in ImageJ and wand tool was used to integrate total pixel intensities from lanes of interest. Gel absorbency data collected on different agarose gels were compared to one another by normalizing them with respect to the DNA-origami seed control, as well as the volume of reaction loaded.

### DNA-origami seed

Unpurified dehydrated staple oligonucleotides were purchased from Integrated DNA Technologies (IDT) at 10 nmol scale. Each unpurified staple strand was rehydrated at ~100 μM in Milli-Q water and then equal volumes pooled together. The p8064 scaffold strand was produced from M13 phage replication in *Escherichia coli*. The DNA-origami seed was folded with 10 nM p8064 scaffold and ~100 nM of each unpurified staple strand in 1X TE buffer (5 mM Tris and 1 mM EDTA) containing 6 mM MgCl_2_. The reaction was incubated on a PTC-225 Peltier Thermal Cycler (MJ Research) with the following temperature program: 90 °C for 2 min, cool to 55 °C and decrease to 50 °C over 18 h by decreasing the temperature at a rate of −1 °C/3 h, and then holding the temperature at 4 °C thereafter. The folded reaction was analyzed on an agarose gel and by observation with TEM. The folded seed was stored at 4 °C in the raw folding mixture. The seed was noted to remain stable in 4 °C storage for up to a year and was used directly from the raw folding reaction in slat assembly experiments. Staple and scaffold DNA sequences are reported in Supplementary Data S1.

### Ribbon and tube assembly reactions for slats with *n* = 6 domains, or *n* = 8 domains

10X reaction buffers were prepared for all reactions as follows: 35 mM Tris, 7 mM EDTA, 0.1% Tween-20 (Sigma Aldrich #P9416), 108–200 mM MgCl_2_ depending on the final MgCl_2_ in the reaction. The use of 10X reaction buffers was preferred to lessen variability in the final concentration of MgCl_2_ and Tween-20 between experiments. PAGE purified DNA slat pools and the DNA-origami seed were added to diluted reaction buffer to achieve the final concentrations: 0.2–1 µM purified DNA slats, 0.003–2 nM seed, and 1× reaction buffer. It is noteworthy that PAGE purification of the slats was critical to achieve filament growth. Attempts to assemble the raw slats as purchased from Integrated DNA Technologies (IDT) did not result in appreciable growth. We hypothesize that impurities in the raw slats such as 5′-end truncations (i.e., slats with an incomplete number of binding domains) were incorporated into the leading edges of the growing ribbons, and then prevented from falling off by the addition of a few subsequent slats. If multiple such truncations become trapped at the end of a ribbon, this should lead to termination of growth. The reactions were assembled isothermally on a PTC-225 Peltier Thermal Cycler (MJ Research) or a Tetrad 2 Peltier thermal cycler (Bio-Rad) at a suitable growth temperature (e.g., 46–56 °C) for various times (e.g., 1–100 h, but overnight ~16 h was most typical). Although spurious nucleation occurring during room-temperature preparation of the nuc-*y*-, *x*-, and *y*-slats was not typically observed, we performed variations of the protocol for when spontaneous nucleation and growth would have otherwise skewed the results. In one approach, the seed was not initially added so the slats could be denatured at 85 °C for 10 min, at which point the seed was added to the now-cooled 60 °C reaction. In the second approach, *x*-slats were not added, the reaction was incubated at 60 °C for 3–4 min, at which point the *x*-slats were added. In both approaches, the reactions were subsequently cooled to the final isothermal growth temperature.

### Enzymatic addition of poly-T brushes for v8.1 and v8.2 on 3′-ends of slats

Terminal transferase (TdT) was used to synthesize a poly-T brush on the 3′-end of each v8 slat. TdT was used here because slats as-purchased were 90 nt and further increasing their length for the brush would have resulted in a significant upcharge from the commercial vendor. The TdT poly-T brush reaction were as follows: PAGE purified *x*- and *y*-slats were separately combined with dTTPs (Fisher Scientific, R0171) at a molar ratio of 1:125, 0.05 v/v% terminal transferase (NEB, M0315L) in 1× terminal transferase reaction buffer (NEB, M0315L) and 0.25 mM CoCl_2_ (NEB, M0315L). The reaction was incubated for 30 min at 37 °C, followed by heat inactivation of the enzyme at 70 °C for 10 min. The slats were then separated from the reaction buffer by precipitating them in isopropanol, washed twice in cold ethanol, and resuspending them in the water at the desired concentration. The DNA slat assembly reactions were then prepared as described above.

### Two-step incubation of v6 slats to observe spontaneous nucleation and measure nucleation rate

Reactions were prepared as in the preceding “Ribbon and tube assembly reactions for slats with *n* = 6 domains, or *n* = 8 domains” method. Then, reactions were incubated at a low temperature (i.e., 4–46 °C for 6–12 h) to promote spurious nucleation. Next, they were incubated at a higher spurious nucleation-resistant and growth-favorable temperature (i.e., 50 °C for 10–16 h) to equalize lengths of the ribbons for the various conditions tested, so that quantitative comparison of spontaneous nucleation could be made. Initial nucleation and final growth conditions for all two-step incubation experiments were as follows: [Fig. 3I leftmost and S38A: (1 µM each slat, 16 mM MgCl_2_, 41–45 °C, 12 h) → (1 µM each slat, 16 mM MgCl_2_, 50 °C, ~12 h)]; [Fig. 3I middle and S38B: (1 µM each slat, 10–16 mM MgCl_2_, 40 °C, 9.7 h) → (0.75 µM each slat, 16 mM MgCl_2_, 50 °C, ~16 h)]; [Fig. 3I rightmost and S38C: (0.2–2 µM each slat, 16 mM MgCl_2_, 40 °C, 6.15 h) → (1 µM each slat, 16 mM MgCl_2_, 50 °C, ~14.1 h)]; [Supplementary Fig. 38B: (1 µM each slat, 16 mM MgCl_2_, 4–49 °C, 6 h) → (1 µM each slat, 16 mM MgCl_2_, 50 °C, 10 h)]; [Supplementary Fig. 39: (1 µM each slat, 16 mM MgCl2, 4–44.3 °C, 6 h) → (1 µM each slat, 16 mM MgCl_2_, 50 °C, 17 h)]. For Fig. 3I middle and rightmost, buffer containing additional MgCl_2_ or heat-denatured slats was added immediately as the second growth phase of the experiment was started to equalize their concentration to promote the growth of the nuclei into ribbons of similar final length. Initial reactions and slat normalization buffers for Fig. 3I rightmost in particular required small variations in volumes of slats, such that an Echo 525 acoustic liquid handler was used to eliminate possible manual pipetting error. The nucleation rate for unseeded conditions where ribbons were observed was determined by comparing the gel intensity with respect to a seeded control (i.e., the seed concentration was known) assembled only for the latter growth normalization phase. It should be reiterated that measurement of spontaneous nucleation is limited by the gel-detection limit to observe ~500,000 ribbons (Supplementary Fig. 23)—as such, low levels of spontaneous nucleation that may have occurred at optimal ribbon growth temperatures (i.e., ~46 to ~58 °C) could not be determined.

### Transmission electron microscopy (TEM)

Filament samples were prepared by diluting slat assembly reactions 1:10–1:200 in 1× reaction buffer matching the buffer in the initial assembly reaction. The DNA-origami seed was diluted in 1× TE buffer with 6 mM MgCl_2_. FCF400-CU-50 TEM grids (Fisher Scientific, 5026034) were negatively glow discharged at 15 mA for 25 s in a PELCO easiGlow. The diluted sample (3 µL) was applied to the glow discharged grid, incubated for 2 min, and wicked off completely into Whatman paper (Fisher Scientific, 09-874-16B). Immediately after, 3 µL of 2% aqueous filtered uranyl formate, which in certain instances was pH adjusted with 25 mM sodium hydroxide, was applied and incubated for 1–2 s. The uranyl formate was wicked off completely into Whatman’s paper. All imaging was performed at 80 kV on a JEOL JEM 1400 plus microscope and captured with AMT Image Capture Engine Software Version 7.0.0.255. Adobe Illustrator 2019 was used to adjust the contrast of the TEM micrographs.

### Measurements and statistics

TEM filament length measurements were obtained manually using the “segmented line” tool in ImageJ (v2.0.0-rc-69/1.52i). At each condition of interest, lengths of 130–168 filaments were recorded from TEM images showing distinguishable single filaments where the origami seed was identifiable.

### Statistics and reproducibility

All agarose gel results used for quantitative analysis were performed in independent triplicate experiments unless otherwise noted in the figure caption. Qualitative agarose gel results and representative micrographs showing crisscross ribbon assembly were independently reproduced at least two times attaining similar results unless otherwise noted in the figure caption.

### Reporting summary

Further information on research design is available in the Nature Research Reporting Summary linked to this article.

## Supplementary information

Supplementary Information

Description of Additional Supplementary Files

Supplementary Data 1

Reporting Summary

## Data Availability

Data supporting the findings of this manuscript are available from the corresponding authors upon reasonable request. A reporting summary for this Article is available as a Supplementary Information file. Source data are provided with this paper.

## Code availability

The Python code for generating the DNA slat sequences and the code for the stochastic model are available at https://github.com/aersh/crisscross.
