# Supplementary material for: Robust nucleation control via crisscross polymerization of highly coordinated DNA slats
Source: Nat Commun. 2021 Mar 19;12:1741. doi: 10.1038/s41467-021-21755-7 (PMC7979912; doi:10.1038/s41467-021-21755-7)
Supplement: Supplementary file 1 — Supplementary Information [file 41467_2021_21755_MOESM1_ESM.pdf]

Supporting Information

Title:

Robust nucleation control via crisscross polymerization of highly coordinated DNA slats

Authors:

Dionis Minev[1,2,3,4,#], Christopher M. Wintersinger[1,2,3,4,#], Anastasia Ershova[2,3,4], William M. Shih[2,3,4,*]

Affiliations:

[1]John A. Paulson School of Engineering and Applied Sciences, Harvard University, 29 Oxford Street, Cambridge, MA 02138, USA; [2]Wyss Institute for Biologically Inspired Engineering at Harvard University, 3 Blackfan Cir, Boston, MA 02115, USA; [3]Department of Cancer Biology, Dana-Farber Cancer Institute, 360 Longwood Ave, Boston, MA 02215, USA; [4]Department of Biological Chemistry and Molecular Pharmacology, Harvard Medical School, 240 Longwood Avenue, C-213 Boston MA, 02115, USA; [#]denotes equal contribution, *Correspondence: William_Shih@dfci.harvard.edu

Table of contents

Supplementary Discussion 1 **3**

Supplementary Discussion 2 **5**

2.1: Comparison of CS ribbons versus ST and HT nanotubes 5

2.2: Comparison of ST zigzag ribbons versus STN, HTN, and CS ribbons 12

2.3: Toy model for building intuition on why designs with larger n have smaller Bε/n 14

2.4: In our simplistic kTAM model, how does lowering the temperature affect the barrier height? 16

Supplementary Discussion 3 **17**

3.1: Kinetics by TEM observation versus model fit 19

3.2: Observation of spurious ribbons on agarose gels versus model fit 21

3.3: Analytical solution of ratiometric gel analysis 23

3.4: Analytical solution derivation 23

3.5: Consideration of delayed seed initiation 25

Supplementary Figures 1–46 **26**

Supplementary Figures 1–10: Principles of crisscross-slat versus square-tile and hexagonal-tile nucleation 26

Supplementary Figures 11–13: ssDNA slat architecture 38

Supplementary Figures 14–22: Determining optimal growth conditions for seeded ribbons 41

Supplementary Figures 23–28: Assay for spontaneous nucleation under optimal growth conditions 50

Supplementary Figures 29–35: Ribbon assembly characteristics by experimental observation and modelling 56

Supplementary Figures 36–39: Quantification of spontaneous nucleation under suboptimal low-temperature growth conditions 63

Supplementary Figure 40: Crisscross polymerization to sense nucleic acid sequences 67

Supplementary Figures 41–46: Twisted ribbons and tubes 68

Supplementary Tables 1–5 **74**

Supplementary References **79**

Supplementary Discussion 1

Further discussion on challenges for implementing kinetic trapping, especially for diagnostic amplification applications: Binding sites on monomers are per design occluded in metastable aggregates or by non-covalent intramolecular bonding and require an initiator to unlock the formation of a higher-order assembly[1–11]. More generally, the height of the kinetic barrier is determined by the energetic penalty of opening a single trapped monomer, or else of displacing a monomer bound in a trapping complex, to induce polymerization. Consequently, to increase that barrier, the kinetic trap of the monomer has to carry a larger energetic penalty for opening it. This would require changing the structure of the monomer, which is often not trivial. Furthermore, if the barrier is very large, then the time it takes to induce the release of the kinetic trap by the living polymer end may become rate-limiting for growth at high concentrations of monomer.

Single-stranded DNA (ssDNA) can be designed as a programmable monomer in living supramolecular polymerizations[12–16]. In the absence of an initiator, kinetically trapped hairpins are inhibited from polymerizing into thermodynamically favored chains. In the presence of unpaired ssDNA initiators, hybridization chain reactions (HCRs) can be triggered by opening hairpins using toehold-mediated strand displacement. Furthermore, catalytic hairpin assembly (CHA) can be programmed to produce initiator-dependent complex behaviors. However, HCR and CHA face some challenges. The second order rate constant for assembly for the original Dirks and Pierce HCR system (toehold = 6 nt, stem = 18 nt) was reported to be only 2×10^3 $M^{-1}s^{-1}$, or about a thousand-fold slower than the rate of unimpeded DNA hybridization, although a system with a stem of only 12 nt in length was reported to be ten times faster[16]. Growth appears to be highly sensitive to chemically imperfect hairpins, thus terminations are frequent, degrading monodispersity[16].

HCR and CHA have been investigated as platforms for enzyme-free amplification[14], which has the advantage that it bypasses the need for costly enzymes and the need for their environmental preservation (e.g. cold chain). However, another major concern is that leakage appears to be a persistent issue for HCR and CHA, which can be ameliorated somewhat by design[16,17]. As a result, the limit of detection (LOD) for strategies based on HCR or CHA typically has been reported in the pM to fM range, although some results in the aM range have been reported using electrochemical detection[14,18,19]. Towards achieving zM sensitivity, it is likely that the presence of trace truncated strands that act as initiators will be problematic. These will be present due to incomplete purification or else will arise continuously due to low-level

spontaneous cleavage. Thus HCR and CHA face challenges for rapid detection down to extremely low concentrations of analytes.

Another exciting direction for enzyme-free amplification is entropy driven cascades, especially with the recent development of "leakless" strand displacement systems[20]. Nevertheless, these systems also are susceptible to leak from truncated strands and complexes. Furthermore, strand displacement systems generally are not amenable to growth of distinct structures, and therefore are not as appropriate for digital counting of target analyte molecules, unless compartmentalization is employed.

In contrast, crisscross assembly is fast (second-order rate constant of at least 10^6 $M^{-1}s^{-1}$ thus far), and it would seem that there is no mechanism by which truncated strands should be expected to lead to spurious nucleation (although they can contribute to unwanted termination). We demonstrate that, in theory, an arbitrarily high nucleation barrier can be constructed, that only can be bypassed by a designed seed (i.e. not by truncated species); our experimental data shows a 200 fM LOD (i.e. limit of agarose-gel detection) under extremely irreversible conditions. According to our theoretical analysis, $G(A) = nG_{mc}/(1 + \varepsilon/G_{mc})$ (Supplementary Discussion 2), therefore spurious nucleation is predicted to slow by 11.4 orders of magnitude in going from 1.0 µM each slat (i.e. G_{mc} = 7.3) and 50-fold growth:shrinkage (i.e. ε = 1.7) — approximating the fast, irreversible growth conditions used for our measurement of 200 fM LOD — to 0.2 µM each slat (i.e. G_{mc} = 8.0) and 2-fold growth:shrinkage (i.e. ε = 0.3) — an example of slow, near-reversible growth conditions. Furthermore, each increase in monomer half coordination n is predicted to lead to almost a million-fold lower rate of spurious nucleation at 1 µM each slat (i.e. G_{mc} = 7.3), 50-fold growth:shrinkage (i.e. ε = 1.7). A discrete structure is produced per seed, thus crisscross assembly is conducive to digital counting without the need for compartmentalization. Crisscross should also be adaptable to polynomial or exponential growth for digital detection at extremely low analyte concentrations (e.g. low magnification, low-cost fluorescence microscopy).

Supplementary Discussion 2

Theoretical comparison of crisscross versus square-tile and hexagonal-tile assembly: In this section we adapt the previously developed kinetic tile assembly model (kTAM)[21–23] reported for square tile (ST) to hexagonal tile (HT) and crisscross (CS) assembly. In **Supplementary Discussion 2.1**, we present an in-depth description (a more technical description of the findings presented in the main text) of the theoretical results. It is noteworthy that ST assembly can also achieve flat ribbon formation with zig-zag ribbon assembly[24,25], and we compare the theoretical/measured nucleation rates of zig-zag ST to that of CS in **Supplementary Discussion 2.2**. In **Supplementary Figure 2**, we show the formation of various sized critical nuclei for all three assembly systems. We then show in **Supplementary Figures 3−4** how the critical nuclei for each system form into a stable and growing nanotube or ribbon. In addition to the discussion of the main text, we show in **Supplementary Figure 5** how the critical nucleus for each system shrinks with increasingly large values of ε. In **Supplementary Discussion 2.1** all equations and assumptions used in the comparison are highlighted. We also introduce a toy model for building intuition on how large n leads to small $B\varepsilon/n$, in **Supplementary Discussion 2.3**.

We note that the kTAM comparison of CS ribbons versus ST/HT nanotubes does not consider the potential for internal degrees of freedom within monomers, nor does it consider different binding domain sequences (i.e. DNA slats or tiles are modelled as rigid slats, squares, or hexagons without specific sequence information of binding domains). Furthermore, alternative assembly pathways resulting in nuclei spawned from misbonded—e.g. kinetically trapped or otherwise aggregated arrangements—of slat/tiles are also not considered in the kTAM comparison.

2.1: Comparison of CS ribbons versus ST and HT nanotubes

We first pursued a theoretical comparison of spurious nucleation between square-tile (ST) nanotubes and hexagonal-tile (HT) nanotubes (**Supplementary Figures 1–10**). We define the monomer half coordination number n as the number of bonds an incoming tile must complete for stable attachment under near-reversible growth conditions, equal to half the number of its coordination sites; $n = 2$ and 3 for ST and HT, respectively. We adapted the previously reported kinetic Tile Assembly Model (kTAM)[21–23] to calculate the energies of compact assemblies corresponding to potential critical nuclei. The free energy $G(A)$ of an assembly A is

$$G(A) = (nN - B)(G_{mc}/n) - \boldsymbol{B(\varepsilon/n)}$$

where N is the number of monomers, B is the number of pairs of bonded coordination sites, and $nN - B$ is the number of pairs of unbonded coordination sites. G_{mc} is the free-energy “debit”, mostly due to loss of translational and rotational entropy, for initiating capture of a free monomer from bulk solution into an assembly. The potential free energy per pair of unbonded coordination sites is $(G_{mc} + \varepsilon)/n$, where G_{mc}/n can be conceived as the destabilizing “debit” per pair of unbonded coordination sites, while $-\varepsilon/n$ can be conceived as the stabilizing “excess credit” per pair of bonded coordination sites (i.e. $\varepsilon \rightarrow 0$ at the temperature close to reversibility). We can conceptualize the equation above as the *free energy of assembly A* equals the sum of the *destabilizing* <u>*debits*</u> *from its unbonded coordination sites* and the ***stabilizing excess*** <u>***credits***</u> ***from its bonded coordination sites***. The term $(nN - B)(G_{mc}/n)$ is analogous to the interfacial free energy in classical nucleation theory (CNT) for a 2D assembly, as it describes the interface of the critical nucleus to the outside and is roughly proportional to the circumference of that 2D critical nucleus. $B(\varepsilon/n)$ is analogous to the bulk energy in CNT for a 2D assembly, which roughly scales with the area of the 2D critical nucleus.

For both ST and HT nanotubes, we define the programmed width k_{max} as the number of tiles across the circumference of the compact assembly that is the critical nucleus for $\varepsilon \rightarrow 0$. This appears to be a natural definition for comparing the two designs, since for any such ST or HT compact assembly of width k, $(nN - B)(G_{mc}/n) = (nk)(G_{mc}/n) = kG_{mc}$ (**Supplementary Table 1**). Thus for near-reversible-growth conditions, a large barrier to spurious nucleation can be achieved for either system by programming the tile sets to assemble into nanotubes of sufficiently large circumference. On the other hand, if the goal is to choose the system that better maintains its large kinetic barrier to assembly under fast, irreversible growth conditions (i.e. low G_{mc} and high ε), then the design that exhibits the smaller $B\varepsilon/n$ at every width k should be preferred. In **Supplementary Discussion 2.3**, we present a toy model that provides a partial explanatory framework to account for why, more generally, designs with larger n require fewer reversible-growth credits $-BG_{mc}/n$ to hold together monomers in a compact assembly of equivalent width k, and therefore bear fewer excess credits $-B\varepsilon/n$.

Comparing HT to ST compact assemblies of equivalent width, B is no more than 12.5% larger for HT (i.e. is very similar), therefore the reversible-growth credits $-BG_{mc}/n$ holding the metastable assembly together, as well as the excess credits $-B\varepsilon/n$, are from 29–33% smaller in magnitude for HT (**Supplementary Table 1**). In **Supplementary Figures 1B** and **1C**, we plot theoretical comparisons assuming a “high” 1 μM concentration of each monomer (corresponding to G_{mc} = 7.3) as a desirable benchmark for which to

achieve nucleation control; prior experimental implementation of square ssDNA tiles did not achieve satisfactory nucleation control using this concentration of monomers (see Supplementary Information A, Section S5.1.1 and Supplementary Figure 27A from Woods et al. [26]). Below a transitional threshold for *ε* (**Supplementary Figure 1C**, dotted black lines), the compact assembly with a width of k_{max} is the critical nucleus, and *G(A)* falls at a constant rate as *ε* rises. After *ε* passes its transitional value (**Supplementary Figure 1C**, solid black lines), the slope of *G(A)* versus *ε* flattens as successively smaller compact assemblies take over the role of critical nucleus. For fixed $\varepsilon = \log_{10}(100) = 2$ (i.e. fast, irreversible growth where rate of tile attachment is 100 times larger than the rate of tile detachment) (**Supplementary Figure 1B**, black lines), regardless of the programmed k_{max}, the critical nuclei for ST and HT have nominal widths k^* of 2.3 and 3.1, respectively (**Supplementary Figure 1B**). The barrier for HT is roughly three units higher than that for ST (i.e. 13.9 versus 10.8), thus the relative rate of spurious nucleation is about three orders of magnitude slower (i.e. $10^{-(13.9-10.8)}$) for HT than for ST under these growth conditions.

We were motivated to investigate tiling systems where monomers can have a much larger number of coordination sites to allow far better maintenance of high kinetic barriers under conditions promoting fast, irreversible growth. Therefore we conceived of highly coordinated crisscross slats (CS) that can be programmed to polymerize into ribbons. A CS is a linear array of 2*n* weak-binding sites, each of equal strength and specific for a single conjugate site on one of 2*n* distinct slats of the opposite class (**Supplementary Figure 1A*iii–iv***). The two classes of CS are y-slats and x-slats (top layer and bottom layer, respectively, in **Supplementary Figure 1A**). Specificities are arranged such that, under near-reversible-growth conditions, alternating y-slats and x-slats can add sequentially to the ribbon end only by securing *n* consecutive cross-binding interactions each, as shown by the magenta slat in **Supplementary Figure 1A*iii*** (also see **Supplementary Figure 6**). Slats are able to satisfy their large number of coordination sites only by reaching beyond nearest neighbors (with respect to the center of mass of each slat), such as depicted for $n = 6$ in **Supplementary Figure 1A*iii***.

Near the reversible temperature for growth (i.e. $\varepsilon \rightarrow 0$), the critical nucleus consists of *n* y-slats and *n* x-slats (**Figure 2Ai**, **Supplementary Figure 8**). If we define the width k_{max} such that it equals *n*, then *G(A)* of that critical nucleus is $k_{max}G_{mc}$, the same as for ST and HT. But for fast, irreversible growth, the free energies of CS compact assemblies are preserved far better than those of ST and HT assemblies of comparable width, due to the much smaller $B\varepsilon/n$. For example, ε/n is four times smaller for $CS_{n=8}$ than for ST. Furthermore, since CS bond only to perpendicular neighbors and not to parallel neighbors, whereas ST bond to all nearest neighbors, B_{ST}/B_{CS} approaches two at high *k* (1.75 for $k = 8$) (**Supplementary Table 1**). Collectively, $B\varepsilon/n$ in this case is seven times smaller for CS than for ST. Therefore the free

energy of the CS critical nucleus remains considerable even for relatively large ε; $G(A) = k^{*}G_{mc}$ where k^{*} is $k_{max}/(1 + \varepsilon/G_{mc})$. At $G_{mc} = 7.3$ and $\varepsilon = 2$ (**Supplementary Figure 1C**, orange lines), $CS_{n=6}$ and $CS_{n=8}$ critical nuclei have widths k^{*} of 4.7 and 6.3, respectively, and the spurious nucleation rates are 23.6 (i.e. 34.4 – 10.8) and 35 (i.e. 45.8 – 10.8) orders of magnitude slower than that for ST, respectively (**Supplementary Figure 1C**).

Derivations of the equations:

The following assumptions were made for the comparison: We set a single energy unit to $2.3\ k_B T$, where k_B is the Boltzmann constant and T the temperature. We chose this to yield a $10\times$ per-unit change for variations of the ratio of $growth : shrinkage$ (equation 3), variations in the DNA slat and square-tile concentration, and variations in barrier height. Consequently equations 1, 2, 3, and 4 (adapted from equation 2 and 3 with $\varepsilon = nG_{se} - G_{mc}$, from Evans & Winfree[23]) define the relationship between the dimensionless parameter ε and the $growth : shrinkage$ of the assembly. Equations 5 and 6 are a further adaptation from Evans & Winfree[23] (see section 2 Basic models, sentence above equation 2), defining the free monomer concentration G_{mc}.

$$r_f = \hat{k}10^{-G_{mc}} \quad (1)$$

$$r_{r,n} = \hat{k}10^{-nG_{se}} \quad (2)$$

$$r_f/r_{r,n} = growth : shrinkage = e^{2.3(nG_{se}-G_{mc})} \approx 10^{(nG_{se}-G_{mc})} = 10^{\varepsilon} \quad (3)$$

$$\varepsilon = log_{10}\left(\frac{r_f}{r_{r,n}}\right) \quad (4)$$

$$[c] = u_0 e^{-2.3(G_{mc}+\alpha)} \approx u_0 10^{-(G_{mc}+\alpha)} \quad (5)$$

$$G_{mc} = \alpha - log_{10}\left(\frac{[c]}{u_0}\right) \quad (6)$$

Where $r_f/r_{r,n}$ is the ratio for $growth : shrinkage$, $\hat{k}$ is a reference rate for tile dissociation in s^{-1} (see Evans & Winfree[23], page 4, sentence below equation 2), n is the number of correct bonds formed, G_{se} is

the free energy for a single bond, u_0 is a standard concentration of 1 M, $[c]$ the concentration per DNA slat or square tile monomer, and α a constant unitless parameter accounting for entropic fixation and electrostatic repulsion. For this section we assume $\alpha = log_{10}(20\ M/1\ M) = 1.3$ corresponding to entropy of initiation of -6 cal mol^{-1} K^{-1} (see ref [27]). For the purpose of this example, we set the free monomer concentration $G_{mc} = 1.3 - log_{10}\left(\frac{10^{-6}M}{1M}\right) = 7.3$, i.e. 1 μM each DNA slat or square tile monomer.

The free energy of the assembly

The free energy *G(A)* of an assembly *A* is given by

$$G(A) = (nN - B)(G_{mc}/n) - \boldsymbol{B(\varepsilon/n)} \qquad (7)$$

Square-tile (ST) assembly equations

The number of tiles (*N*) and bonds (*B*) for the square-tile (i.e. *n* = 2) assembly (**Supplementary Figure 2A**) can be described as

$$N = k^2 \qquad (8)$$

$$B = 2k(k - 1) \qquad (9)$$

where *N* and *B* are defined for all nonnegative integers. Inserting equations 8 and 9 into 7 results in the free energy for unbounded assembly

$$G(A) = kG_{mc} - k(k - 1)\varepsilon \qquad (10)$$

where *n* = *2*. The critical nucleus (k^*) is at the maximum free energy (i.e. *dG(A)/dk* = 0) and can therefore be described as

$$k^* = (G_{mc} + \varepsilon)/2\varepsilon \qquad (11)$$

Hexagonal-tile (HT) assembly equations

The number of tiles (*N*) and bonds (*B*) for the hexagonal-tile assembly (**Supplementary Figure 2B**) can be described as

$$N = (3k^2 + 1)/4 \quad (12)$$

$$B = (3/4)(k-1)(3k-1) \quad (13)$$

where N and B are defined for all positive integers. Inserting equations 12 and 13 into 7 results in the free energy for unbounded assembly

$$G(A) = kG_{mc} - (1/4)(3k^2 - 4k + 1)\varepsilon \quad (14)$$

where $n = 3$. We note that $G(A)$ is described for all positive k integers (for $k = 0$, $G(A) = 0$). The critical nucleus (k^*) is at the maximum free energy (i.e. $dG(A)/dk = 0$) and can therefore be described as

$$k^* = 2(G_{mc} + \varepsilon)/3\varepsilon \quad (15)$$

Crisscross (CS) assembly equations

The number of tiles (N) and bonds (B) for crisscross assembly (**Supplementary Figure 2C**) can be described as

$$N = 2k \quad (16)$$

$$B = k^2 \quad (17)$$

where N and B are defined for all nonnegative integers. Inserting equations 16 and 17 into 7 results in the free energy

$$G(A) = (2k - k^2/n)G_{mc} - (k^2/n)\varepsilon \quad (18)$$

where $2n$ is the number of bonds that a crisscross slat can form (**Supplementary Figure 2D**). The critical nucleus (k^*) is at the maximum free energy and can therefore be described as

$$k^* = n/(1 + \tfrac{\varepsilon}{G_{mc}}) \quad (19)$$

The free energy *G(A)* at the critical nucleus *k with respect to *ε* for ST- and HT-nanotube and CS assembly.**

For both ST and HT assembly we define k_{max} as the number of tiles across the diameter of the compact assembly that is the critical nucleus near the reversible temperature (i.e. $\varepsilon \rightarrow 0$). Note that critical nucleus

size is undefined at perfectly reversible conditions (i.e. $\epsilon = 0$); the more relevant concept is critical nucleus size at near-reversible conditions (i.e. $\epsilon \to 0$). For crisscross assembly this equates to $k_{max} = n$ (i.e. the $2n$-length of the slat bounds k_{max}). The free energy $G(A)$ of the critical nucleus and its size k^* as a function of ε are shown **Figure 1C, Supplementary Figure 1C** and **Supplementary Figure 5** respectively for specific values of $k_{max} = 6$ and 8. **Supplementary Figure 3** shows physically how the k_{max} nuclei for ST and HT transition from 2D planar growth into nanotubes.

Equations 11 and 15 describe how the critical nuclei for ST and HT unbounded assembly (i.e. a compact assembly growing in 2D as values of k are increased as per **Supplementary Figure 2**) change with respect to ε. By rearranging equation 11 and 15 and defining k^* as k_{max}, we can compute a transitional ε value ($\varepsilon_{transition}$). For $\varepsilon < \varepsilon_{transition}$ the k^* is fixed to k_{max} with critical nucleus size remaining constant. Once $\varepsilon > \varepsilon_{transition}$ the critical nucleus shrinks as defined by k^* in equation 11 and 14 for ST and HT respectively.

For CS ribbon assembly, k^* with respect to ε is solely described by equation 19. Once the ribbon has passed the nucleation phase and is in the extension phase, the excess cross-binding interactions ($n - k^*$) promote the irreversibility of growth (i.e. $\varepsilon = G_{mc}(n - k^*)/k^*$). Thus any desired barrier height G_{k*} and irreversibility ε can be achieved by scaling n and then adjusting temperature to distribute n between k^* (i.e. size of critical nucleus and therefore height of nucleation barrier) and $n - k^*$ (i.e. growth irreversibility) as desired.

ST-nanotube:

$$G(A)_{k^*ST} = \begin{cases} k_{max}G_{mc} - k_{max}(k_{max}-1)\varepsilon, & \varepsilon < \varepsilon_{transition} \\ \frac{(G_{mc}+\varepsilon)^2}{4\varepsilon}, & \varepsilon > \varepsilon_{transition} \end{cases} \tag{20}$$

with $\varepsilon_{transition} = G_{mc}/(2k_{max} - 1)$

HT-nanotube:

$$G(A)_{k^*HT} = \begin{cases} k_{max}G_{mc} - (1/4)(3k_{max}{}^2 - 4k_{max} + 1)\varepsilon, & \varepsilon < \varepsilon_{transition} \\ \frac{(4G_{mc}{}^2 + 8G_{mc}\varepsilon + \varepsilon^2)}{12\varepsilon}, & \varepsilon > \varepsilon_{transition} \end{cases} \tag{21}$$

with $\varepsilon_{transition} = 2G_{mc}/(3k_{max} - 2)$

CS assembly ($k_{max} = n$)**:**

$$G(A)_{k*\ CS} = nG_{mc}/(1 + \varepsilon/G_{mc}) \tag{22}$$

for $\varepsilon \geq 0$

2.2: Comparison of ST zigzag ribbons versus STN, HTN, and CS ribbons

The possible free energies/rates of nucleation versus the lowest measured experimental nucleation rates for crisscross slats and square-tile zigzag ribbons from ref. [25] is shown with respect to the kTAM model[21–23]. We reported ~0.6 aM/s as our lowest measured nucleation rate, which is ~500-fold lower compared to ~300 aM/s for ref. [25]. We proceed to predict the nucleation rates of crisscross slats from kTAM for the same assumed reversibility ε as ref. [25], though we cannot measure this experimentally using gel methods because the few ribbons in question would be far below the agarose gel detection limit.

Note about the assumption for ε: Gel characterization experiments and TEM observations of crisscross ribbons indicate growth was fast at the conditions where ~0.6 aM/s was observed (i.e. **Figure 3H** with v6.1 slats, 46ºC, 16 mM MgCl2, 1 µM each slat). Let us then assume that $\varepsilon = 1.7$, where growth:shrinkage is 50x, which seems reasonable and more conservative from the $\varepsilon = 2$ 100x growth:shrinkage benchmark in **Figure 1B** and **Supplementary Figure 1B**. Our reasoning is as follows: we note that the measured reversible temperature for slat growth (v6.1, 16 mM MgCl2, 1 µM each slat) was ~56.2ºC, and that near optimal growth was obtained at six degrees cooler (**Figure 3C**), which could correspond roughly to $\varepsilon \approx 1$. Thus we very roughly estimate ε at 46ºC (i.e. ten degrees cooler) to be 1.7. We also assume that growth in the literature precedent in ref. [25] was more reversible, perhaps 2x growth:shrinkage corresponding to $\varepsilon = 0.3$.

For $STZZ_{n=2,\ kmax=3.5}$: (ribbon 6 tiles wide (i.e. $k_{width} = 6$) where $G(A) = 3.5G_{mc}$ at $\varepsilon \rightarrow 0$):

- $\varepsilon = 1.7$ (50x growth:shrinkage), $G_{mc} = 8.0$ (i.e. 0.2 µM each monomer), $G(A) = 17$

- **ε = 0.3 (2x growth:shrinkage), G_{mc} = 8.0 (i.e. 0.2 µM each monomer), *G(A)* = 25.4**
- $\varepsilon \rightarrow 0$ (1x growth:shrinkage), G_{mc} = 8.0 (i.e. 0.2 µM each monomer), *G(A)* = 28.0
- **Nucleation rate observed at ε = 0.3: (300 aM/s or 300*10^{-18} M/s) (ref [25])**
- There is little room to reduce nucleation rates by diminishing ε because ε = 0.3. Further stringency would make growth exceedingly slow.

For $STZZ_{n=2,\ kmax=6}$: (ribbon 11 tiles wide (i.e. k_{width} = 11) where *G(A)* = 6G_{mc} at $\varepsilon \rightarrow 0$):

- ε = 1.7 (50x growth:shrinkage), G_{mc} = 8.0 (i.e. 0.2 µM each monomer), *G(A)* = 24.2
- ε = 0.3 (2x growth:shrinkage), G_{mc} = 8.0 (i.e. 0.2 µM each monomer), *G(A)* = 39.0
- $\varepsilon \rightarrow 0$ (1x growth:shrinkage), G_{mc} = 8.0 (i.e. 0.2 µM each monomer), *G(A)* = 48

For $CS_{n=6}$:

- **ε = 1.7 (50x growth:shrinkage), G_{mc} = 7.3 (i.e. 1 µM each monomer), *G(A)* = 35.5**
- ε = 0.3 (2x growth:shrinkage), G_{mc} = 7.3 (i.e. 1 µM each monomer), *G(A)* = 42.1
- $\varepsilon \rightarrow 0$ (1x growth:shrinkage), G_{mc} = 7.3 (i.e. 1 µM each monomer), *G(A)* = 43.8
- ε = 1.7 (50x growth:shrinkage), G_{mc} = 8.0 (i.e. 0.2 µM each monomer), *G(A)* = 39.6
- **ε = 0.3 (2x growth:shrinkage), G_{mc} = 8.0 (i.e. 0.2 µM each monomer), *G(A)* = 46.3**
- $\varepsilon \rightarrow 0$ (1x growth:shrinkage), G_{mc} = 8.0 (i.e. 0.2 µM each monomer), *G(A)* = 48
- Nucleation rate observed at ε = 1.7: (~0.6 aM/s or 0.6*10^{-18} M/s)
- Changing from (ε = 1.7, G_{mc} = 7.3) → (ε = 0.3, G_{mc} = 8.0) changes the nucleation energy from 35.5 → 46.3
- Difference in the barrier by changing ε from (1.7, G_{mc} = 7.3) → (0.3, G_{mc} = 8.0) is 46.3 – 35.5 = 10.8 orders-of-magnitude
- **Hence, the nucleation rate is predicted to lessen from ~0.6*10^{-18} M/s → ~0.6*$10^{-(18+10.8)}$ M/s,** if one were to grow the slats at more stringent conditions with lower monomer concentration as done by ref. [25]. We further highlight that the experimental measurement in our work was done using high 16 mM $MgCl_2$ and v6.1 slats—the $MgCl_2$ could be lessened and the slat version changed to v6.2 to further decrease spurious nucleation, as suggested in **Figure 3I**, **Supplementary Figure 38**, and **Supplementary Figure 39**.

The values tabulated above for square tiles assume zigzag ribbons, as per the monomer design used by ref. [25]. The energetic differences resulting from the exact square tile architecture, be it square tile nanotubes or square tile zigzag ribbons, do not alter our conclusions about the relative robustness of

crisscross (**Supplementary Figure 6**). In particular, we note that at large ε (e.g. 1.7) and low G_{mc} (e.g. 7.3 or 8.0) the nucleation energy of square tile nanotubes and zig-zag ribbons will be identically small in comparison to crisscross slats.

Square-tile zigzag (STZZ) assembly equations

Equation 23 (see Evans & Winfree[23], page 12, left column) describes the free energy $G(A)$ at the critical-nucleus k^* versus ε, for a zigzag ribbon of width $k_{width.}$

$$G(A)_{k^*,STZZ} = (k_{width} + 1)(G_{mc} + \varepsilon)/2 - (2k_{width} - 2)\varepsilon \quad (23)$$

For STZZ, we normalized $G(A)_{k^*,STZZ}$ as $k_{max}G_{mc}$ at $\varepsilon \rightarrow 0$, in order to better compare STZZ to STN, HTN, and CS ribbons. Therefore, we redefined $k_{max} = 0.5(k_{width} + 1)$ or $k_{width} = 2k_{max} - 1$ (**Supplementary Figure 6**, equation 24–25).

STZZ , with k_{width}: $$G(A)_{k^*,STZZ} = (k_{width} + 1)(G_{mc} + \varepsilon)/2 - (2k_{width} - 2)\varepsilon \quad (24)$$

STZZ , with $k_{width} = 2k_{max} - 1$: $$G(A)_{k^*,STZZ} = k_{max}G_{mc} + \varepsilon(4 - 3k_{max}) \quad (25)$$

2.3: Toy model for building intuition on why designs with larger *n* have smaller *Bε/n*

The goal of this section is to provide a toy model as an explanatory framework that suggests why, comparing larger-n to smaller-n monomer designs for compact assemblies of equivalent width k_{max} (i.e. $G(A) = k_{max}G_{mc}$ for all designs at $\varepsilon \rightarrow 0$), the reversible-growth credit BG_{mc}/n required to hold monomers together in the metastable assembly is expected to be smaller for larger-n designs, and therefore the excess credit $B\varepsilon/n$ providing extra stability to the compact assembly is expected to be smaller as well.

From inspecting compact assemblies of width k for $ST_{n=2}$ and $CS_{n=k}$ in **Supplementary Figure 2***,* we see that the total number of coordination sites $2nN$ is $4k^2$, the number of those that are unbonded $2(nN - B)$ is $2nk$, and the number of those that are bonded $2B$ is $4k^2 - 2nk$. Therefore the reversible-growth bonding credit BG_{mc}/n is $(2k^2/n - k)G_{mc}$, and the excess bonding credit $B\varepsilon/n$ is $(2k^2/n - k)\varepsilon$. The number of monomers N is $N = B/n + (N - B/n) = B/n + k$ (**Supplementary Figure 9**) or $2k^2/n$.

But since $G(A) = k_{max}G_{mc}$ at $\varepsilon \rightarrow 0$ for all designs according to our definition of k_{max}, then $2(nN - B)$ must indeed be $2nk$ for all designs at $k = k_{max}$. Thus it appears for $k = k_{max}$ we only need to make one

assumption, that the number of coordination sites is $4k^2$, in order to fix B/n at $2k^2/n - k$, such that n is the only degree of freedom comparing ST and $CS_{n=k}$. Therefore $(B/n)_{CS}/(B/n)_{ST} = ((2k^2/k) - k)/((2k^2/2) - k) = 1/(k - 1)$. For example, for $n_{CS} = k = 8$, then $(B/n)_{CS}/(B/n)_{ST} = 1/7$, just as we found in the main text.

To rationalize this, we can create a toy model to imagine a hypothetical transformation from $ST_{n=2}$ to $CS_{n=k}$. We conceive of a compact assembly as a collection of $2nN = 4k^2$ discrete nodes, where initial capture of each incurs a debit G_{nc}. Nodes are sorted into monomers (i.e. there are N monomers each with $2n$ distinct nodes) and are connected to each other by a combination of intra-monomer linkages (the new concept) and inter-monomer bonds (same as before, as described in the main text and other supplemental text) (**Supplementary Figure 10A−C**). Each node bears a single coordination site, which either can participate in an inter-monomer bond or else be unbonded (same as before). In our simple toy model, the $2n$ nodes of a monomer are connected in a linear array with $2n - 1$ intra-monomer linkages. We set the credit of each intra-monomer linkage to a magnitude that exactly offsets the debit for initial capture of a node, i.e. $-G_{nc}$ (which we set as equal to $-G_{mc}$ since the concentration of a unique node is the same as the concentration of the unique monomer in which it is grouped). Therefore the sum of debits for initial capture of the $2n$ nodes of a monomer and credits from $2n - 1$ intra-monomer linkages is $2nG_{nc} - (2n - 1)G_{nc} = G_{nc}$, or equal to the debit for initial capture of a monomer G_{mc}, just as before. The potential free energy of each inter-monomer bond still is $(G_{nc} + \varepsilon)/n$, and at least half the coordination sites in each monomer must be bonded for stable capture, all as before. Then the free energy of compact assembly A is the sum of debits from initial capture of nodes, credits from inter-monomer bonds, and credits from intra-monomer linkages.

$$G(A) = 2nNG_{nc} - (B/n)(G_{nc} + \varepsilon) - N(2n - 1)G_{nc} = (N - B/n)G_{nc} - B\varepsilon/n$$

This reduces to the sum of debits from unbonded coordination sites and excess credits from bonded coordination sites, just as before, since our hypothetical breaking of monomers into nodes and then linking them back together to reform monomers has a net-zero impact on assembly free energy.

What is new is that we now can assess "reversible-growth cohesion credits" as the sum of those from inter-monomer bonds and those from intra-monomer linkages, i.e. $-(B/n + N(2n - 1))G_{nc}$. We can see that these cohesion credits are conserved (i.e. $B/n + N(2n - 1) = 4k^2 - k$ or the number of nodes minus the number of outstanding G_{nc} debits in the assembly) in the transformation from ST to $CS_{n=k}$, where every additional intra-monomer linkage (worth one G_{nc}) is offset by a net decrease of one unit in B/n (worth one G_{nc}). Therefore the transformation trades cohesion credits from renormalized inter-monomer bonding with

equivalent cohesion credits from intra-monomer linking (i.e. $\Delta(B/n) = -\Delta(N(2n-1))$). Or more qualitatively, critical nuclei of equivalent width k_{max} require decreasing amounts of inter-monomer bonding energy to hold together as the monomer coordination number rises, since intramonomer linkages are doing an increasing amount of that "work".

Here are two examples comparing ST to HT to $CS_{n=k}$, for $k = 7$ and $k = 101$, showing cohesion credits in units of $-G_{nc}$:

***k* = 7**
ST: $N = 49$, $B = 84$; **2*nN*** = 2*2*49 = **196**; cohesion credits = $B/n + N(2n-1)$ = 84/2 + 49*3 = 189
HT: $N = 37$, $B = 90$; **2*nN*** = 2*3*37 = **222**; cohesion credits = $B/n + N(2n-1)$ = 90/3 + 37*5 = 215
$CS_{n=k}$: $N = 14$, $B = 49$; **2*nN*** = 2*7*14 = **196**; cohesion credits = $B/n + N(2n-1)$ = 49/7 + 14*13 = 189

***k* = 101**
ST: $N = 10201$, $B = 20200$; **2*nN*** = 2*2*10201 = **40804**; cohesion credits = $B/n + N(2n-1)$ = 20200/2 + 10201*3 = 40703
HT: N = 7651, B = 22650; **2*nN*** = 2*3*7651 = **45906**; cohesion credits = $B/n + N(2n-1)$ = 22650/3 + 7651*5 = 45805
$CS_{n=k}$: N = 202, B = 10201; **2*nN*** = 2*101*202 = **40804**; cohesion credits = $B/n + N(2n-1)$ = 10201/101 + 202*201 = 40703

As a more general treatment, reversible-growth cohesion credits versus k is plotted for ST, HT, and CS in **Supplementary Figure 10D**. We can see that the number of nodes approaches $4.5k^2$ for HT at large k, compared to $4k^2$ for ST and $CS_{n=k}$ (i.e. 12.5% greater number of nodes for HT). Nonetheless, the cohesion credits per node $(B/n + N(2n-1))/2nN$ converges to one for all three designs as k increases, which makes sense since as k rises, the ratio of unbonded coordination sites to bonded coordination sites approaches zero. Therefore additional considerations, such as geometry, would need to be added to our simple toy model for accurate accounting of transformations to other designs besides *ST* and $CS_{n=k}$ (e.g. number of nodes are not necessarily conserved during transformations due to differences in geometry).

2.4: In our simplistic kTAM model, how does lowering the temperature affect the barrier height?

- Lower temperature increases binding energy, i.e. increases ε
- Lower monomer concentration increases entropic penalty for capture, i.e. increases G_{mc}
- Higher salt concentration reduces electrostatic repulsion and therefore increases net binding energy, i.e. increases ε.

However, we believe other effects come into play as well. For example, slats may bind each other non-specifically, leading to kinetic trapping that becomes more prominent at lower temperatures. Kinetic trapping usually is associated with suppression of spurious nucleation. On the other hand, it is conceivable

that some off-target ensembles could be formed that can quickly rearrange into critical nuclei, therefore the rate of spurious nucleation could be increased.

For the reasons outlined above, it is simple to explain the direction of the trends qualitatively but difficult to make quantitative predictions as binding energies increase (e.g. lower temperatures). We emphasize that the most important conclusion from a design perspective is that each increment of n leads to a several-order-of magnitude decrease in the rate of spurious nucleation at micromolar slat concentrations even under highly irreversible growth conditions, and that the exact magnitude of this decrease becomes less important given the ability to scale n. By analogy, the power of DNA origami is that the designed structure is far more favored over unanticipated off-target structures, regardless of our inability to predict quantitatively what the energetic difference is.

Supplementary Discussion 3

Ribbon assembly characteristics by experimental observation and modelling: This section describes and characterises the model used in **Figure 3F–G**. To validate the mechanism by which slats assemble into crisscross ribbons, we built a stochastic model to simulate their assembly, and developed an analytical solution of this model. We tested the model by fitting to length distribution data obtained by TEM and agarose gel densitometry collected at various reaction conditions. Conditions include **Supplementary Figure 29** ("optimal" 50ºC, v6.1 1 µM each slat, 16 mM $MgCl_2$), **Supplementary Figure 30** ("suboptimal" nucleation-prone 40ºC, v6.1 1 µM each slat, 20 mM $MgCl_2$), **Supplementary Figure 31** ("optimal" 50ºC, v6.2 1 µM each slat, 16 mM $MgCl_2$), and **Supplementary Figure 32** ("optimal" 50ºC, v6.2 1.5 µM each slat, 14 mM $MgCl_2$).

We postulate that there are three distinct phases of assembly: initiation, growth, and termination. Initiation can occur from either a DNA seed, or from a spuriously formed nucleus. Growth subsequently proceeds at a relatively constant rate until ribbon growth terminates due to accumulation of defects or impurities. The growth may occasionally stall and subsequently recover, for example due to an incorrect slat temporarily binding to the terminus of a ribbon.

From these mechanistic assumptions, we reproduce experimentally measured ribbon length distributions over time in a stochastic model considering only nucleation rate, growth rate, stalling probability, and termination probability. Each available seed has a fixed probability of nucleating a ribbon at each timepoint. Spurious nucleation occurs at a fixed rate throughout the assembly time. Once initiated, a

ribbon grows at a fixed rate until its growth is terminated with a fixed probability of termination at each timepoint. At each timepoint in ribbon growth, the ribbon has a fixed probability of stalling (stopping growth), or if stalled, of restarting growth.

We assume that effects from changes in concentration of the slats from monomer depletion during growth are negligible because most experiments had large excesses of slats (1–1.5 μM for each slat). Additionally, this model simplifies growth as a continuous coarse-grained increase of ribbon length, as opposed to discrete incorporation of single slats.

**Parameter**	**Notation**	**Fitting**
Growth rate	l_{growth}	TEM data
Probability of termination	p_{term}	TEM data
Probability of stalling and recovery	p_{stall}	TEM data
Probability of seed nucleation	p_{seed}	Gel data
Spurious nucleation rate	r_{nuc}	Gel data
Number of seeds	N_{seed}	Constant/gel data
Total assembly duration	T_{final}	Constant
Timepoint	t	–
Length of ribbon k	l_k	–
State of ribbon k (seed/growing/terminated)	s_k	–

List of parameters used in stochastic model and analytical solution, and the data used to fit each parameter (if applicable). Probabilities of termination, stalling and seed nucleation were used as rates in the analytical solution, and hence referred to as λ_{term}, λ_{stall} and λ_{seed}

respectively in **Supplementary Discussion 3.4**. The number of seeds is taken as constant for the stochastic simulations but is fitted in the gel data analysis.

Stochastic model algorithm: Begin with a population of N_{seed} seeds (i.e. uninitiated ribbons) by initialising the ribbon state $s_k \leftarrow$ "*growing*" and length $l_k \leftarrow 0$ for ribbons $k \in \{1, \ldots, N_{seed}\}$.

For every timepoint $t \in \{1, \ldots, T_{final}\}$:

1. Generate r_{nuc} new ribbons by initialising each of these ribbons with state $s_k \leftarrow$ "*growing*" and length $l_k \leftarrow 0$ for ribbons $k \in \{1, \ldots, r_{nuc}\}$.
2. For every stalled ribbon k such that $s_k ==$ "*stalled*", set $s_k \leftarrow$ "*growing*" with a probability of p_{stall}.
3. For every growing ribbon k such that $s_k ==$ "*growing*", add a constant length l_{growth} to l_k, i.e. $l_k \leftarrow l_k + l_{growth}$.
4. For every ribbon k such that $s_k ==$ "*growing*" or $s_k ==$ "*stalled*", set $s_k \leftarrow$ "*terminated*" with a probability of p_{term}.
5. For every growing ribbon k such that $s_k ==$ "*growing*", set $s_k \leftarrow$ "*stalled*" with a probability of p_{stall}.

The output of the algorithm is the sample of lengths l_k for all the growing and terminated ribbons such that $s_k ==$ "*growing*", $s_k ==$ "*stalled*", or $s_k ==$ "*terminated*".

When considering only seeded growth (for example in the TEM analyses in **Supplementary Figures 29–32**), step 1 was omitted, effectively meaning $r_{nuc} = 0$. Similarly, when considering spurious assembly only, N_{seed} was set to zero (i.e. run the algorithm without an initial population of ribbons).

3.1: Kinetics by TEM observation versus model fit

The lengths of ribbons nucleated from a seed were measured using TEM for different assembly conditions and assembly times. For each timepoint we measured approximately 150 ribbons (for actual N see figure legends of **Supplementary Figures 29–32**) , and so the number of seeds used for the simulations was also 150. Only ribbons that had a visible seed attached to one end were measured. Therefore, in the

stochastic simulations we can ignore the contributions of spurious nucleation. We also assume that stalling and recovery occur by similar mechanisms (for example the reversible binding of incorrect slats), and therefore we take the probability of stalling and of recovery to be equal for simplicity. To justify this assumption, we tried fitting these two parameters separately, which resulted in very similar parameter values for stalling and recovery – having them as two separate variables also did not significantly improve the fit.

The p_{stall}, p_{term}, and l_{growth} parameters in the stochastic model were fit to the TEM data using the Kolmogorov-Smirnov (KS) statistic to compare the cumulative distribution functions of ribbon lengths of the model and the data. For every experimental condition, the mean of the KS statistics for each assembly time was minimised with the Nelder-Mead method. Using a timestep of one minute, this resulted in a stalling and recovery probability of ~0.03-0.1 per timestep, a termination probability of ~0.00005-0.005 per timestep, and a growth rate of ~4-30 nm/minute (**Supplementary Figures 29−32**). Fitted parameters for each simulation are shown in the respective figure legends.

In particular, optimal growth conditions yielded growth rates of ~30 nm/minute and termination and stall probabilities on the order of 0.05 per minute. Suboptimal growth conditions (i.e. lower temperature, **Supplementary Figure 30**) resulted in slower assembly kinetics, with significantly lower growth rates (~4 nm/minute) and lower termination probabilities (~0.00005 per minute), giving rise to seemingly linear growth over the duration of the assembly. We speculate that at lower temperatures, that since fewer bonds are needed per slat addition, therefore a larger number of truncation defects are required for termination. We further note that effects such as kinetic trapping at lower hybridization free energies are not explicitly captured in this model. While experimental limitations currently preclude the use of the model beyond the validation of our conceptual understanding of crisscross assembly, we expect that in the future, by fitting the model at a greater number of experimental conditions, we may be able to utilize predictions from the model to help optimize experimental conditions to attain desirable growth properties, i.e. maximize growth and recovery rates while minimizing termination and stall rates.

Polydispersity index was calculated as $\overline{L}_W/\overline{L}_N$, where $\overline{L}_W$ is the weight average length $\overline{L}_W = \frac{\sum_{i=1}^{\infty} N_i L_i^2}{\sum_{i=1}^{\infty} N_i L_i}$ and $\overline{L}_N$ is the number average length $\overline{L}_N = \frac{\sum_{i=1}^{\infty} N_i L_i}{\sum_{i=1}^{\infty} N_i}$ (where N_i is the number of ribbons of length L_i), and was found to be in the range of 1.07−1.53 for the experimental measurements (see legends of

Supplementary Figures 29−32 for exact values). While previous work in crystallization-driven living self-assembly has produced significantly lower polydispersity indices (e.g. less than 1.03 for cylindrical micelle assembly in ref [28]), crisscross ribbons are less disperse than previously reported systems in DNA self-assembly. For example, we analysed the results from ref [15] Figure 3C to find that living supramolecular polymerization of DNA by HCR over a 2 hour incubation yields dispersities of ~1.4 (c.f. ~1.2 for crisscross). Analysing the results from ref [29] Figure 3C, we find that DNA nanotubes have a dispersity of ~1.8 after a 16-hour anneal − in contrast crisscross ribbon dispersity is typically < 1.5 for similar incubations (polydispersion in these cases is likely due to the prevalence of growth termination for very long growth series). Furthermore, we expect that a reduction in attainable dispersity can be achieved by further exploration of slat sequence design (**Supplementary Table 2**) and optimisation of experimental parameters to reduce stalling and termination probabilities.

3.2: Observation of spurious ribbons on agarose gels versus model fit

We were unclear whether the spurious assembly observed in experiments using certain reaction conditions was the result of nuclei that had formed during some early stage when the reagents were mixed, or if they were forming throughout the growth period at some constant spurious nucleation rate (i.e. r_{nuc}). To elucidate this process, we compared spurious and seed-initiated growth of v6.1 on agarose gels versus time using conditions that favored spurious ribbon formation (40ºC, 20 mM $MgCl_2$, 1 µM each slat) or growth optimal conditions that disfavored it (52ºC, 20 mM $MgCl_2$, 1 µM each slat). We used the stochastic model and analytical solution (which were developed with the hypothesis that spurious nucleation occurs at constant rate r_{nuc}) to see if observed gel densitometry data for spurious and seeded-initiated assembly could be recreated *in silico*.

We assume measured intensity of a gel band at a given position on the gel is proportional to the number of ribbons where the relative SYBR-Gold gel stain fluorescence per ribbon scales linearly with its length (i.e. accounting for the amount of stain bound to the net amount of DNA in each ribbon). To apply the model to recreate the gel densitometry profiles, we scaled the ribbon counts by their corresponding length. The total intensity in both the experiment and model is the integration of the intensity vs gel position (i.e. ribbon length). For experimental data, gel intensity profiles were extracted using ImageJ and total intensity was calculated as the area under the curve.

We estimated p_{stall}, p_{term}, and l_{growth} parameters from the TEM data (**Supplementary Figure 30**). These parameters were fixed in the analytical solution of the model (see **Supplementary Discussion 3.3**

for further motivation and **Supplementary Discussion 3.4** for derivation), which was used to fit the r_{nuc} parameter to the total intensities of assembly when no seed was present. The fitted r_{nuc} parameter was subsequently used to fit the number of seeds N_{seed} to the total intensities of assembly in seeded conditions based on the analytical solution.

To recreate the "seed" peak in the gel intensity profiles for seeded assembly, we needed to assign leftover seeds a certain size (relative to ribbon length). Thus, we took seed size to be 85.2 nm, and added Gaussian noise with a mean of 0 nm and standard deviation of 50 nm to prevent singularities in plotting. While it is not possible to directly quantify the effective size of the DNA seed relative to ribbon length in terms of how it would run on a gel, we estimated its effective size by considering that the seed contributes 4032 nucleotides. The average length of a single slat is 71 nucleotides, and we can approximate that each slat contributes around 1.5 nm to the length of the ribbons. Thus, the effective seed size can be estimated to be 85.2 nm, though in practice simulations are robust to variations in the seed size used since it simply determines the starting position of the simulated gel intensity profile.

Using gel data for experimental conditions permissive to spurious nucleation (40°C, 20 mM $MgCl_2$, v6.1), we fit the analytical equation for total gel intensity to the mean areas of the gel intensity profiles (**Supplementary Figure 33A–B** for gel, **Supplementary Figure 33C** for fit). The ratio between the number of seeds present and the spurious nucleation rate (per minute) was estimated to be 1148. This would mean that for a concentration of 2 nM seed at these conditions, we would expect a spurious nucleation rate of 1.7 pM per minute ($2.8 * 10^4$ aM/s). Note that we are unable to make similar estimates for other experimental conditions as spurious nucleation is too low to be detectable by gel analysis.

The stochastic model was executed with $N_{seed} = 22961$ and $r_{nuc} = 20$ per minute (corresponding to the ratio calculated through the analytical solution fit) and the parameters estimated in **Supplementary Figure 30**. A continuous distribution of ribbon lengths was generated using Epanechnikov kernel density estimation based on the model outputs. Given that gels separate linear DNA logarithmically based on length, we assumed that logarithmic separation based on ribbon length would be a reasonable qualitative approximation of how ribbons may run on the gel. Thus, the products of the relative counts and lengths were plotted on a logarithmic scale to recreate the expected gel intensity profiles for different assembly durations, giving good qualitative agreement between the model and the data (**Supplementary Figure 33D**).

3.3: Analytical solution of ratiometric gel analysis

In order to be able to calculate the expected ratio of gel intensities (proportional to the total mass of ribbons present) resulting from spurious nucleation of a 100-hour incubation relative to a 1-hour incubation (the experiment in **Figure 3H, Supplementary Figure 36**), we derived an analytical solution to our probabilistic model (see **Supplementary Discussion 3.4** for derivation). This analytical solution was found to be in good agreement with the stochastic model simulations (**Supplementary Figure 34A**).

Under this model, the expected ratio solely depends on stalling and termination probability (**Supplementary Figure 34B−C**). Based on our estimates of these probabilities from fitting the TEM data, through the analytical solution we can expect an approximately 500- to 8000-fold increase in mass (corresponding to total gel intensity) due to spurious nucleation for a 100-hour incubation relative to a 1-hour incubation. When comparing the increase in mass after 100 hours to that after 1 hour and ignoring stalling, with zero termination we would expect a 10,000-fold increase in mass (quadratic growth as a result of a linear increase in the number of ribbons and linear growth), while with 100% termination at each step we would expect a 100-fold increase in mass (linear increase in number of ribbons).

The effect of stalling is minor in this case when modelling unseeded growth, though it is most pronounced for intermediate stalling probabilities. Intuitively, there would be a substantial proportion of ribbons that would not have stalled after 1 hour, while after 100 hours they would have likely reached a steady state of about 50% of non-terminated ribbons being stalled. At high stalling probabilities, the majority of ribbons at both 100 hours and 1 hour are likely to have reached the steady state of growing 50% of the time, and thus the ratio between 100 hours and 1 hour increases again. This is manifested as a small dip in **Supplementary Figure 34C**.

3.4: Analytical solution derivation

In order to enable the ratiometric analysis discussed in **Supplementary Discussion 3.3**, we derived an analytical solution to our stochastic model. Every growing ribbon is assumed to have a fixed probability of terminating at each timestep. Thus, for ribbons nucleated at $t = 0$, the proportion of ribbons terminated at each timestep t is given by the probability density function of an exponential distribution with rate parameter λ_{term}. If we ignore stalling, given a fixed growth rate l_{growth}, the length of a ribbon that has grown for T timesteps is $l_{growth}\,T$. With a fixed probability of stalling and recovery from a stall λ_{stall}, the proportion of ribbons growing at timestep t is given by $0.5 + 0.5\,e^{-2\,\lambda_{stall}\,t}$. Hence the total length of a

ribbon growing for T timesteps is the definite integral of this expression from $t = 0$ to $t = T$, which gives a total length of $\left(\frac{T}{2} - \frac{e^{-2\lambda_{stall} T} - 1}{4\lambda_{stall}}\right) l_{growth}$. Thus, for N_{seed} growing ribbons, the total length of ribbons whose growth has terminated by T_{final} is given by:

$$\sum_{\substack{k \in \{1 \ldots N_{\text{seed}}\} \\ s_k = \text{"terminated"}}} l_k = \sum_{t=0}^{T_{\text{final}}} \left(\frac{t}{2} - \frac{e^{-2\lambda_{\text{stall}} t} - 1}{4\lambda_{\text{stall}}}\right) l_{\text{growth}} N_{\text{seed}} \lambda_{\text{term}} e^{-\lambda_{\text{term}} t} \quad (1)$$

And the total length of ribbons still growing at T_{final} is given by:

$$\sum_{\substack{k \in \{1 \ldots N_{\text{seed}}\} \\ s_k = \text{"growing"}}} l_k = \left(\frac{T_{\text{final}}}{2} - \frac{e^{-2\lambda_{\text{stall}} T_{\text{final}}} - 1}{4\lambda_{\text{stall}}}\right) l_{\text{growth}} N_{\text{seed}} e^{-\lambda_{\text{term}} T_{\text{final}}} \quad (2)$$

Equations (1) and (2) can be summed to give the total lengths for seeded assembly.

By comparison, for spurious assembly, the number of ribbons formed at each timestep corresponds to the fixed rate r_{nuc}. Thus, at the end of the incubation, there are $T_{final} r_{nuc}$ ribbons present. Therefore, equation (1) becomes:

$$\sum_{\substack{k \in \{1 \ldots T_{\text{final}} r_{\text{nuc}}\} \\ s_k = \text{"terminated"}}} l_k = \sum_{t_{\text{nuc}}=0}^{T_{\text{final}}} \sum_{t=0}^{T_{\text{final}} - t_{\text{nuc}}} \left(\frac{t}{2} - \frac{e^{-2\lambda_{\text{stall}} t} - 1}{4\lambda_{\text{stall}}}\right) l_{\text{growth}} r_{\text{nuc}} \lambda_{\text{term}} e^{-\lambda_{\text{term}} t} \quad (3)$$

Similarly, equation (2) becomes:

$$\sum_{\substack{k \in \{1 \ldots T_{\text{final}} r_{\text{nuc}}\} \\ s_k = \text{"growing"}}} l_k = \sum_{t_{\text{nuc}}=0}^{T_{\text{final}}} \left(\frac{(T_{\text{final}} - t_{\text{nuc}})}{2} - \frac{e^{-2\lambda_{\text{stall}} (T_{\text{final}} - t_{\text{nuc}})} - 1}{4\lambda_{\text{stall}}}\right) l_{\text{growth}} r_{\text{nuc}} e^{-\lambda_{\text{term}} (T_{\text{final}} - t_{\text{nuc}})} \quad (4)$$

Equations (3) and (4) can be summed to give the total lengths for spurious assembly. Correspondingly, seeded and spurious assembly can be summed to give total assembly in the presence of a seed (for example in the "Seeded assembly" conditions in **Supplementary Figure 33**).

3.5: Consideration of delayed seed initiation

In the simulations described in previous sections, we assumed that all the seeds nucleate ribbons at the beginning of the incubation. However, for seeded assembly, we can still see a prominent band corresponding to uninitiated seed in certain experimental conditions (for example, the 2-hour gel data in **Supplementary Figure 33**). Thus, we can conclude that the seeds do not all initiate ribbons at $t = 0$. While the approximation that seed initiation is instantaneous is sufficient to get good agreement with both TEM and gel data, in some instances we might be interested in describing the behaviour of the seeds themselves. In this case, we can assume that seed nucleation occurs at a constant rate, and therefore the fraction of ribbons nucleated at timestep t follows an exponential distribution with rate λ_{seed}, i.e. there will be $N_{seed}\, \lambda_{seed} e^{-\lambda_{seed}\, t_{nuc}}$ ribbons that have nucleated at the timestep t_{nuc}. Thus, equation (1) becomes:

$$\sum_{\substack{k\in\{1\ldots N_{\text{seed}}\}\\ s_k=\text{"terminated"}}} l_k = \sum_{t_{\text{nuc}}=0}^{T_{\text{final}}} \sum_{t=0}^{T_{\text{final}}-t_{\text{nuc}}} \left(\frac{t}{2} - \frac{e^{-2\,\lambda_{\text{stall}}\,t}-1}{4\,\lambda_{\text{stall}}}\right) l_{\text{growth}}\, N_{\text{seed}}\lambda_{\text{seed}}\, e^{-\lambda_{\text{seed}}\,t_{\text{nuc}}}\lambda_{\text{term}}\, e^{-\lambda_{\text{term}}\,t} \quad (5)$$

Similarly, equation (2) becomes:

$$\sum_{\substack{k\in\{1\ldots N_{\text{seed}}\}\\ s_k=\text{"growing"}}} l_k = \sum_{t_{\text{nuc}}=0}^{T_{\text{final}}} \left(\frac{(T_{\text{final}}-t_{\text{nuc}})}{2} - \frac{e^{-2\,\lambda_{\text{stall}}\,(T_{\text{final}}-t_{\text{nuc}})}-1}{4\,\lambda_{\text{stall}}}\right) l_{\text{growth}}\, N_{\text{seed}}\, \lambda_{\text{seed}} e^{-\lambda_{\text{seed}}\,t_{\text{nuc}}}\, e^{-\lambda_{\text{term}}\,(T_{\text{final}}-t_{\text{nuc}})} \quad (6)$$

In the stochastic simulation of the model, this can be implemented by beginning with a population of N_{seed} seeds (i.e. uninitiated ribbons) by initialising the ribbon state $s_k \leftarrow$ "*seed*" and length $l_k \leftarrow 0$ for ribbons $k \in \{1, \ldots, N_{seed}\}$. Subsequently, at every timepoint $t \in \{1, \ldots, T_{final}\}$, for every uninitiated ribbon k such that $s_k ==$ "*seed*", set $s_k \leftarrow$ "*growing*" with a probability of p_{seed}.

From the gel data in **Supplementary Figure 33**, we can estimate that p_{seed} for these specific experimental conditions is around 0.003 per minute (**Supplementary Figure 35A**). Incorporation of seed initiation into the model is capable of reproducing a large portion of the variance in the data even when stalling is excluded from the model (**Supplementary Figure 35B−D**). However, since seed initiation is not capable of reproducing the true shape and symmetry of the length distributions, preference was given for stalling as the main source of variance in ribbon length, and for simplicity seed initiation was assumed to be instantaneous in all of the analyses in this paper.

Supplementary Figures 1–46

Supplementary Figures 1–10: Principles of crisscross-slat versus square-tile and hexagonal-tile nucleation

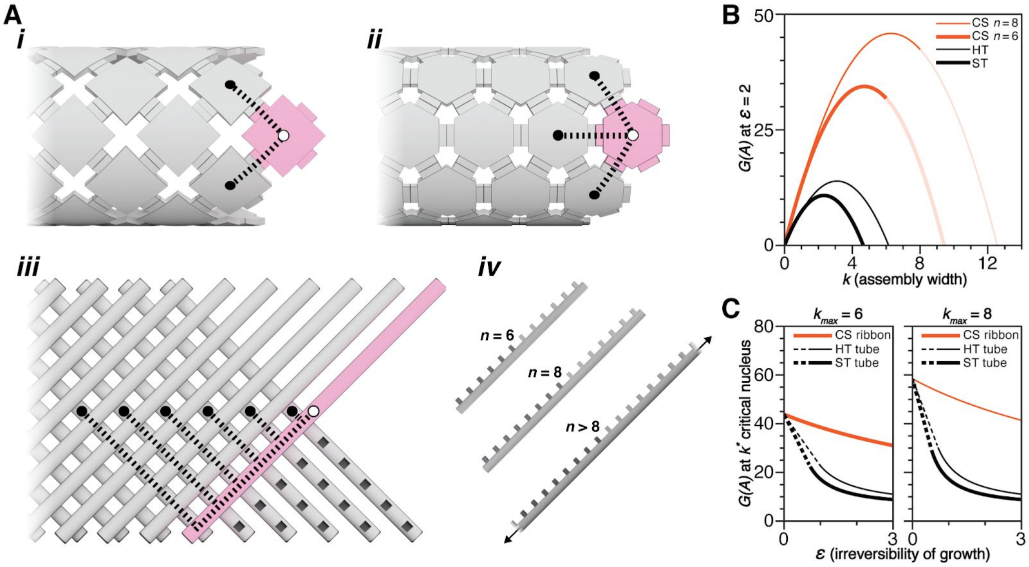

Supplementary Figure 1. Variation of Figure 1 to fully explain Supplementary Discussion 2, with differences in panels B–C. High monomer coordination number (2*n*) preserves large barriers to spurious nucleation under increasingly irreversible growth conditions, with a theoretical comparison of square-tile (ST) nanotubes, hexagonal-tile (HT) nanotubes, and crisscross-slat (CS) ribbons of comparable width. **A**, Under near-reversible-growth conditions, incoming ST (***i***) and HT (***ii***) bind two or three nearest neighbors respectively to elongate tubes, whereas incoming $CS_{n=6}$ (***iii)*** bind to six slats to elongate ribbons. ***iv*,** Slats may be arbitrarily extended to any positive integer *n* to increase coordination number. **B**, Free energy *G(A)* of an assembly *k* is greater for CS at irreversible $\varepsilon = 2$ conditions (i.e. $10^{\varepsilon} = 100{:}1$ growth:shrinkage, "high" 1 µM monomer). The energy maxima for the critical nuclei are the same as Figure 1B, except they are plotted against the assembly width parameter *k* instead of the number of *N* monomers. For CS, $k_{max} = n$; *G(A)* where $k > n$ is indicated with faded lines because they are not physically tenable. Addition of a slat once k_{max} is reached increments the number of *B* bonds by *n* to linearly change *G(A)*, as in Figure 1B. **C**, Larger free energies *G(A)* for k^* critical nuclei of slats are sustained for irreversible fast-growth large ε conditions compared to the tiles, with 1 µM monomer concentration. Dotted versus solid lines for ST and HT indicate where the critical nucleus transitions from $k^* = k_{max}$ to $k^* < k_{max}$.

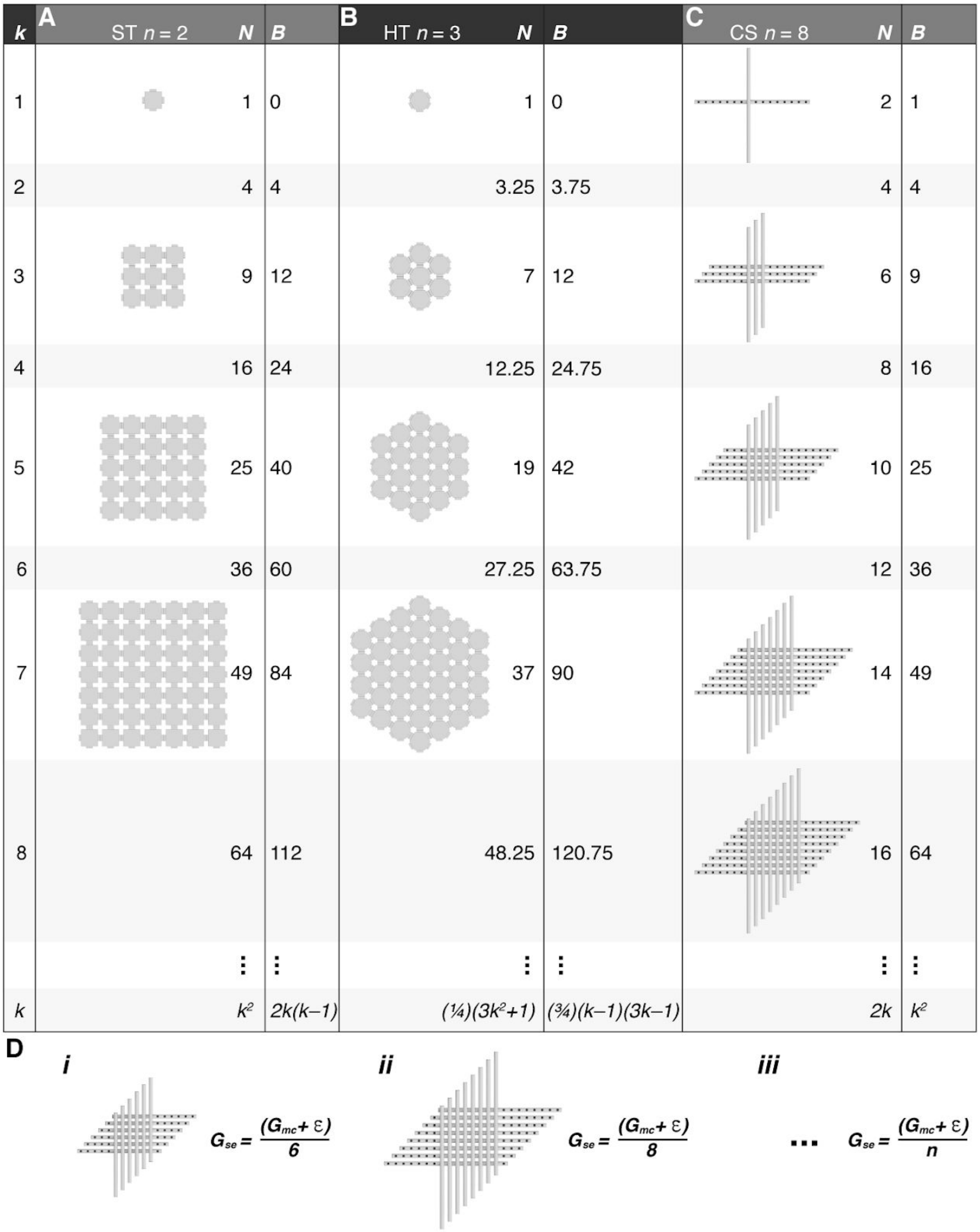

Supplementary Figure 2. A–C, Various *k* size assemblies of square tiles (ST), hexagonal tiles (HT), and crisscross slats (CS), as adapted from Evans and Winfree (Ref [23], see "Section 4 Nucleation, Figure 8" of their work). Expressions defining the number of *N* monomers and *B* bonds for each *k* assembly are unique to a given monomer design, as in the bottom row. Any *k* assembly could represent a critical nucleus for

growth with particular reaction conditions (i.e. a specific value of ε). Models of the nuclei are drawn for all designs with odd values of k. **D**, Relationship between G_{se} and G_{mc} and the size of the maximum critical nucleus (k^*) is dependent on the DNA slat length, with $n = 6$ in ***i***, $n = 8$ in ***ii***, and n is any positive integer in ***iii***.

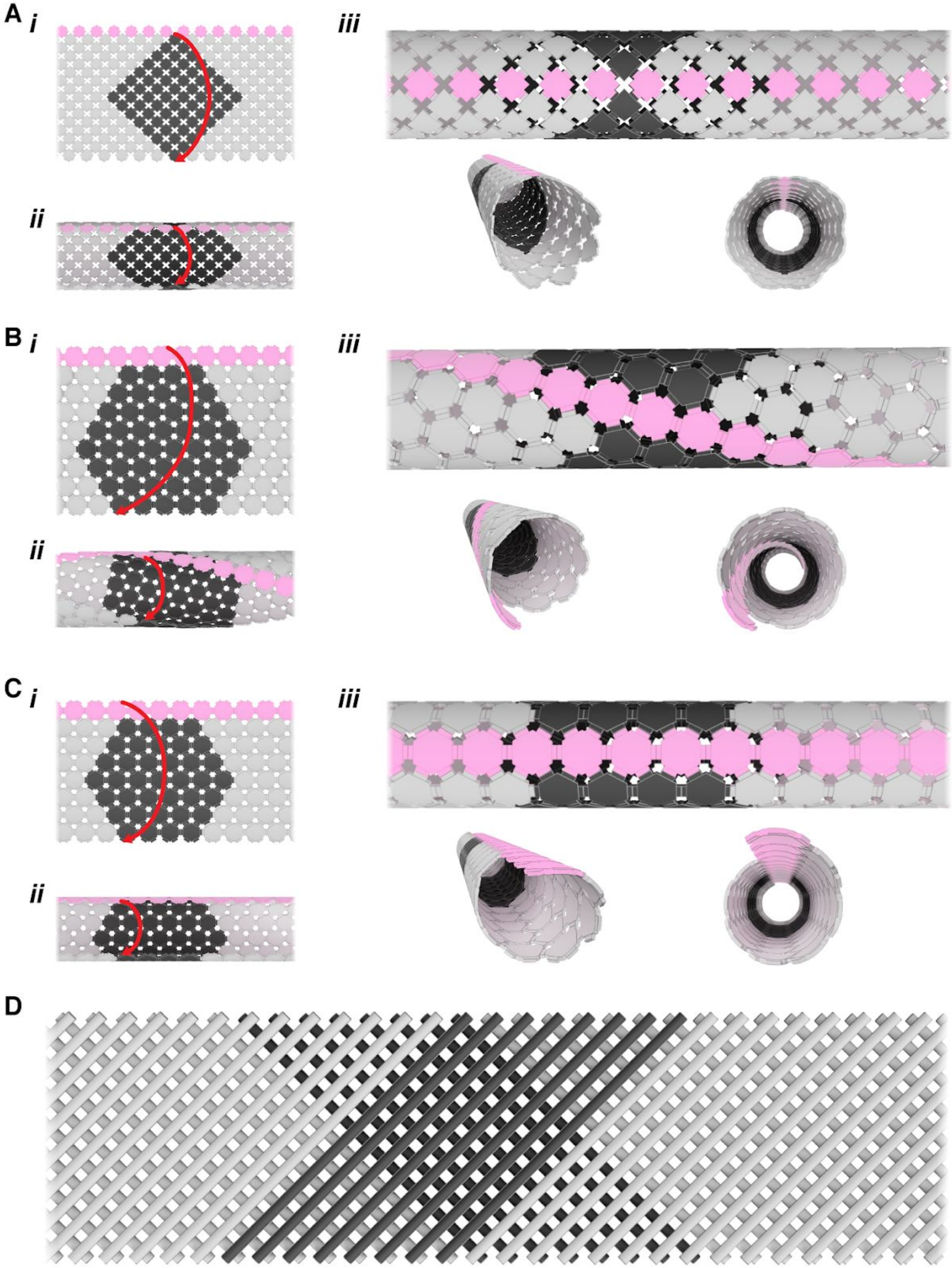
A
i
ii
iii
B
i
ii
iii
C
i
ii
iii
D

Supplementary Figure 3. The critical nucleus for ST and HT and formation of tubes (**A–C**), versus the critical nucleus for CS ribbons (**D**) are shown as dark grey monomers in the context of the larger structure. Magenta tiles are the connecting seam of tiles that allow a planar 2D array of tiles to make a tube. The lighter grey monomers are the growth of a larger structure following nucleation. **A**, the ST k_{max} = 8 critical nucleus is linked into a tube by rolling it corner-to-corner and binding it together with a straight seam of magenta tiles. **B**, the HT k_{max} = ~8 critical nucleus is linked into a tube by rolling it approximately corner-to-corner and binding it together with an angled seam of magenta tiles. **C**, the HT k_{max} = 7 critical nucleus is linked into a tube by rolling it edge-to-edge and binding it together with a straight seam of magenta tiles. The manner in which edges are connected versus the corners as shown ***B*** are an alternative arrangement of HT as a tube that also results in a leading edge suitable for sequential growth with n = 3 bonds as shown in **Supplementary Figure 4**. **D**, the CS k_{max} = 8 critical nucleus grows into a ribbon directly from the nucleus without a seam. We note that the $G(A)$ of the critical nuclei for the tubes in ***A*** and ***B*** is identical to that of the n = 8 CS in D at reversibility when $\varepsilon \rightarrow 0$—the corresponding energies of the nucleus and its size as growth becomes irreversible (i.e. increasingly large values of ε) are plotted rightward in **Supplementary Figure 1C** and **Supplementary Figure 5**. For simplicity, we assume the energetic cost for rolling up the critical nucleus into a tube is negligible.

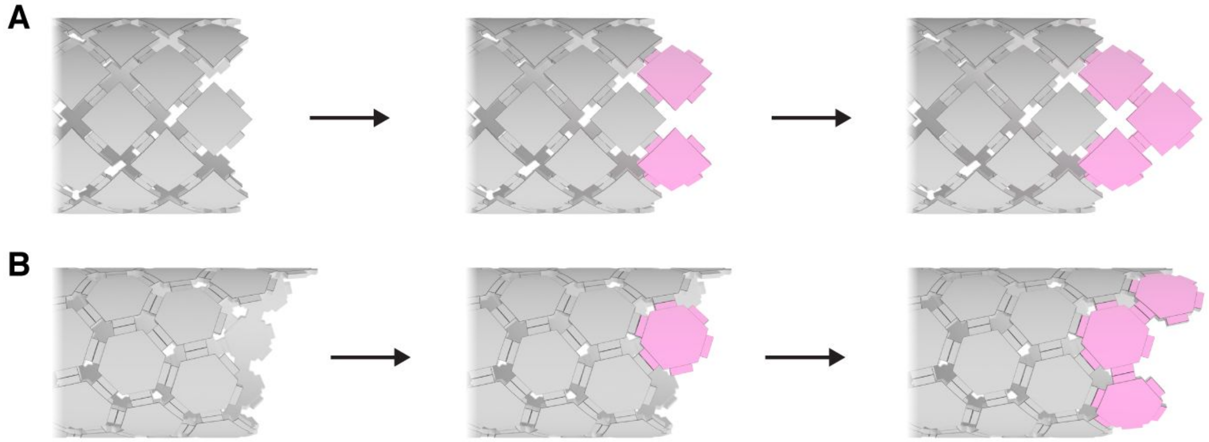

Supplementary Figure 4. The addition of three inbound magenta tiles to perpetuate growth of tubes with a sequential assembly mechanism using bonds to neighbors. In the leftmost of **A**, the leading edge of the ST tube as illustrated in **Supplementary Figure 3A** coordinates $n = 2$ neighbors to bind new tiles. In the leftmost of **B**, the leading edge of the HT tube as illustrated in **Supplementary Figure 3B** coordinates $n = 3$ neighbors to bind new tiles. Newly bound tiles in the middle step coordinate additional tile sites with n bonds to recruit more tiles yet, as shown in the rightmost steps. We contrast the tile addition here with CS in **Figure 2A*ii*** where slats are added sequentially by binding any designed natural number n neighbors. With assembly of CS ribbons, each addition of a slat in one orientation (e.g. an x-slat) is followed by the addition of another slat in the perpendicular orientation (e.g. a y-slat).

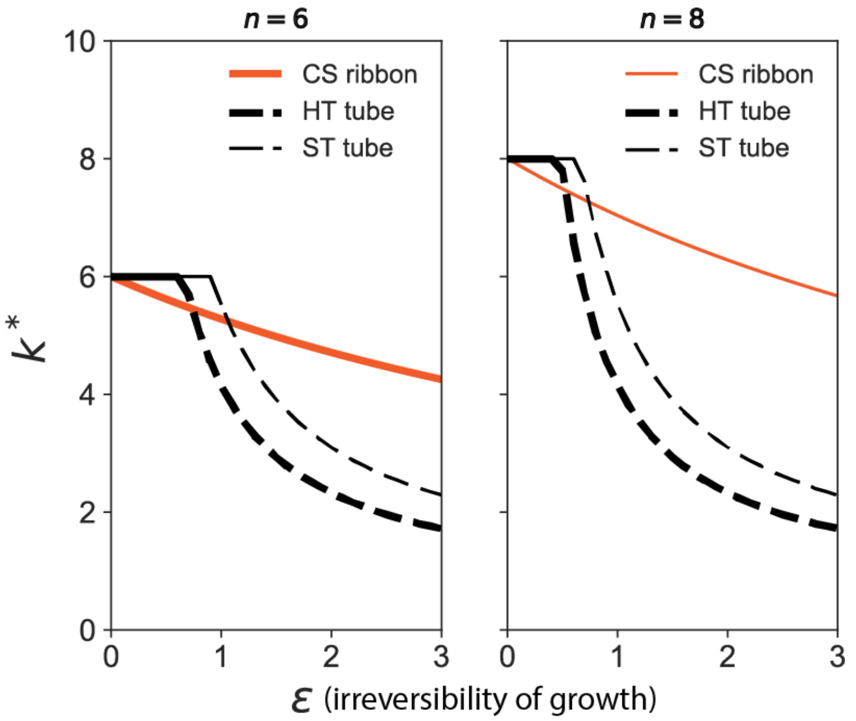

Supplementary Figure 5. Critical-nucleus size k^* versus ε for ST- and HT-nanotube and crisscross assembly, shown for $n = 6$ (left) and 8 (right, i.e. $k_{max} = 6$ and 8). Free energy of the monomer concentration $G_{mc} = 1.3 - log_{10}\left(\frac{10^{-6}M}{1M}\right) = 7.3$ (i.e. 1 µM each DNA slat or square tile monomer and an effective concentration of bound slats or square tiles of 20 M (i.e. $\alpha = \log_{10}(20\text{ M}/1\text{ M})$).

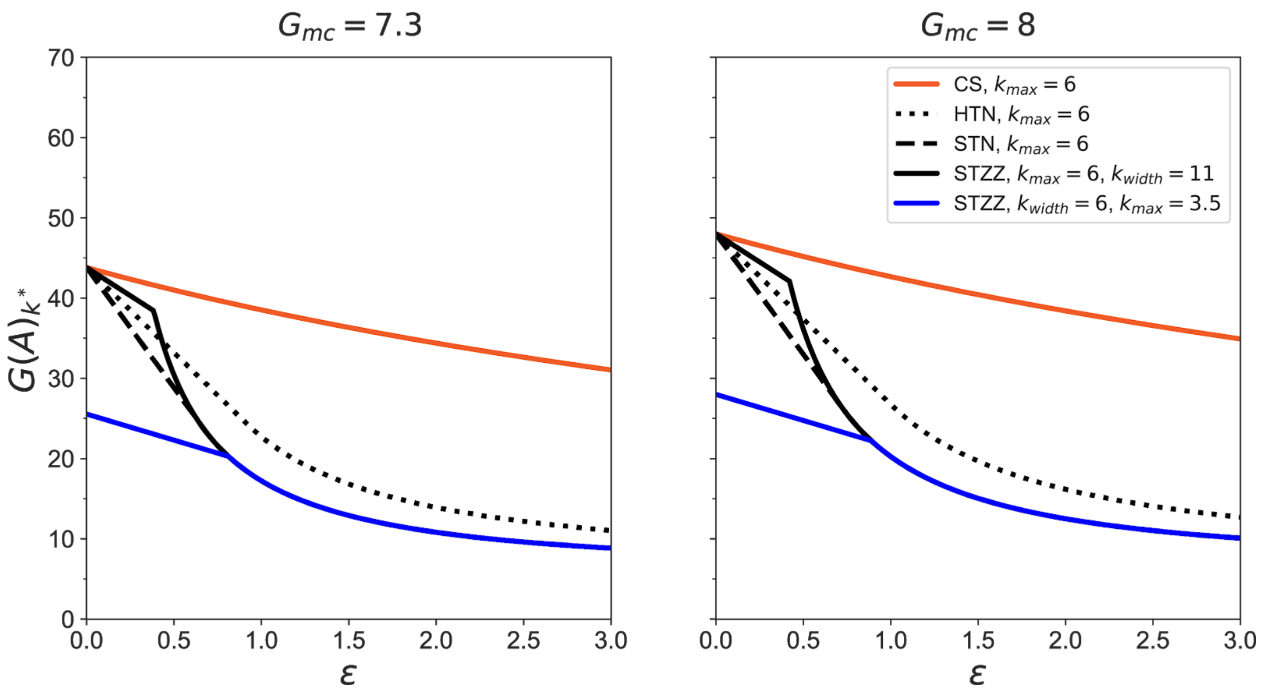

Supplementary Figure 6. Free energy *G(A)* at the critical-nucleus k^* versus ε for ST-nanotubes (STN), ST-zig-zag ribbons (STZZ), HT-nanotube (HTN), and crisscross (CS) assembly, shown for $G_{mc} = 7.3$ (left) and *8* (right). The derivations for the equations that describe STN, HTN, and CS ribbons can be found in S2.1 and for STZZ in S2.2. For STZZ, we normalized $G(A)_{k^*,STZZ}$ as $k_{max}G_{mc}$ at $\varepsilon \rightarrow 0$, in order to better compare STZZ to STN, HTN, and CS ribbons. To do this for STZZ, we redefined $k_{max} = 0.5(k_{width} + 1)$ or $k_{width} = 2k_{max} - 1$.

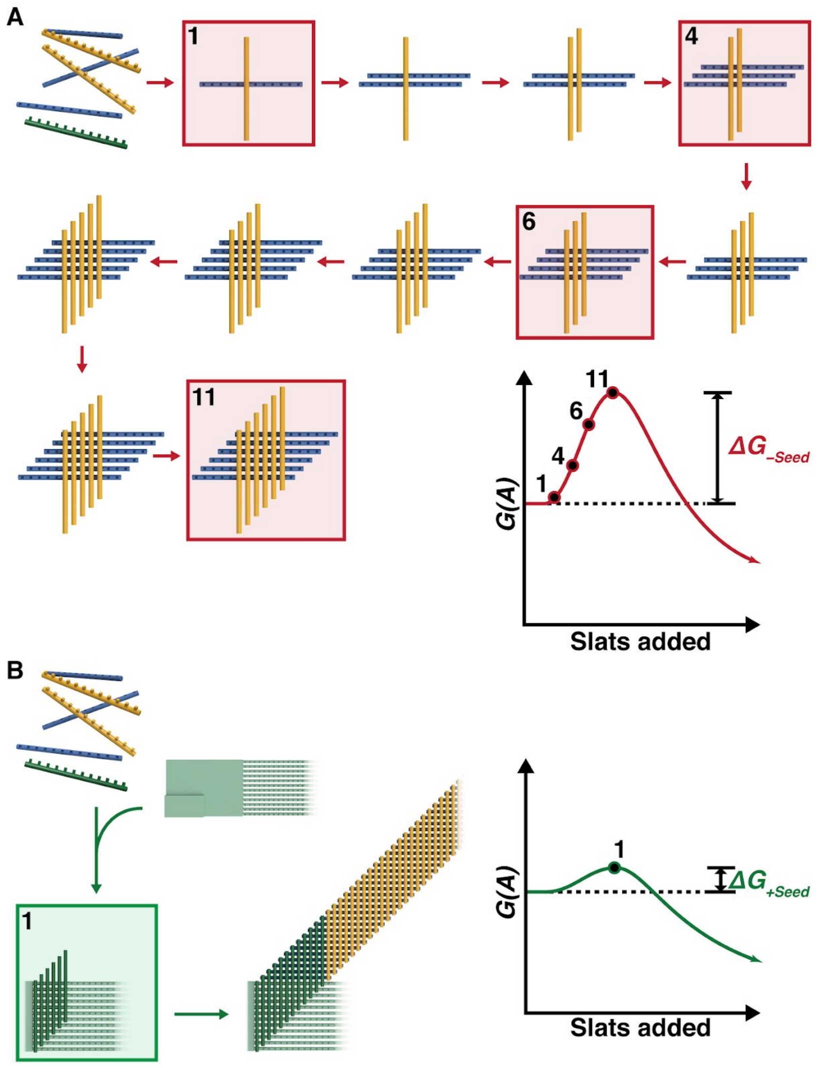

Supplementary Figure 7. Qualitative energetic landscape for spontaneous versus seed-initiated nucleation of crisscross polymerization. **A**, Spurious nucleation must overcome a large kinetic barrier to assembly. In the absence of a seed, DNA slats must form a rate-limiting critical nucleus, shown in step 11, before the capture of new slats to the end of ribbons is energetically favorable. The leading edge of the critical nucleus has sufficiently many overhanging binding sites to cooperatively engage new slats. All steps prior to 11 are energetically unfavorable because the entropic penalty of recruiting slats is larger with respect to energetic gains of binding single or small numbers of weak half-turn binding sites. **B**, Seed-initiated nucleation provides an alternate lower barrier route to assembly. The seed has multiple single stranded binding sites fixed proximally to cooperatively engage initial nuc-y-slats, as shown in step 1. The kinetic barrier to nucleation is much lower with the seed and repeating sets of x- and y-slats are now added to grow ribbons.

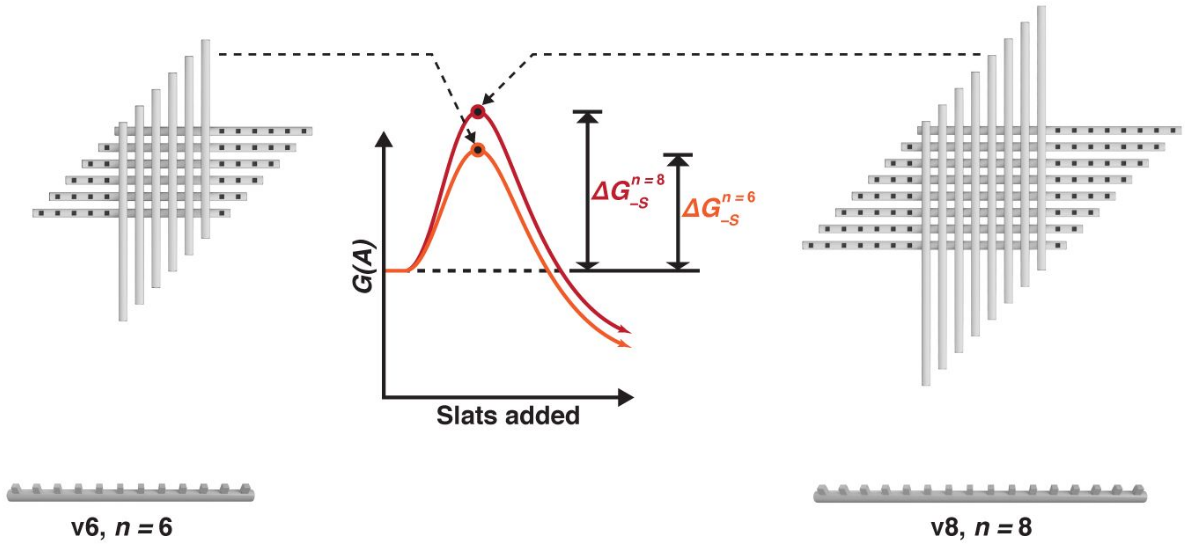

Supplementary Figure 8. Critical nuclei for *v6* (n = *6*) versus *v8* (n = *8*) slat assemblies. ΔG_{-} denotes the energetic barrier without a seed being present in the assembly. The maximum barrier height for crisscross polymerization is proportional to n, therefore the barrier for v8 (n = 8) is 33% higher than that of v6 (n = 6). The size of the critical nucleus is made larger by using longer slats with more stringent assembly conditions. There are four more sequentially arranged binding sites per slat with v8 versus v6 slats. More binding energy is therefore available to capture new slats at the end of v8 slat ribbons. The consequence is that optimal growth is attainable with conditions less prone to spurious nucleation, such as using higher reaction temperatures. The v6 versus v8 slats have critical nuclei that are 6+6 and 8+8 arrays of x- and y-slats respectively, so long as assembly is carried out at the appropriately high near-reversible temperatures. In principle, DNA slats could be further elongated to increase the kinetic barrier to assembly using reaction conditions more stringent than characterized here (e.g. lower $MgCl_2$ concentration, higher temperature, presence of chemical denaturant such as formamide, etc).

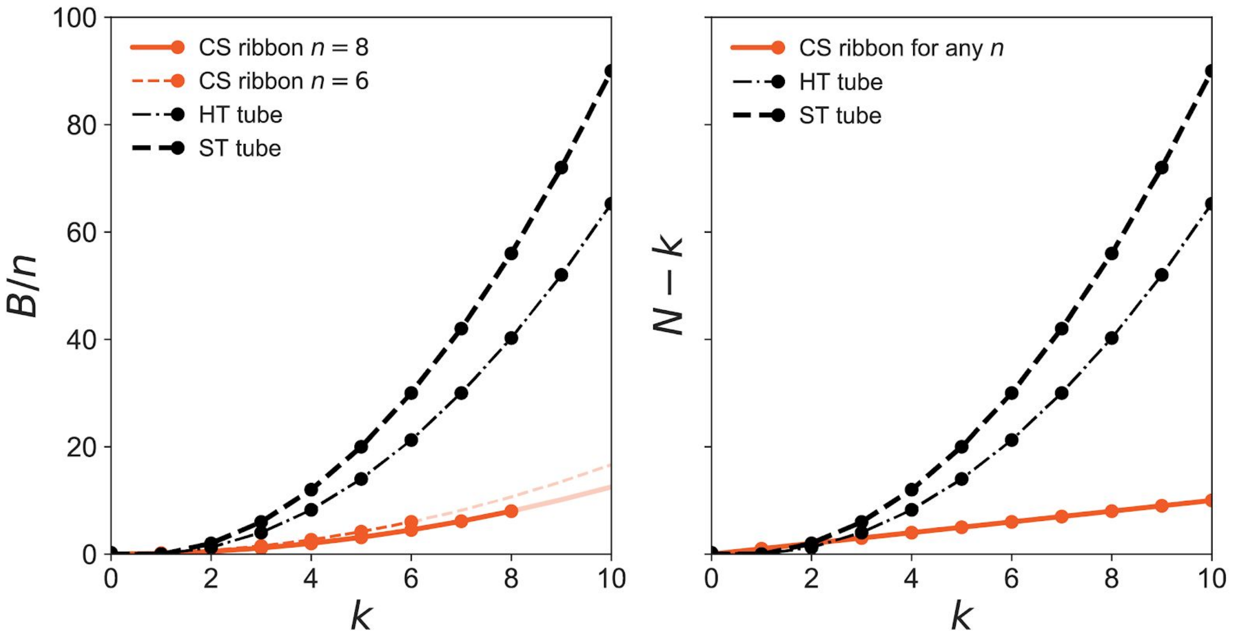

Supplementary Figure 9. On the left, a plot of the number of reversible-growth bond credits B/n, in units of G_{mc}, versus the width of compact assembly k comparing ST to HT to CS compact assemblies. On the right, a plot of the difference of number of monomers and width of compact assembly $N - k$ versus width of compact assemblies k. For ST and HT (black lines), the number of pairs of unbonded coordination sites $nN - B$ equals nk, therefore $N = B/n + k$ (i.e. left and right plots overlap since $B/n = N - k$). In other words, in units of G_{mc}, the total debits N equals the sum of those paid by reversible-growth bond credits B/n and those that are outstanding k. For CS (red lines), $nN - B = nk$ only for $k = n$; $nN - B > nk$ for $k < n$, therefore $N - k > B/n$ for $k < n$. For the plot on the left, $k > n$ is rendered as translucent since this is not physically possible.

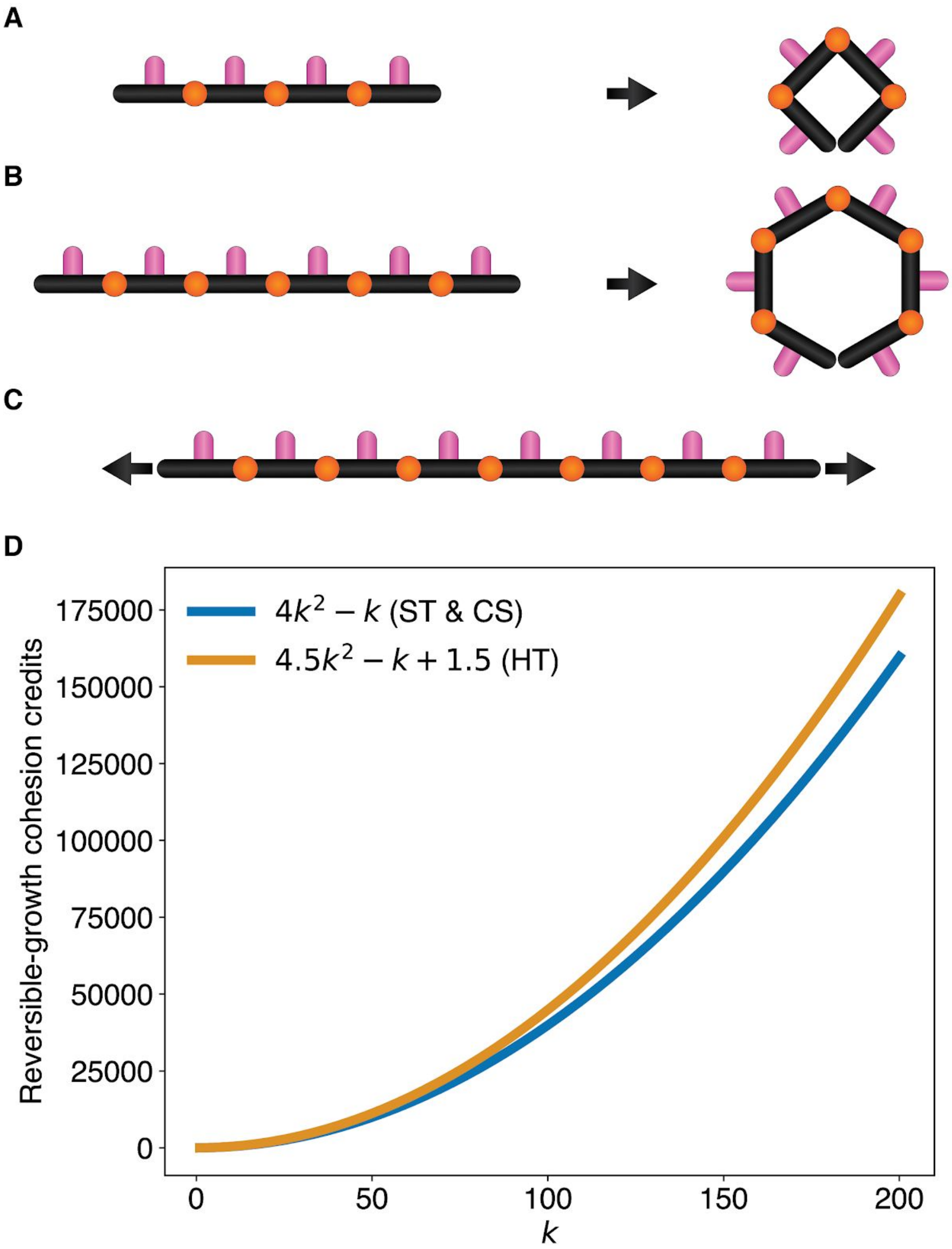

Supplementary Figure 10. A–C, Toy model representation showing the arrangement of *2n* nodes into ST, HT, and CS respectively. Magenta denotes coordination sites and orange denotes intra-monomer linkages. **D**, Number of reversible-growth cohesion credits (i.e. $B/n + N(2n - 1)$), in units of $-G_{nc}$, versus k. The legend shows simplified expressions for ST, HT and CS (for ST and CS, despite different n, B, and N, the expressions reduce to be identical).

Supplementary Figures 11–13: ssDNA slat architecture

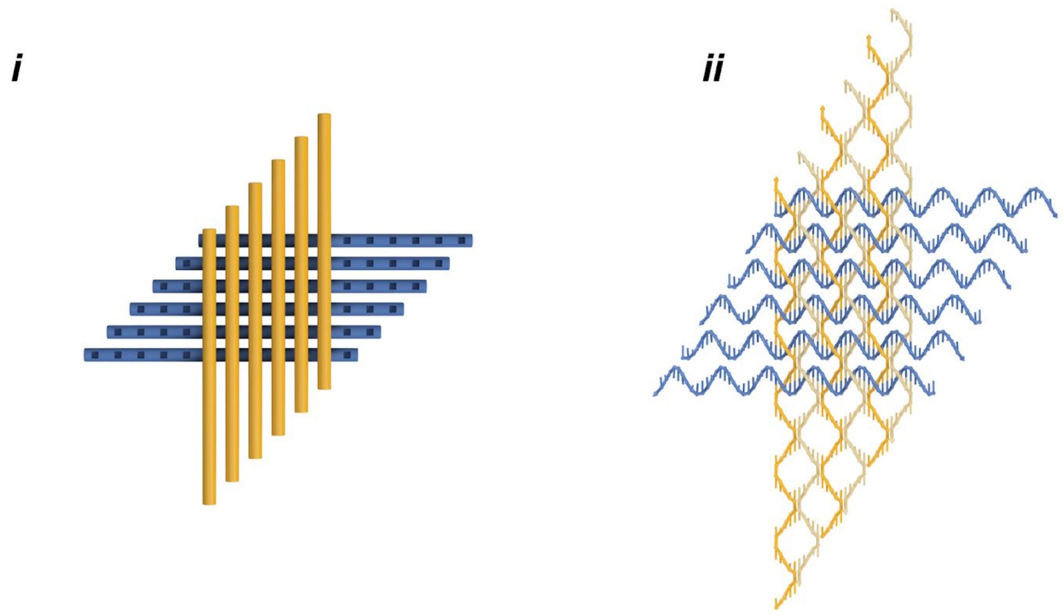

Supplementary Figure 11. Critical nucleus for $CS_{n=6}$ (v6) sequence variants is composed of 12 individual slats. ***i***, generalized cartoon model. ***ii***, specific implementation for ssDNA slats as shown with ball-and-stick DNA model. Each slat blue x-slat or gold y-slat is comprised of 12 half-turns of DNA, with all binding sites as shown in this particular model as 5 nt. However, sequences tested in this work also contained 6 nt binding sites. We note that a crisscross-like routing of the scaffold strand resulting in the staggered parallel double helices connected by antiparallel crossovers that occur every half turn, has previously been reported in a DNA origami structure (see Figure 4B of ref [30]).

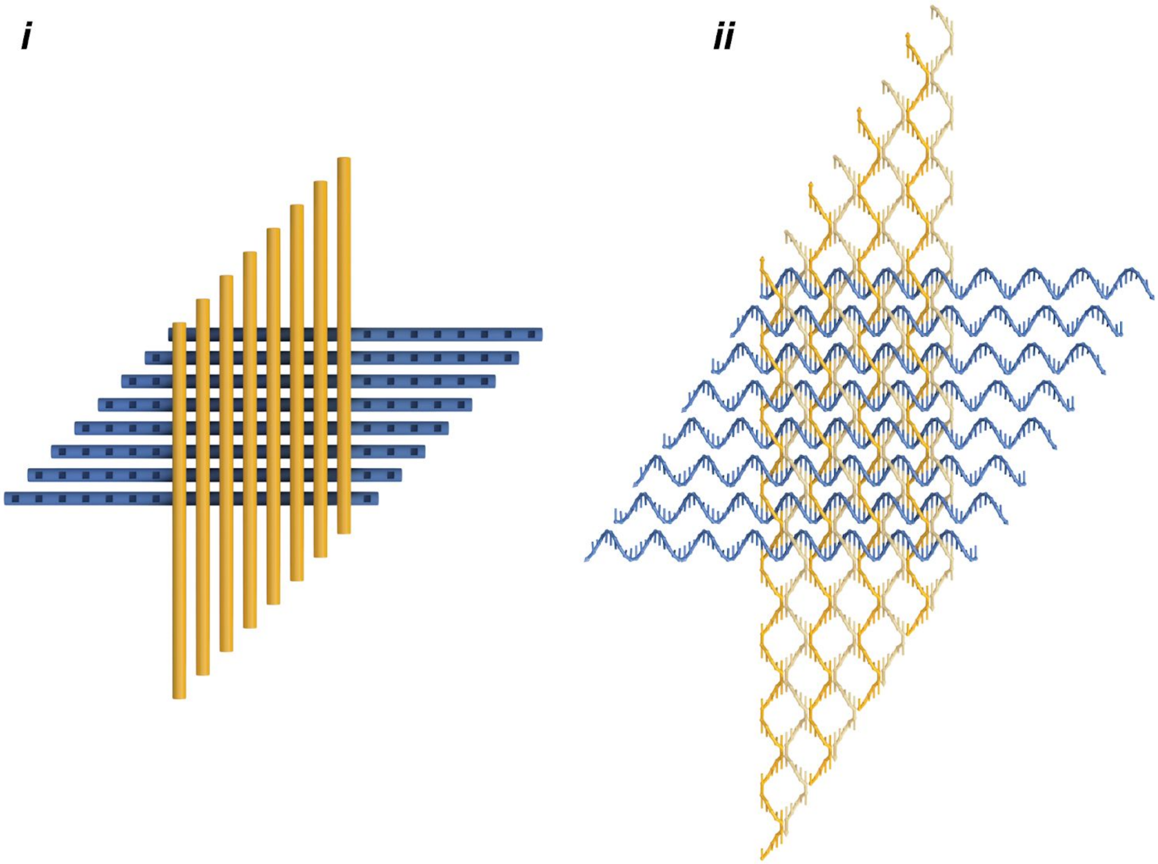

Supplementary Figure 12. Critical nucleus for $CS_{n=8}$ (v8) sequence variants is composed of 16 individual slats. ***i***, generalized cartoon model. ***ii***, specific implementation for ssDNA slats as shown with ball-and-stick DNA model. Each slat blue x-slat or gold y-slat is comprised of 16 half-turns of DNA, with all binding sites as shown in this particular model as 5 nt. However, sequences characterized in this work also contained 6 nt binding sites.

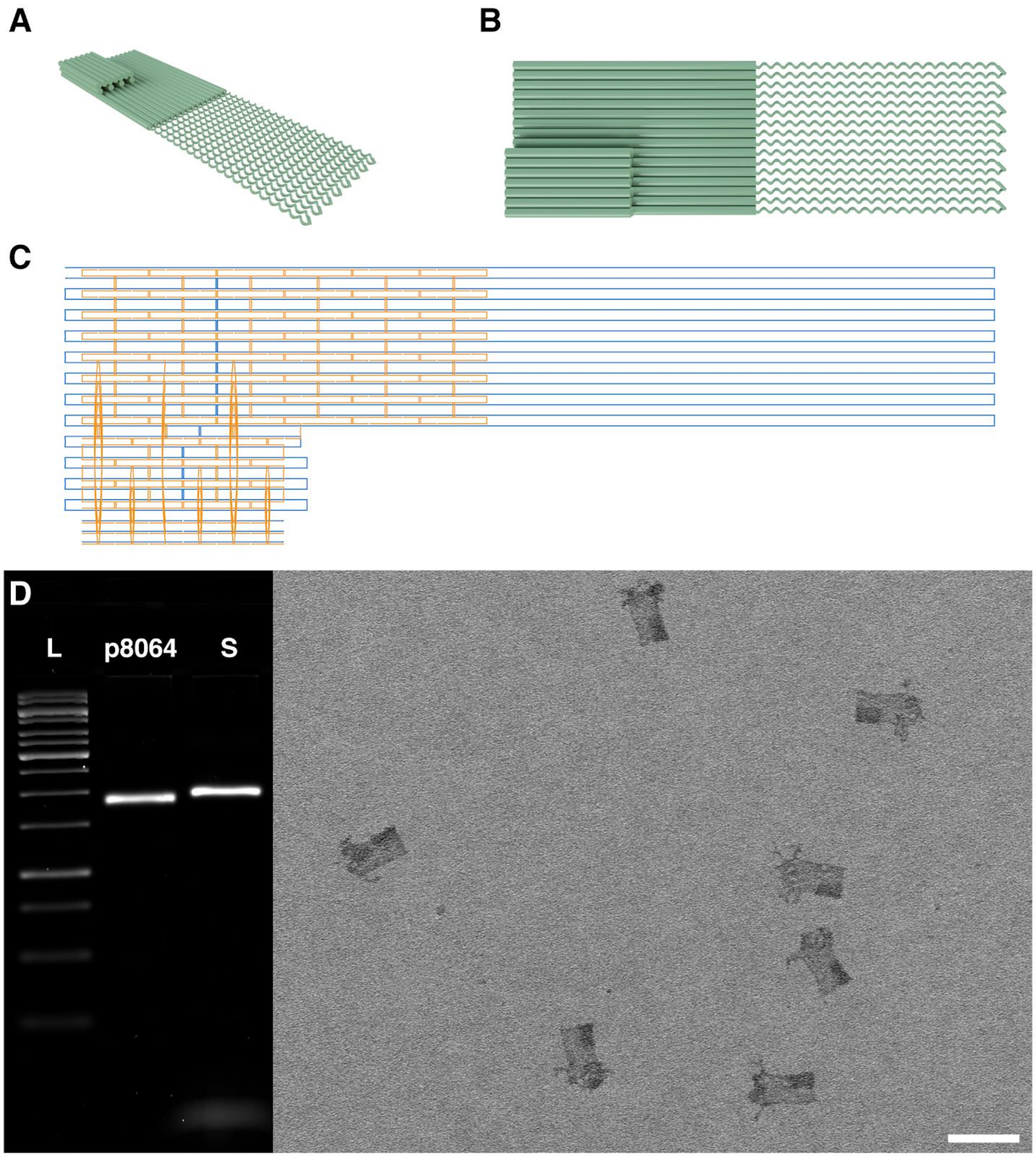

Supplementary Figure 13. DNA origami seed design and assembly. **A–B**, rendering of cylindrical extrusion of design from corner and top views respectively. Sixteen bare scaffold DNA loops that bind the nuc-y-slats are shown as twisted thin cylinders, versus the dsDNA body and reference square shown as straight thick cylinders. **C**, DNA origami scaffold routing diagram as generated with caDNAno[31] with the bare scaffold shown in the right-half of the diagram. **D**, Agarose-gel characterization (left) of folded DNA origami seed (S), with 1 kb Ladder (L), and M13 p8064 scaffold. TEM micrograph (right) of folded DNA origami seeds. Note that bare ssDNA scaffold loops appear unstructured and twisted. Scale bar is 100 nm.

Supplementary Figures 14–22: Determining optimal growth conditions for seeded ribbons

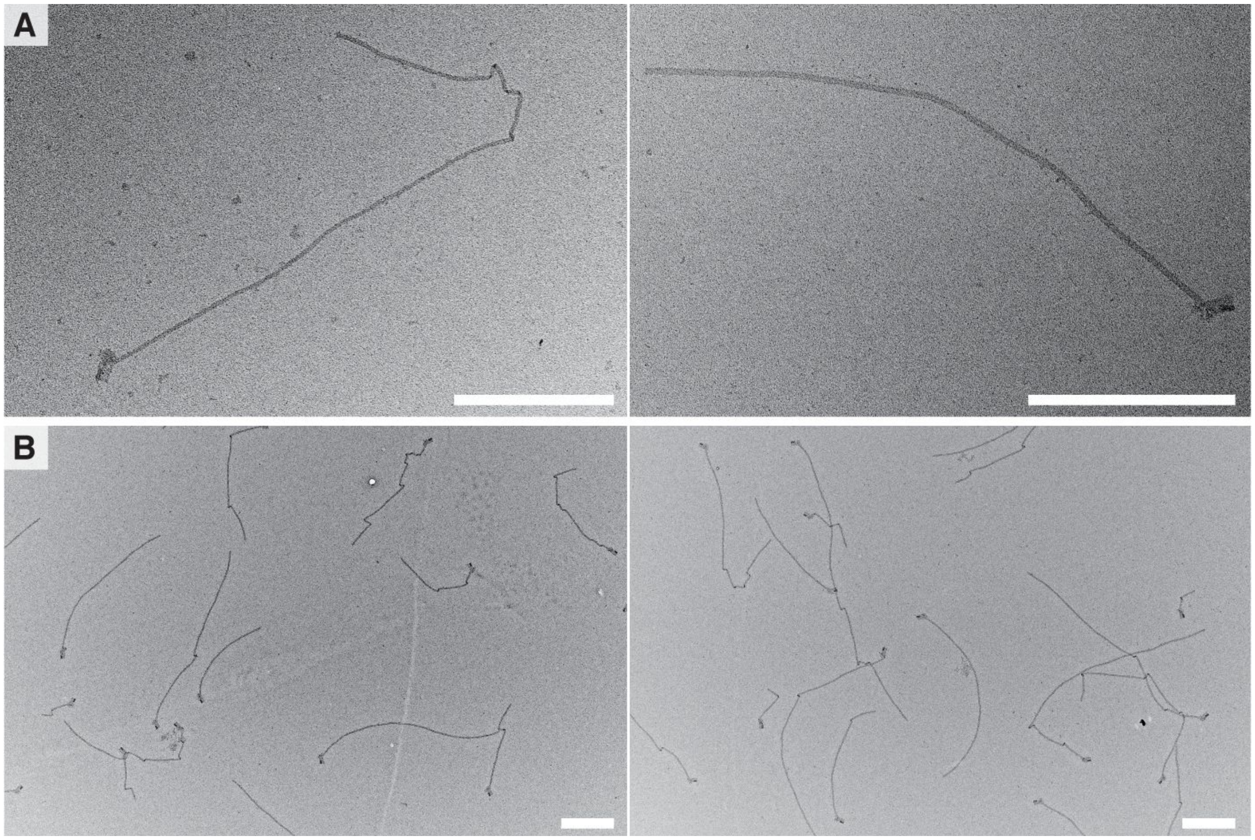

Supplementary Figure 14. TEM images depicting seeded growth of v6.1 ribbons. Growth was conducted for ~16 hours at 50°C, with 14 mM $MgCl_2$, 2 nM seed, and 0.2 µM each slat. Seeds are visible on all structures shown. **A**, Close-up of single ribbons. Left ribbon is ~2.3 µm long, and the right ribbon ~1.5 µm long. **B**, Lower magnification shows population of ribbons. Scale bars are 500 nm.

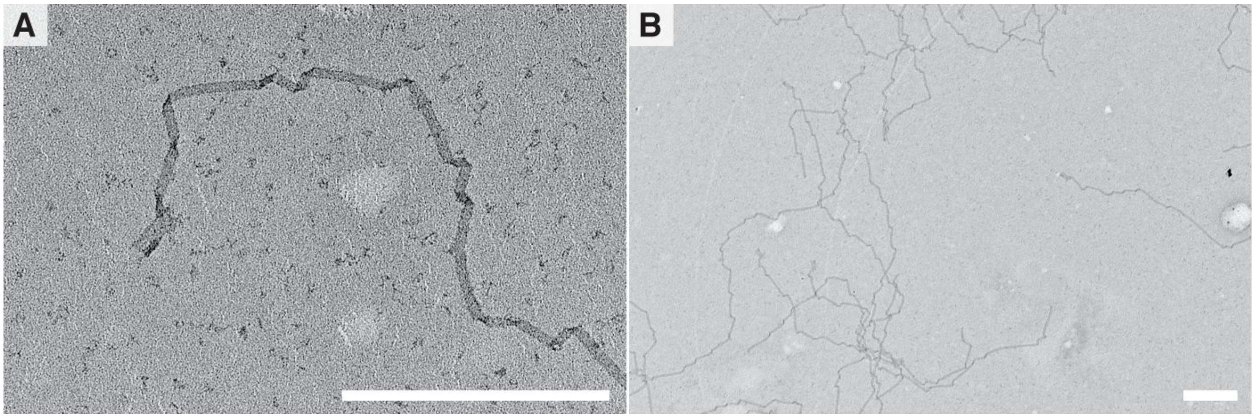

Supplementary Figure 15. TEM images depicting seeded growth of v8.2 ribbons. Growth was conducted for ~16 hours at 50°C, with 16 mM MgCl2, 2 nM seed, and 0.5 μM slats. Seeds are generally visible on structures shown. **A**, Close-up of single ribbon with ~1.6 μm length of growth shown. **B**, Lower magnification shows population of overlapping ribbons. Scale bars are 500 nm.

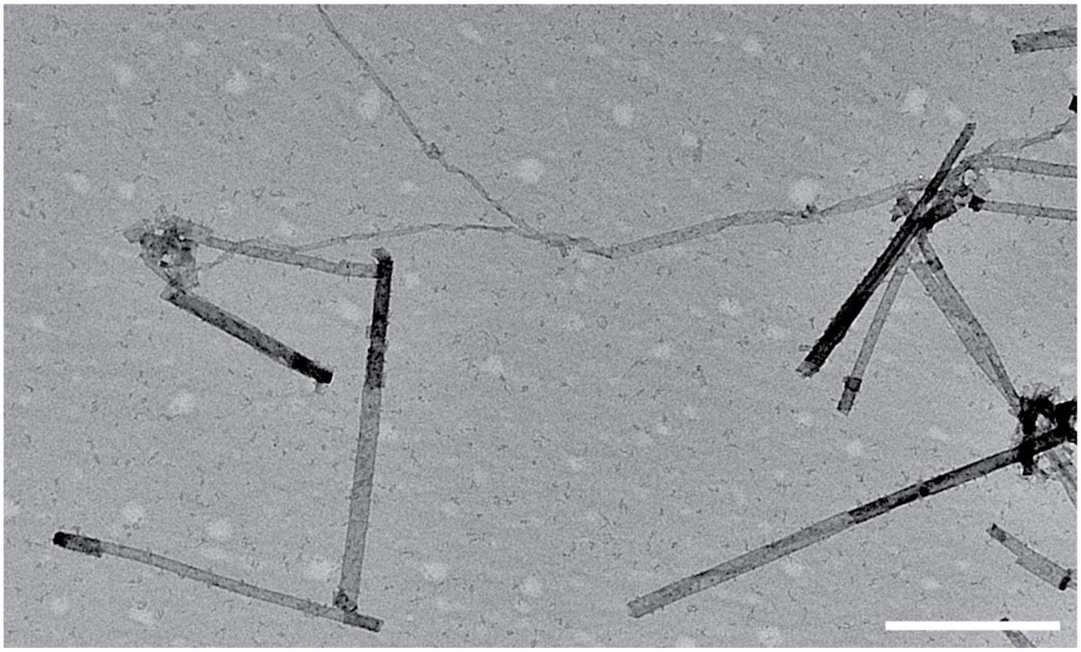

Supplementary Figure 16. TEM images depicting seeded growth of the high symmetry v8.7 slats. These ribbons are prone to aggregation, because they lack T-brush passivation on the ends of the slats (see **Supplementary Table 5**). We hypothesize that they aggregate by blunt-end stacking of the ribbon edges. This particular design uses 10 bp/turn, which also alters ribbon morphology (see **Supplementary Figure 41** for more details on organization of binding domains, and **Supplementary Figures 42–44** for how such rearrangements influenced the resulting morphology. See **Supplementary Table 5** indicating the locations of T-brushes for various designs). Scale bar is 500 nm.

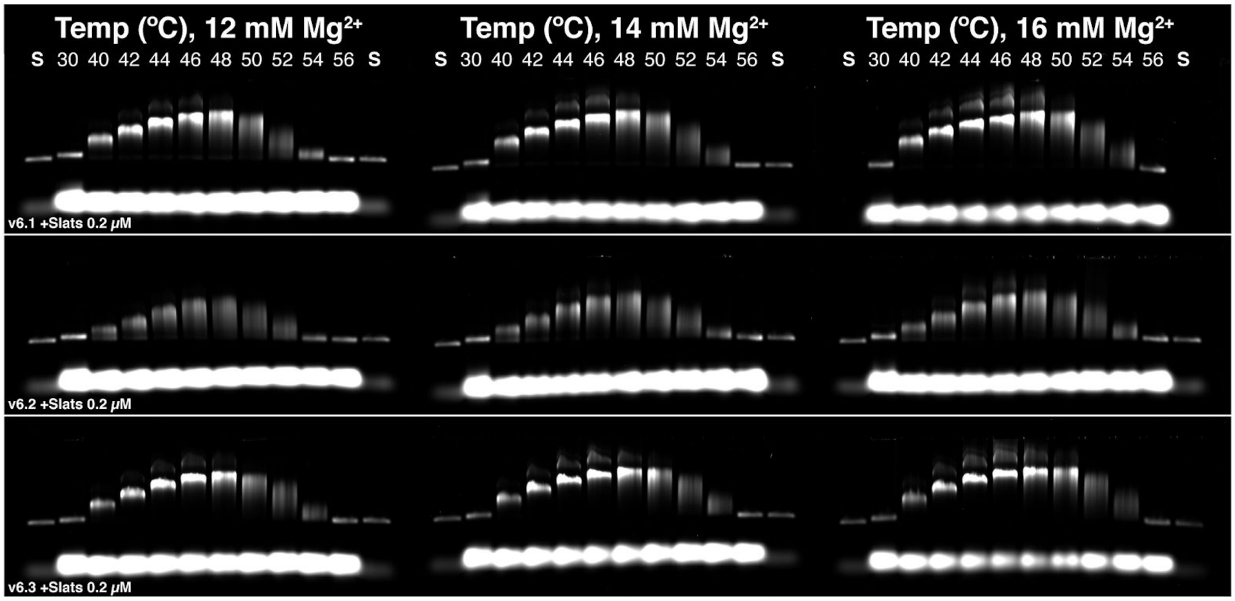

Supplementary Figure 17. Agarose-gel characterization of seeded growth of v6.1, v6.2, and v6.3 to find optimal seeded growth temperatures and $MgCl_2$. Growth was conducted for ~16 hours at the temperature listed above each lane (12, 14, or 16 mM $MgCl_2$, 0.2 µM each slat, 2 nM seed, ~16 hours growth). Lane (S) is the seed only control. Note that the overall slat population was significantly depleted for v6.3 16 mM $MgCl_2$ at the optimal temperature, even though the nuc-y-slats (i.e. ⅓ of the added total) could not be consumed. Temperature screen for v6.1 was performed twice with similar results and once for v6.2 and v6.3.

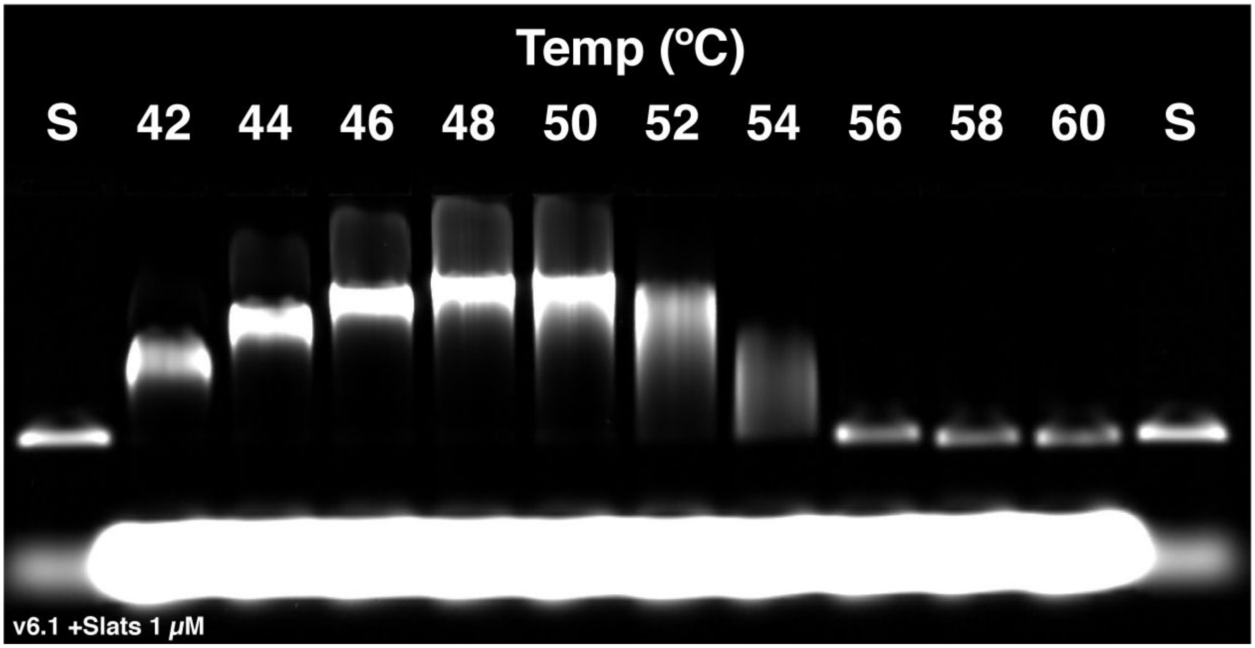

Supplementary Figure 18. Agarose-gel characterization of seeded growth of v6.1 to find optimal seeded growth temperature with higher concentration of slats (16 mM $MgCl_2$, 1 µM each slat, 2 nM seed, ~16 hours growth). Lane (S) is the seed only control. This experiment was performed once.

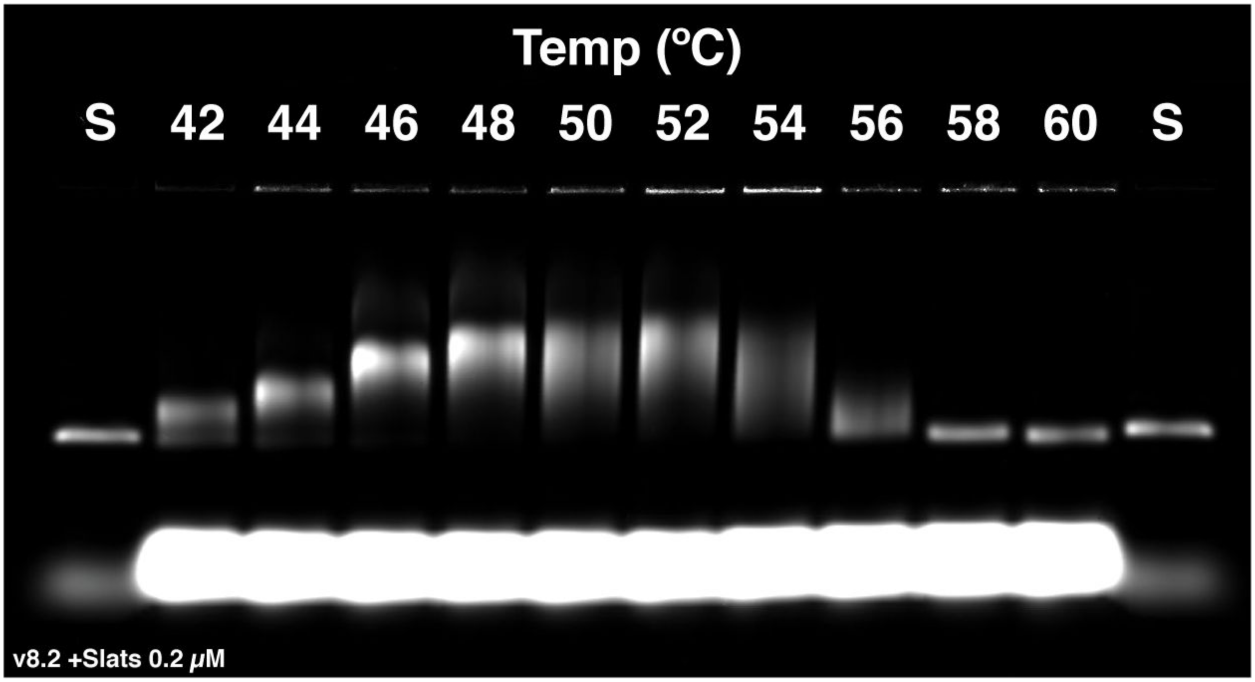

Supplementary Figure 19. Agarose-gel characterization of seeded growth of v8.2 to find optimal seeded growth temperature (14 mM $MgCl_2$, 0.2 µM each slat, 2 nM seed, ~16 hours growth). Lane (S) is the seed only control. This experiment was performed once.

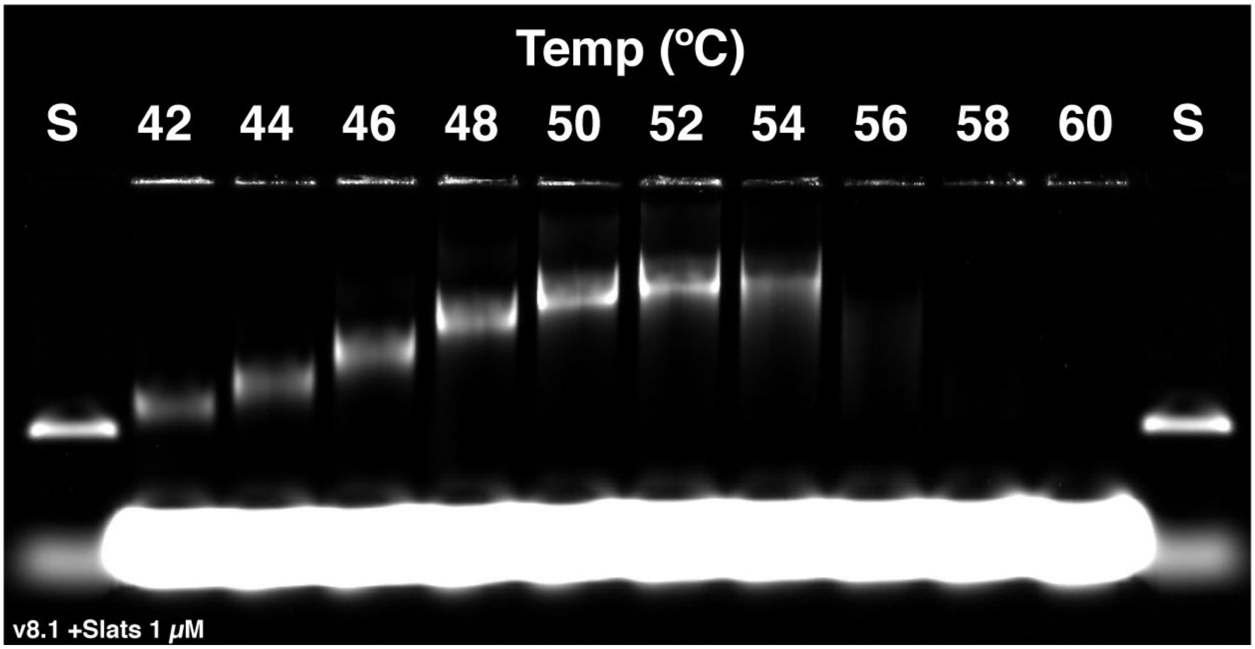

Supplementary Figure 20. Agarose-gel characterization of seeded growth of v8.1 to find optimal seeded growth temperature with high concentration of slats (16 mM $MgCl_2$, 1 µM each slat, 2 nM seed, ~16 hours growth). Lane (S) is the seed only control. Long filaments of this design appear to aggregate at temperatures above 54ºC; presumably this could be mitigated with even longer T-brushes for greater passivation. This experiment was performed once.

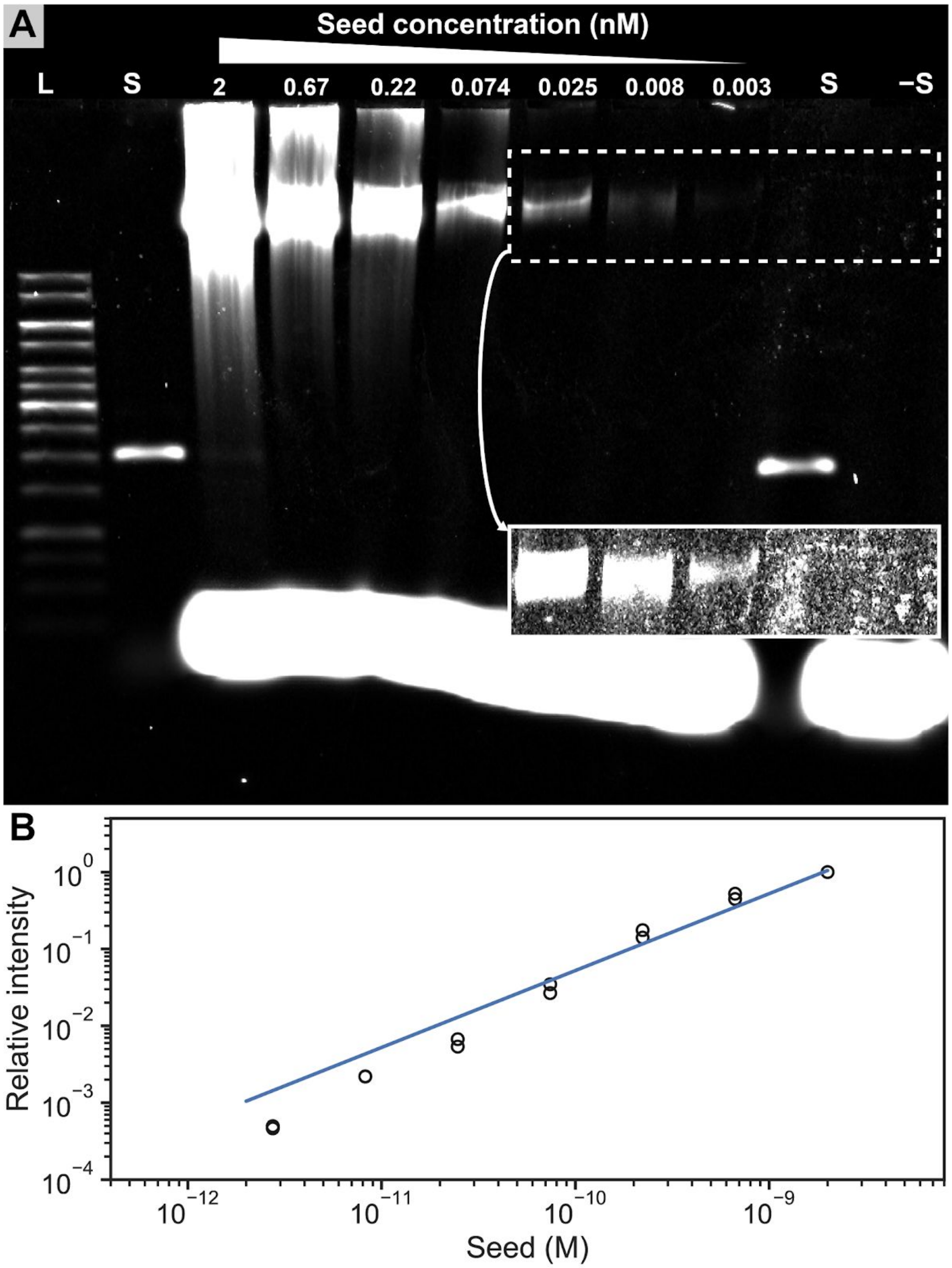

Supplementary Figure 21. Growth of v6.1 ribbons with different initial concentrations of seed. Growth was conducted for ~12 hours at 50°C, with 16 mM $MgCl_2$ and slats at 1 μM each (condition where no spurious assembly is observed). Insert shows contrast and brightness adjusted gel for better visualization. We expect the copy number of ribbons in the final reaction to be linearly proportional to the number of seeds added to the starting reaction. This property of DNA slats is analogous to DNA origami in how the number of scaffold strands precisely controls the copy number of folded structures. **A**, Agarose gel of a single replicate of experiment. Lane (L) is the ladder and (S) is the seed only control. **B**, Plot of relative gel densitometry (measured with respect to the conditions with the highest 2 nM concentration of seed) versus initial concentration of seed in duplicate experiments, with the line showing a linear fitting of the data.

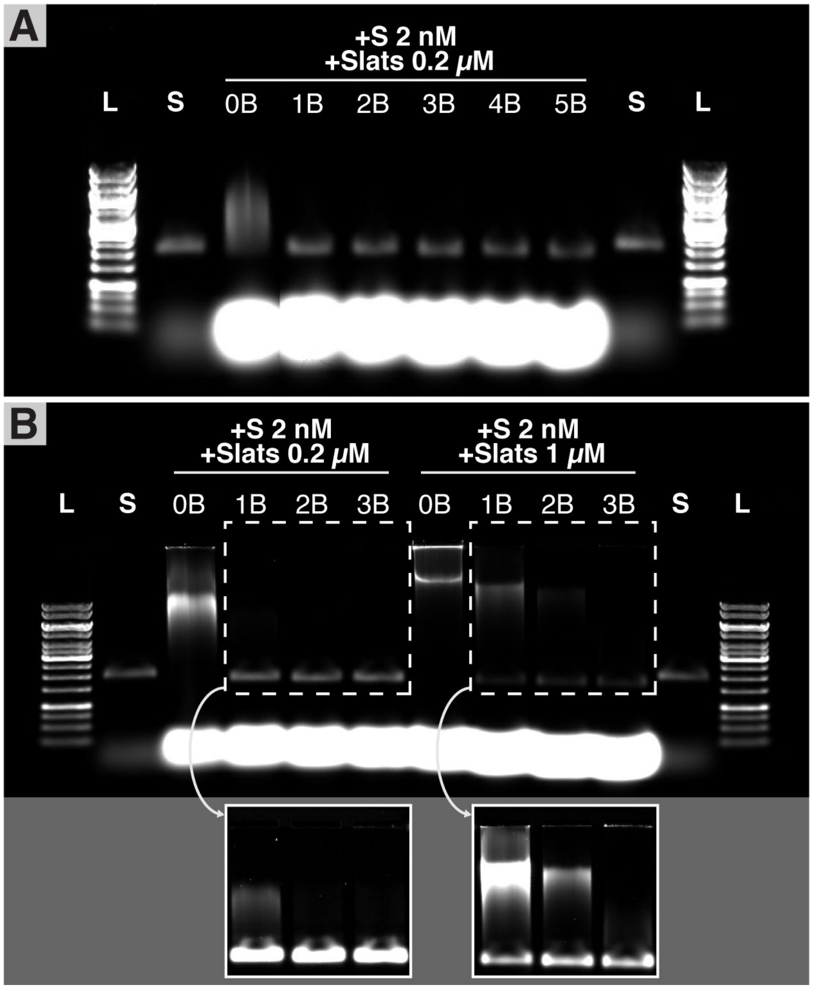

Supplementary Figure 22. We hypothesized that ribbon assembly requires sequential addition of every single designed slat for growth. To test whether ribbon growth could be attained if specific slats in assembly mixture were impeded, we added a 5-fold excess of blocking strands (i.e. reverse complement sequences of a slat) to mask one or more specific nuc-y slats. Here we show that blockage of only a single nuc-y slat completely terminated ribbon growth with 0.2 μM each slat. Using a higher concentration (i.e. 1 μM each) of slats and lower temperature allowed limited recovery of growth (i.e. skipping over the impeded nuc-y slats) for when up to two nuc-y slats were blocked, although growth was completely terminated when three nuc-y slats were blocked. DNA slats assembly was done with sequence v6.1 and blocking strands (B) added at 5X molar excess under various assembly conditions. **A**, DNA slats assembly with 0.2 μM each slat and 2 nM seed, in 12 mM $MgCl_2$ at 50ºC for ~16 hours. No blocking strand (0B) and 1–5 blocking strands (1B–5B, i.e. blocking 1, 2, 3, 4, or 5 nuc-y-slats simultaneously), with each at 1 μM concentration. Only 0B displayed visible growth. **B**, Increasing concentration of the slats from 0.2 μM each (left) to 1 μM (right) with 2 nM seed, in 14 mM $MgCl_2$ at 45ºC for ~16 hours. Blocking strands were added at either 1 μM (left) or 5 μM (right). The combination of lower temperature, higher concentration of slats, and more $MgCl_2$ in the right-half of ***B*** was sufficient to allow partial recovery of ribbon growth for blockage of up to two distinct nuc-y strands, as shown in the cut-away contrast and brightness adjusted section of the gel. (L) 1 kb ladder and (S) seed only control. This experiment was performed once.

Supplementary Figures 23–28: Assay for spontaneous nucleation under optimal growth conditions

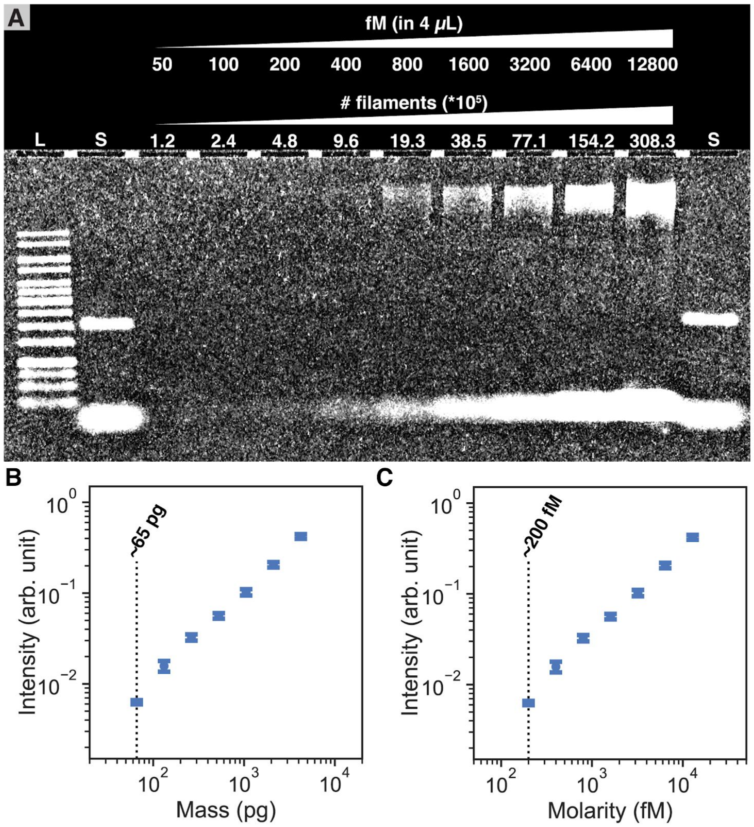

Supplementary Figure 23. To determine the sensitivity limit of SYBR-Gold, we loaded a dilution series of ribbons on agarose gels to determine the minimal concentration where a gel band could be identified. We prepared a typical ribbon assembly reaction (1 µM each v6.1 slat, 16 mM $MgCl_2$, 50ºC for ~16 hr) where 2 nM initial concentration of seed was added. The ribbons were diluted 1 in 250 in 1X reaction buffer. Then, a Labcyte Echo 525 acoustic liquid handler was used to transfer volumes (from 0.025–6.4 µL) of diluted reaction into 10 µL pools of agarose gel loading buffer. The volumes transferred corresponded to the absolute number of ribbons as labeled above each gel well (we assumed one-for-one conversion of each seed into a ribbon). Given the typical 4 µL loading volume of assembly reactions for gel characterization, the absolute loading amounts correspond to the fM molarity annotated above each gel well. A faint, barely discernible band is seen for 200 fM (~$4.8*10^5$ ribbons). We further use the assumption that the mean length of ribbons in the reaction to be ~5 µm based on TEM observations and the model in **Supplementary Figure 29**, such that the picogram mass can be estimated. We assume each slat contributes 1.5 nm to the total length of the ribbon, such that there are an average of ~3333 slats (with each slat comprising 71 nt) extending from each 8064 bp seed. Thus, we calculate that we can detect ribbons to about 65 pg of assembled material (i.e. ~200 fM ribbons in 4 µL reaction). **A**, Agarose gel of a single dilution series, where (L) is the ladder and (S) the seed control, with a faint barely discernible band seen for 200 fM. **B**–**C**, Plots of a triplicate gel dilution series experiment showing mean gel intensities vs estimated mass and molarity of ribbons loaded, with

error bars showing ±SD (for N = 3, independent experiments). The SYBR-Gold detection limits are labeled with a dotted line on each plot.

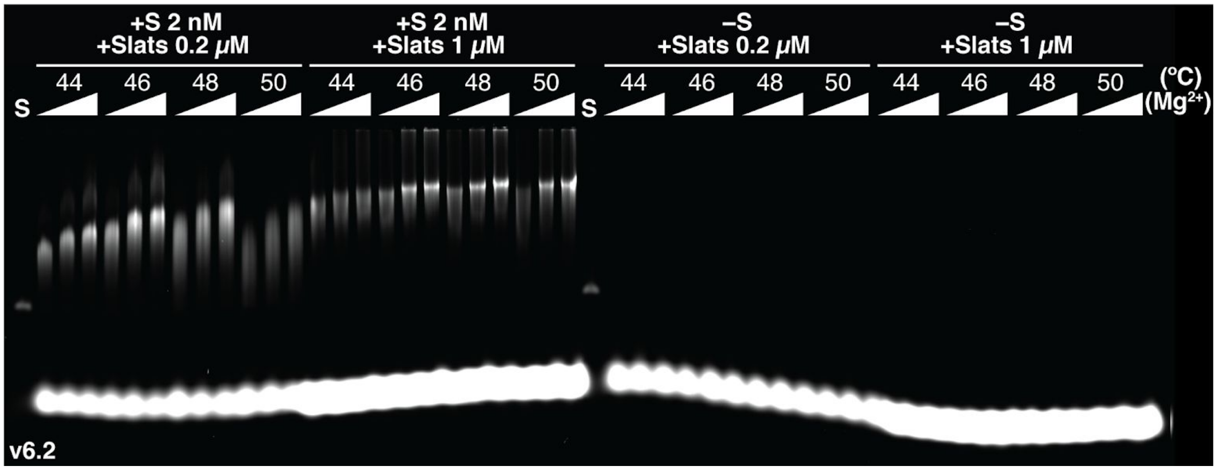

Supplementary Figure 24. Gel characterization of seeded (left) and unseeded (right) growth of DNA v6.2 slats. Reactions with either 0.2 or 1.0 μM each slat were incubated isothermally at one of four temperatures in either 12, 14, or 16 mM $MgCl_2$ for ~16 hours. Notably, unseeded reactions yielded no observable assembly at all conditions tested. (S) is the seed only control. This experiment was performed once.

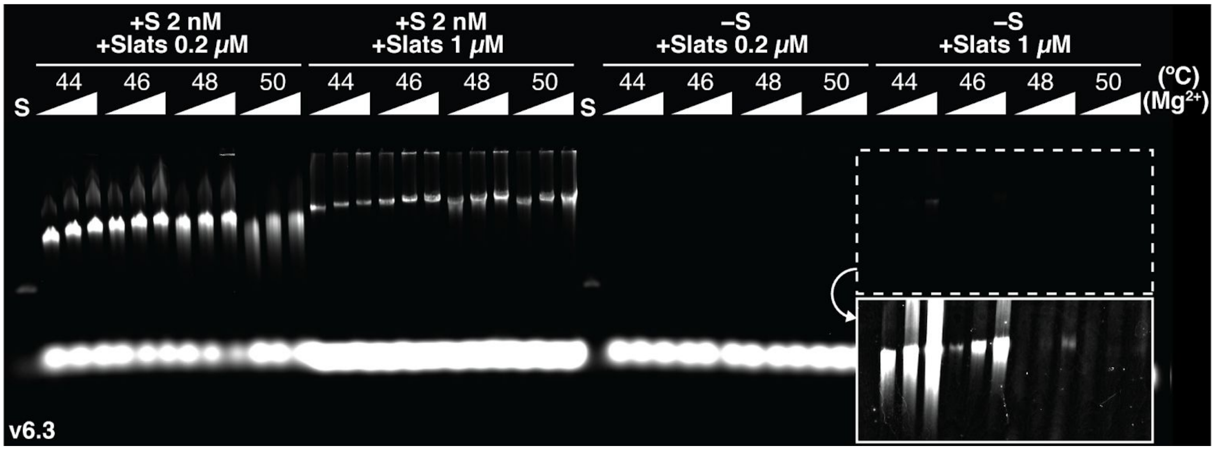

Supplementary Figure 25. Gel characterization of seeded (left) and unseeded (right) growth of DNA v6.3 slats. Reactions with either 0.2 or 1.0 µM each slat were incubated isothermally at one of four temperatures in either 12, 14, or 16 mM $MgCl_2$ for ~16 hours. Unseeded reactions yielded no observable assembly with 0.2 µM each slat. However, unseeded reactions with 1.0 µM each slat yielded observable assembly as shown in the contrast and brightness adjusted cutaway from the gel. (S) is the seed only control.

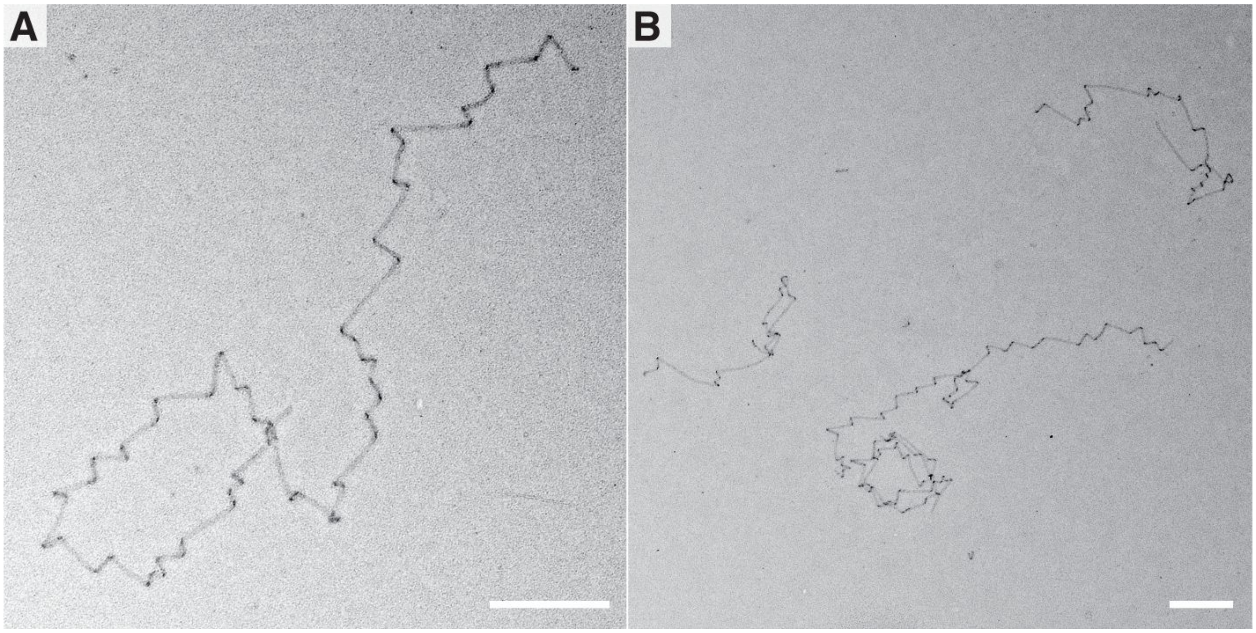

Supplementary Figure 26. **A** and **B**, TEM images of the spuriously formed gel band for v6.3 showing that the assemblies are well-formed ribbons, as opposed to some other artifact not matching the design. No seed was observed on any of the ribbons. The band from the gel in **Supplementary Figure 25** for one condition (−seed, 16 mM $MgCl_2$, 1 µM each slat, 44ºC) was excised with a razor blade, crushed with a pestle, and the aqueous solution that was forced out from the gel in the process was deposited onto a TEM grid and prepared as reported in the methods section. Scale bars are 500 nm.

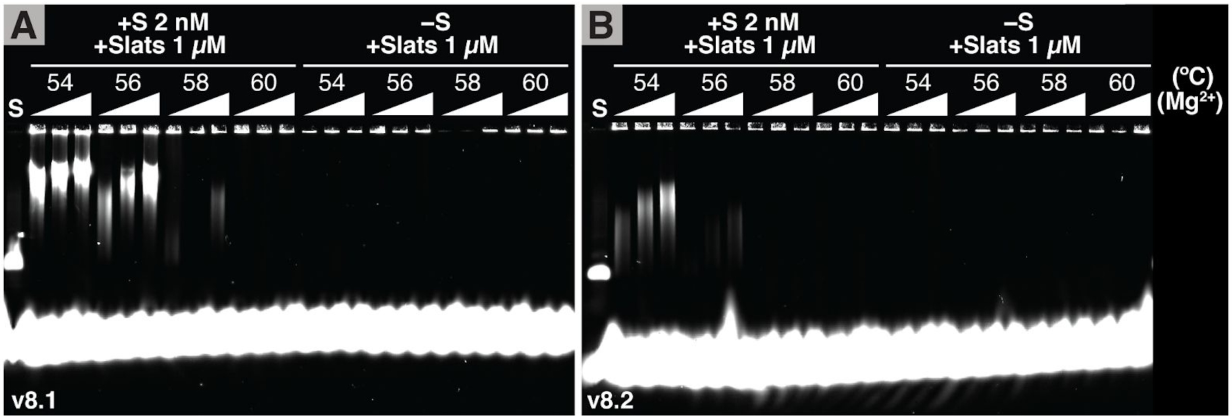

Supplementary Figure 27. Gel characterization of seeded and unseeded growth of DNA slats for v8.1 and v8.2 in **A** and **B** respectively. Reactions with 1 µM each slat were incubated isothermally at one of four temperatures in either 12, 14, or 16 mM $MgCl_2$ for ~16 hours. Reactions without a seed yielded no observable assembly at all conditions tested. (S) seed only control. Material stuck in the wells containing seeded ribbon growth presumably represents a mixture of aggregated ribbons and impurities, such as small lint pieces or dried agarose residue left on the gel comb. We hypothesize that DNA binds non-specifically to these small impurities resulting in a SYBR Gold signal from the wells. This gets especially hard to mitigate with reactions containing large amounts of DNA. To verify that the material stuck in the wells was not the result of aggregated ribbons that spuriously formed for DNA slats reactions without a seed present, we also viewed selected reactions on the TEM. This experiment was performed once.

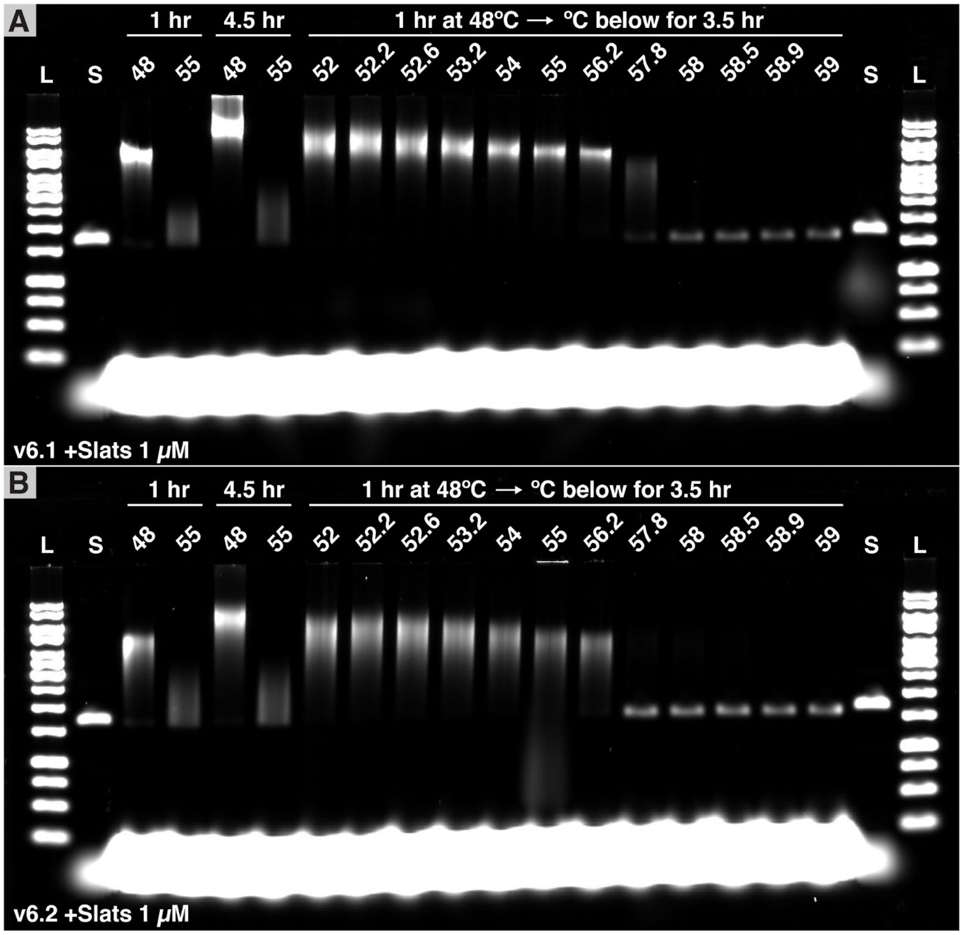

Supplementary Figure 28. We determined the approximate reversible temperature for slat binding for v6.1 and v6.2 by agarose gel. Ribbons were grown for 1 hour at 48ºC, and then the temperature was increased for 3.5 hours across a gradient ranging 52–59ºC to find the melt temperature where growth ends and ribbons fall apart. Gel bands for the ribbons were compared with respect to a control grown for 1 hour at 48ºC, where the temperature with the most similar position gel band to the control is the approximate reversible temperature. Additional controls at lower-optimal (48 ºC) and higher (55ºC) temperatures for the entire 4.5 hour duration of the experiment show how the rate of assembly is lessened as equilibrium conditions (i.e. close to the reversible temperature) are approached. **A–B**, Agarose gels showing the approximate reversible temperature of slat binding. All assemblies contained 16 mM $MgCl_2$ and 1 µM each slat. The 56.2ºC lane for both versions most closely resemble the gel position of the 1 hour 48ºC control, indicating that assembly from the initial 1 hour incubation was negligible. This suggests the reversible temperature is close to 56.2ºC for v6.1 and v6.2 at these reaction conditions. Furthermore, growth of ribbons was much more minimal when the entire 4.5 hour assembly was carried out near the reversible temperature (55ºC) versus much lower than the reversible temperature (48ºC). We highlight that the furthest-from-equilibrium 48ºC temperature did not show evidence of spurious ribbon formation for either v6.1 or v6.2 (see **Figure 3E** and **Supplementary Figure 24**).

Supplementary Figures 29–35: Ribbon assembly characteristics by experimental observation and modelling

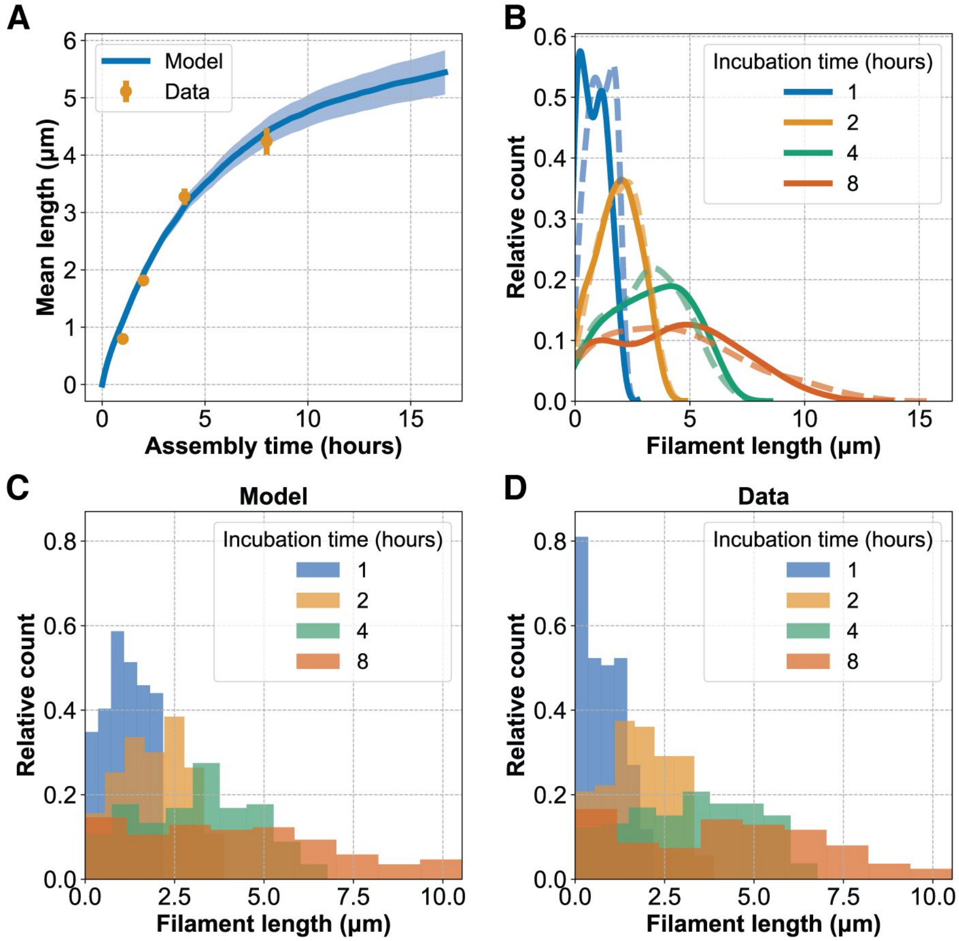

Supplementary Figure 29. Stochastic model fitting for v6.1 at experimental conditions of 50°C, 16 mM $MgCl_2$, and 1 μM each slat, considering only seed-initiated assembly. Estimated parameters are p_{stall} = 0.037 per minute, p_{term} = 0.0030 per minute, and l_{growth} = 31.37 nm/minute. **A**, Mean lengths of the distributions of model vs data. Error bars represent the standard error of the mean and are hidden by the markers for some of the points. **B**, Gaussian kernel density estimates for model (dashed line) vs data (solid line). **C–D**, Histograms of the unprocessed length distributions for the model and data respectively. Polydispersity indices for the data are 1.52, 1.27, 1.27 and 1.41 for 1, 2, 4 and 8 hour incubations respectively. N_{1h}=163, N_{2h}=130, N_{4h}=141, N_{8h}=140.

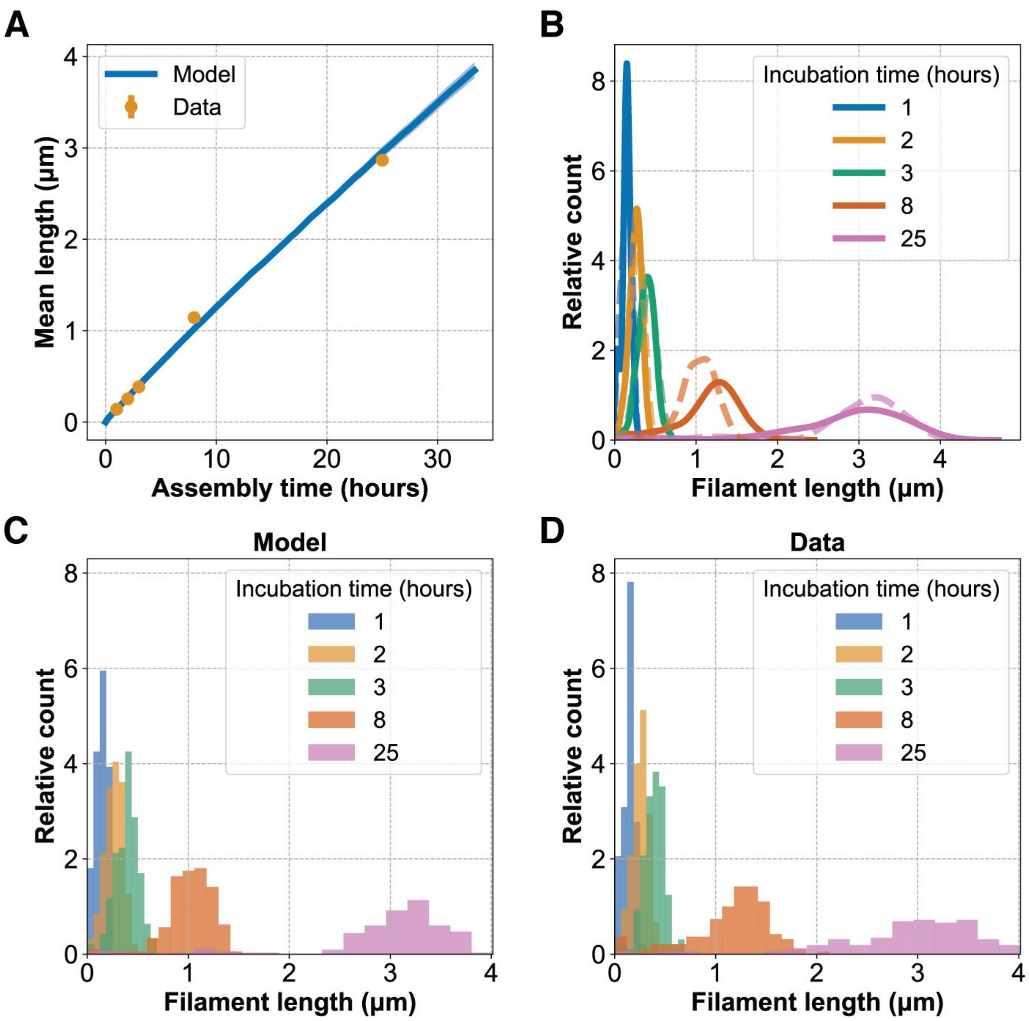

Supplementary Figure 30. Stochastic model fitting for v6.1 at experimental conditions of 40°C, 20 mM $MgCl_2$, and 1 µM each slat, considering only seed-initiated assembly. Estimated parameters are p_{stall} = 0.063 per minute, p_{term} = 0.000056 per minute, and l_{growth} = 4.12 nm/minute. **A**, Mean lengths of the distributions of model vs data. Error bars represent the standard error of the mean and are hidden by the markers for some of the points. **B**, Gaussian kernel density estimates for model (dashed line) vs data (solid line). **C–D**, Histograms of the unprocessed length distributions for the model and data respectively. Polydispersity indices for the data are 1.16, 1.09, 1.08, 1.13 and 1.07 for 1, 2, 3, 8 and 25 hour incubations respectively. N_{1h}=155, N_{2h}=168, N_{3h}=154, N_{8h}=161, N_{25h}=151.

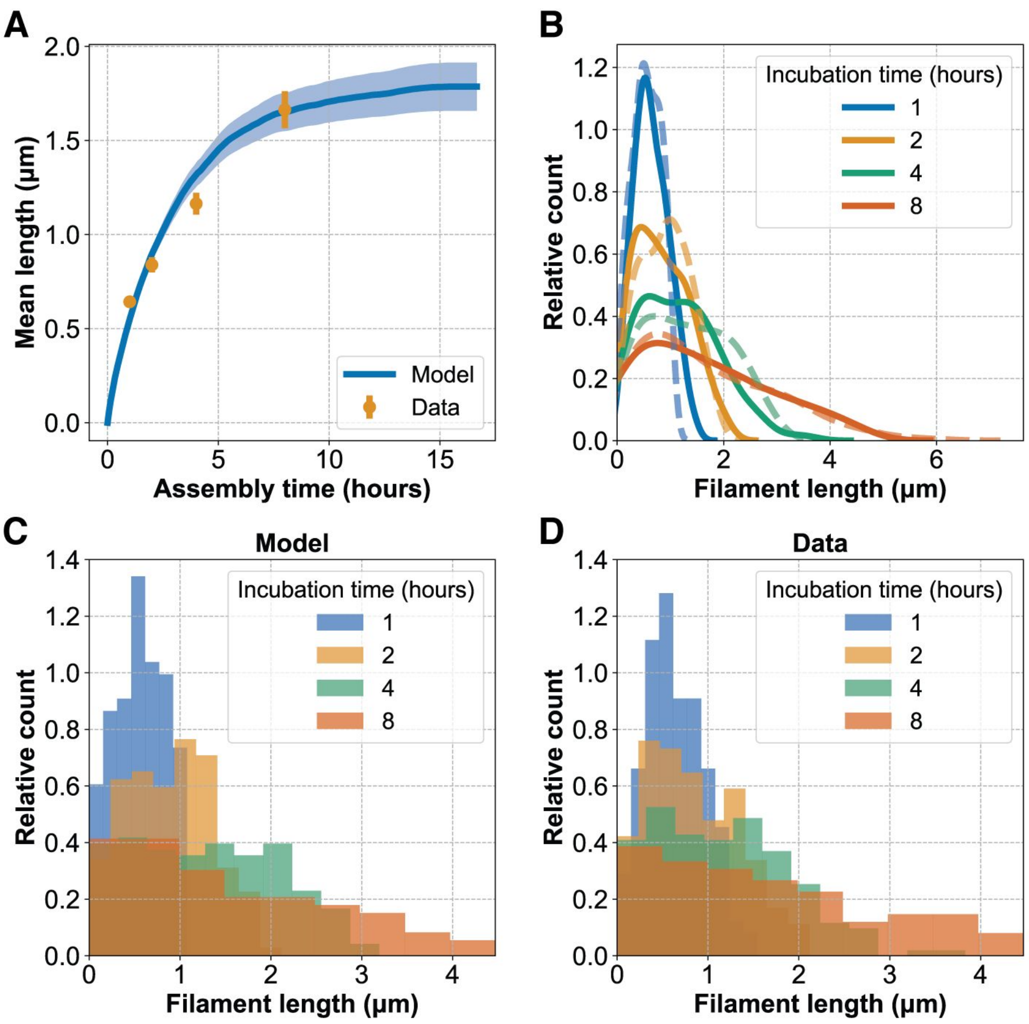

Supplementary Figure 31. Stochastic model fitting for v6.2 at experimental conditions of 50°C, 16 mM $MgCl_2$, and 1 μM each slat, considering only seed-initiated assembly. Estimated parameters are p_{stall} = 0.047 per minute, p_{term} = 0.0050 per minute, and l_{growth} = 8.53 nm/minute. **A**, Mean lengths of the distributions of model vs data. Error bars represent the standard error of the mean and are hidden by the markers for some of the points. **B**, Gaussian kernel density estimates for model (dashed line) vs data (solid line). **C–D**, Histograms of the unprocessed length distributions for the model and data respectively. Polydispersity indices for the data are 1.24, 1.35, 1.40 and 1.53 for 1, 2, 4 and 8 hour incubations respectively. N_{1h}=157, N_{2h}=151, N_{4h}=161, N_{8h}=151.

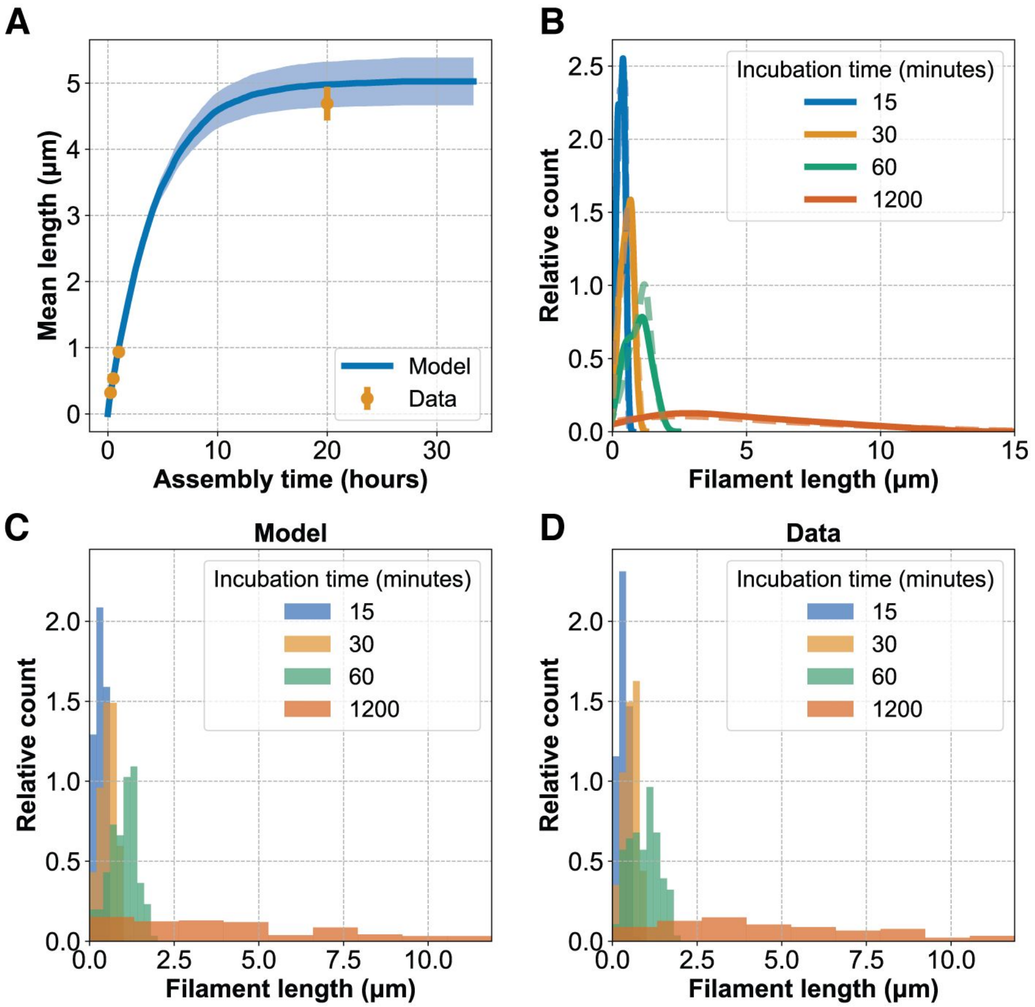

Supplementary Figure 32. Stochastic model fitting for v6.2 at experimental conditions of 50°C, 14 mM $MgCl_2$, and 1.5 μM each slat, considering only seed-initiated assembly. Estimated parameters are p_{stall} = 0.116 per minute, p_{term} = 0.0031 per minute, and l_{growth} = 31.84 nm/minute. **A**, Mean lengths of the distributions of model vs data. Error bars represent the standard error of the mean and are hidden by the markers for some of the points. **B**, Gaussian kernel density estimates for model (dashed line) vs data (solid line). **C–D**, Histograms of the unprocessed length distributions for the model and data respectively. Polydispersity indices for the data are 1.17, 1.16, 1.22 and 1.40 for 15, 30, 60 and 1200 minute incubations respectively. *Note:* the v6.2 DNA slats used in this experiment were double PAGE purified. N_{15min}=159, N_{30min}=113, N_{1h}=139, N_{20h}=137.

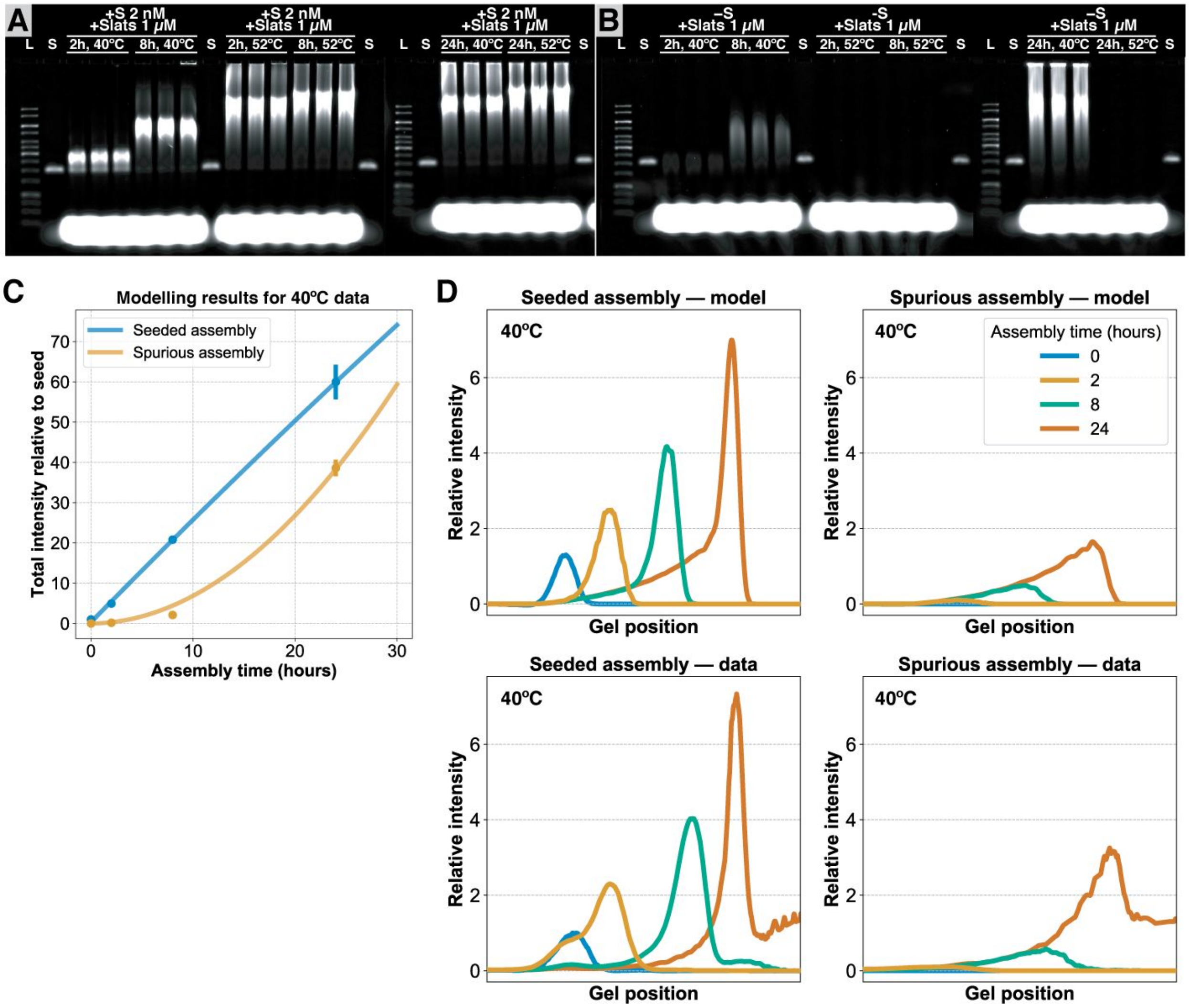

Supplementary Figure 33. Reproduction of gel intensity profiles using the analytical solution. **A–B**, Agarose gels for v6.1 at experimental conditions of 40 or 52°C, 20 mM $MgCl_2$, and 1 µM each slat for either with (gel **A**) or without (gel **B**) the seed. Spurious and seeded growth was seen at 40°C, versus no observable spurious ribbons at 52°C. Each experimental condition was performed in triplicate, with all replicates shown on the gel here. **C**, Analytical solution to the stochastic model (solid line) compared to the 40°C data from the gels in ***A–B*** using parameters estimated in the TEM data fit of **Supplementary Figure 30**. Error bars represent the standard error of the mean and are hidden by the markers for some of the points. Note that here seeded assembly is the sum of assembly from seed nucleation as well as spurious nucleation. **D**, Bottom row shows averaged gel intensity profiles for 40°C data from gels ***A–B***, and the top row shows gel intensity as predicted by the stochastic model using the parameters estimated in ***C***.

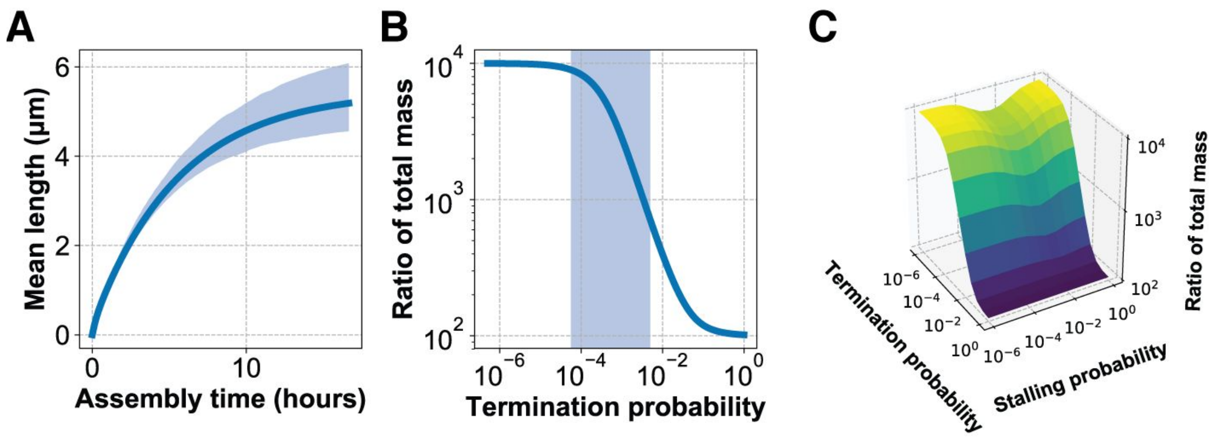

Supplementary Figure 34. Characterisation of the analytical solution. **A**, Mean length versus time derived from the analytical solution (solid line) compared to the 95% confidence interval of 500 runs of the stochastic model (shaded area), using the parameters from the fit in **Supplementary Figure 29**. **B–C**, Expected ratio of the total mass of spuriously nucleated ribbons for a 100-hour incubation compared to a 1-hour incubation (equivalent to total integrated intensity on a gel) based on the analytical solution, assuming no stalling in ***B*** and with variable stalling in ***C***. Shaded area in ***B*** corresponds to termination probabilities observed in **Supplementary Figures 29–32**.

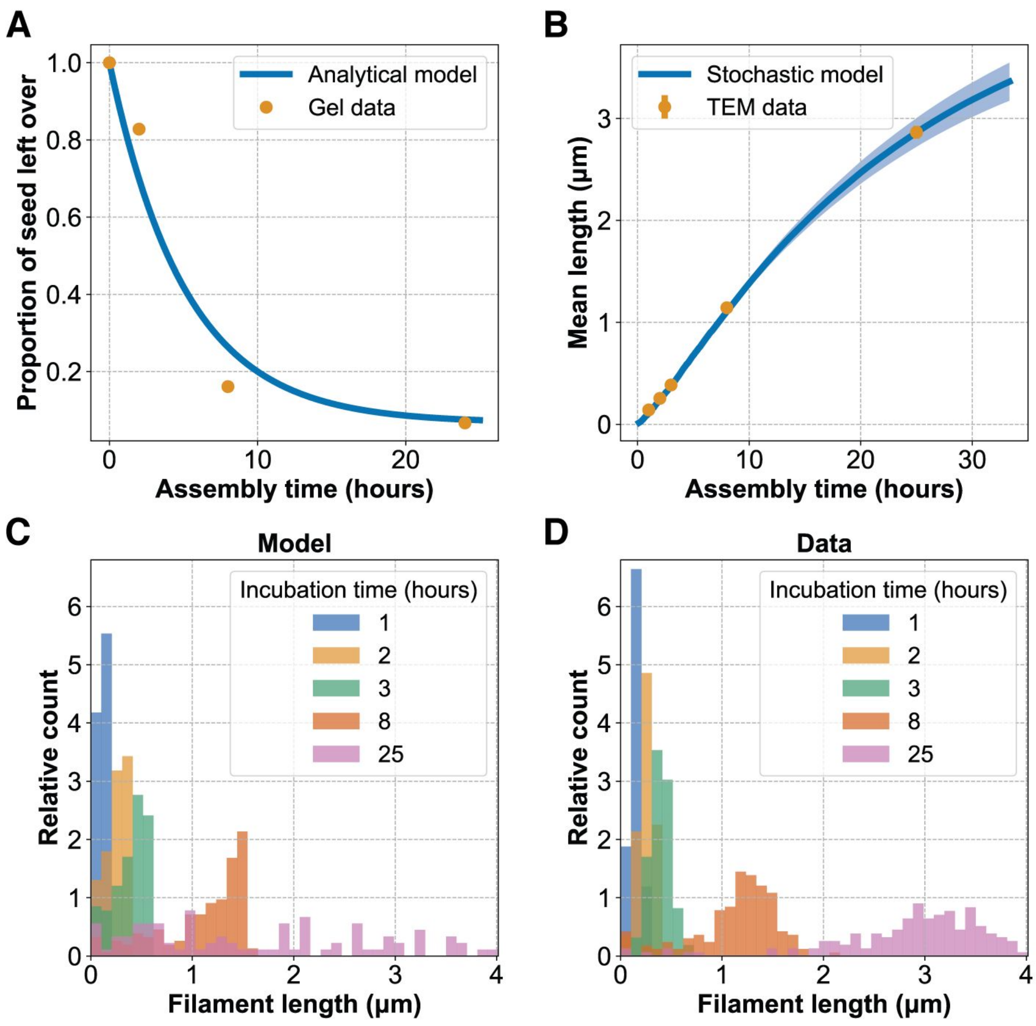

Supplementary Figure 35. Estimation of seed initiation delay and execution of model considering delayed seed initiation but not stalling. **A**, Single exponential decay corresponding to seed initiation at a rate of 0.0032 per minute compared to the seed peak height from averaged data in the gel from **Supplementary Figure 33**. **B−D**, Stochastic model fitting for v6.1 at experimental conditions of 40°C, 20 mM $MgCl_2$, and 1 μM each slat (as in **Supplementary Figure 30**). Estimated parameters are p_{seed} = 0.013 per minute, p_{term} = 0.00067 per minute, and l_{growth} = 3.22 nm/minute (p_{stall} taken as zero). **B**, Mean lengths of the distributions of model vs data. Error bars represent the standard error of the mean and are hidden by the markers for some of the points. **C–D**, Histograms of the unprocessed length distributions for the model and data respectively (using different histogram binning to **Supplementary Figure 30D** for a clearer comparison between model and data in each case). N_{1h}=155, N_{2h}=168, N_{3h}=154, N_{8h}=161, N_{25h}=151.

Supplementary Figures 36–39: Quantification of spontaneous nucleation under suboptimal low-temperature growth conditions

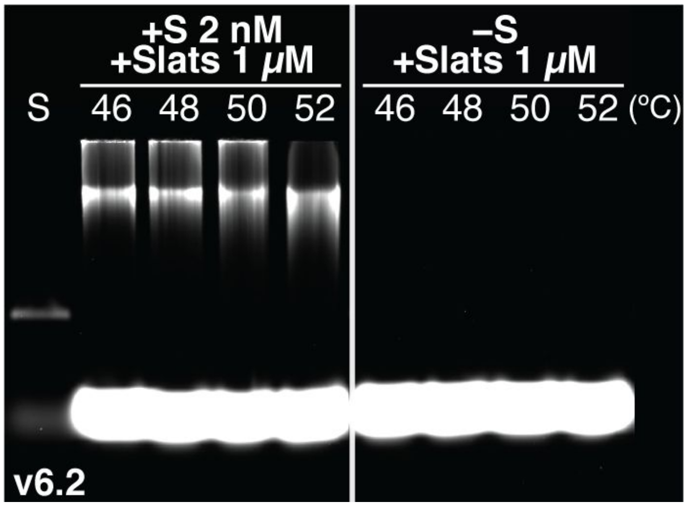

Supplementary Figure 36. Agarose gel showing extended 100 hour incubation of v6.2 slats. Reaction conditions were 16 mM $MgCl_2$ with 1 µM each slat, and 2 nM seed for the +S seeded control in the left half of the gel versus no seed in the right half of the gel. This experiment uses the same reaction conditions as shown for v6.1 in **Figure 3H**. By contrast, no spurious ribbons could be observed at any temperature for v6.2 when no seed was present. Qualitatively, v6.2 seems less prone to spontaneous nucleation compared to v6.1. The quantitative differences in nucleation behavior between these two versions is detailed more thoroughly in **Supplementary Figure 39**. This experiment was performed once.

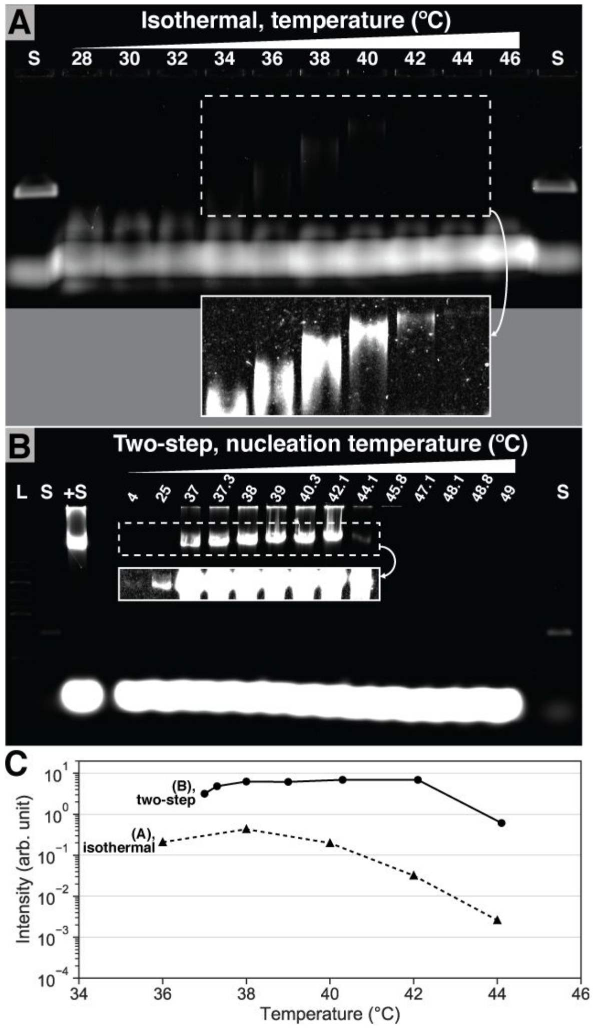

Supplementary Figure 37. Unseeded assembly of v6.1 at suboptimal lower slow-growth temperatures causes spontaneous growth of ribbons observable on agarose gels. **A**, Agarose gel showing no-seed reactions with isothermal incubation at the temperatures shown above each well (16 mM $MgCl_2$, 1 µM each slat, ~16 hours growth). Spuriously nucleated ribbons of various lengths can be observed for 34 to 44ºC, seen more clearly in the contrast and brightness adjusted inset. However, the molecular mass of ribbons became smaller as the temperature was lessened, suggesting that assembly was slower at lower temperatures. **B**, Agarose gel showing no-seed reactions incubated using the two-step isothermal incubation as detailed in the materials and methods. Contrast and brightness adjusted in the inset panel shows the fainter bands more clearly. In comparison to ***A***, the relative length of the spuriously assembled ribbons is similar at all temperature points. This suggests that two-step temperature incubation can be used to normalize lengths of nuclei formed at less favorable growth conditions so that relative nucleation can be compared between conditions. **C**, Plot of gel densitometry measurements, showing more assembly in ***B*** with two-step growth versus isothermal growth in ***A***. The observations in this figure suggest the following about slat behavior: (1) Spurious nucleation is slower as the lower bound of the optimal growth temperatures (i.e. increasing towards ~46ºC) is approached; (2) Formation of spurious nuclei are relatively high for some range of temperatures (e.g. 37–42.1ºC) below the growth optimum. However, assembly of these nuclei into ribbons is progressively slower as temperature is decreased; and (3), The slats at temperatures much lower (i.e. 4ºC and 25ºC) than the growth optimum form relatively few spurious assemblies. We hypothesize that this observed dropoff in growth rate at lower temperatures might be due to misbonding of the slats (i.e. kinetic traps). The effect of kinetic trapping of the slats versus the rate of spontaneous nucleation is not clear.

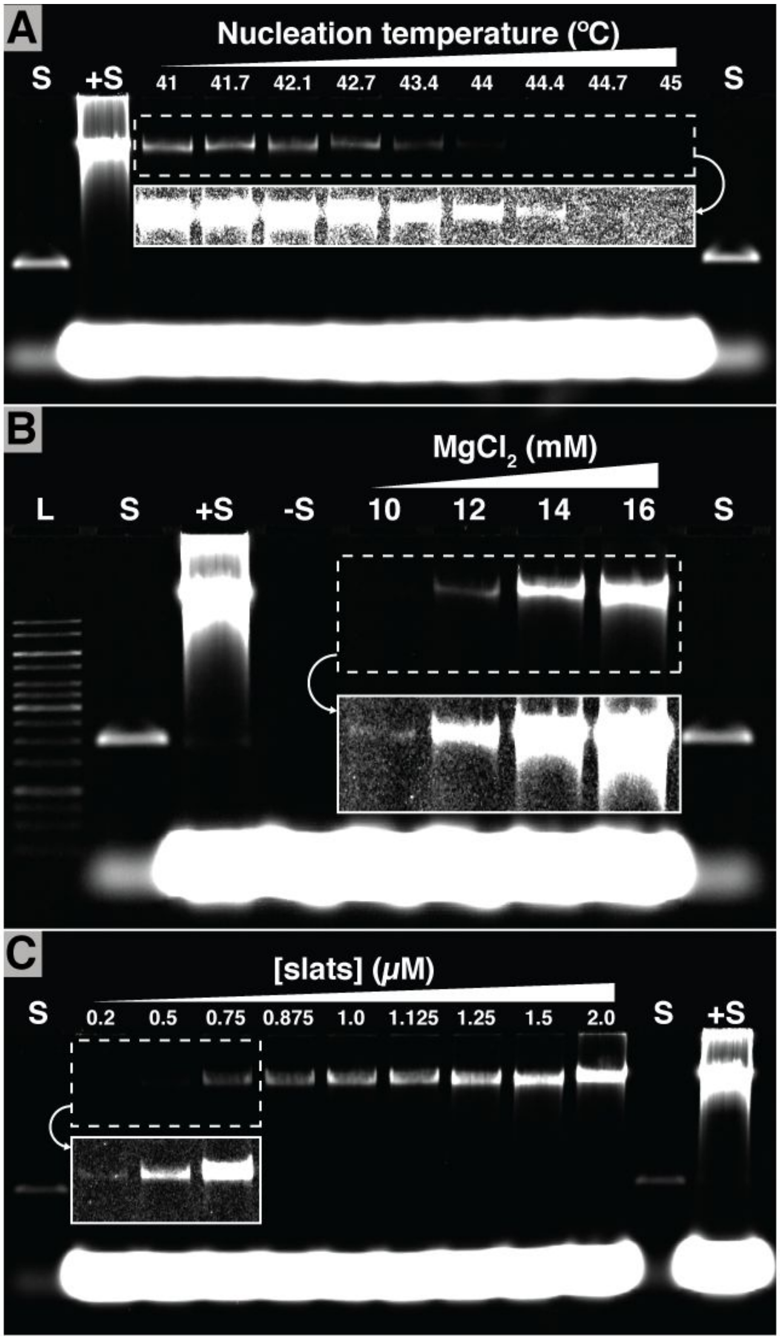

Supplementary Figure 38. Agarose gels of single experiments for which nucleation rates vs experimental parameters (temperature, $MgCl_2$ concentration, and concentration of each slat) in **Figure 3I** were determined. Gel lanes (+S) are control reactions where growth was initiated with 2 nM seed, (S) are seed normalization controls, and (L) is the ladder. Growth of filaments from spuriously formed nuclei were detailed using the two-step isothermal incubation discussed in the Materials and Methods and **Supplementary Figure 37.** Specific reaction condition parameters for each gel experiment are given in "*Two-step isothermal incubation of v6 slats to observe spontaneous nucleation and measure nucleation rate*" of the Materials and Methods. **A** and **B** were performed in triplicate, while **C** was performed in duplicate. The nucleation rate for a given condition was determined by taking the densitometry ratio with respect to the +S control that was grown with 2 nM seed for the latter growth phase of the two-step incubation. Inset sections of gels in white are contrast and brightness adjusted to better-show samples with lower amounts of ribbons.

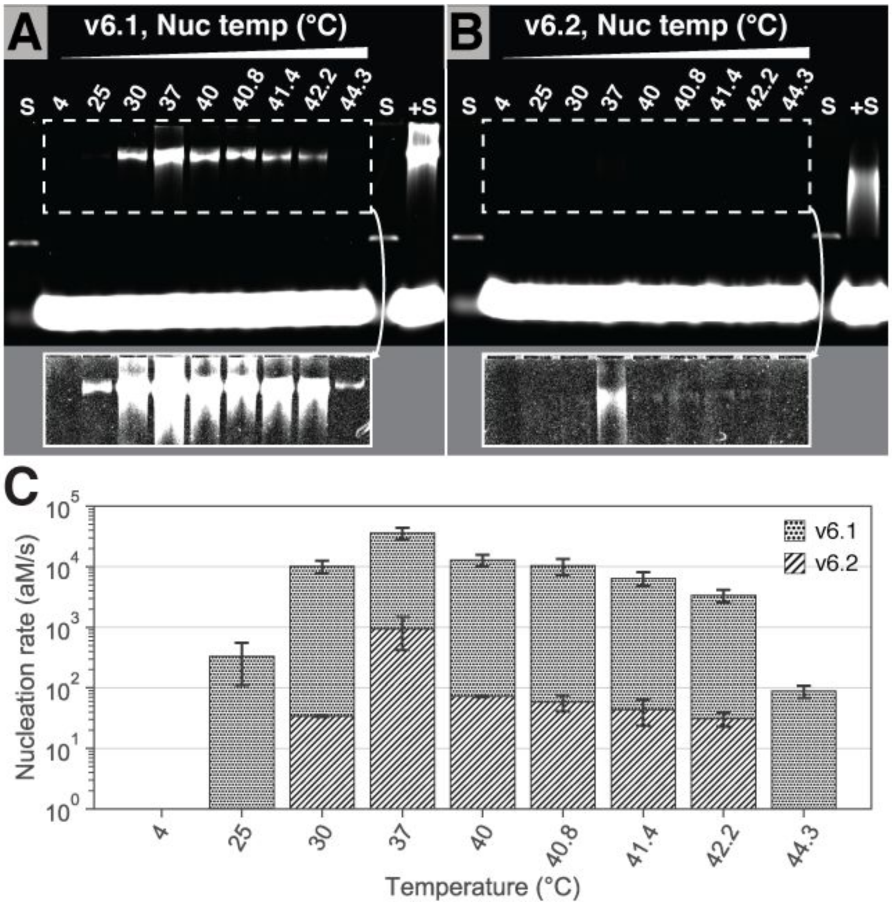

Supplementary Figure 39. Characterization of spurious nucleation for v6.1 and v6.2 at select temperature points. **A**, Single gel of nucleation of v6.1 slats. **B**, Single gel of nucleation of v6.2 slats. Inset sections of the gel in white are contrast and brightness adjusted to better show samples with lower concentrations of ribbons. Lane (S) is the seed only, and (+S) is the 2 nM seeded control that was grown only for the latter growth normalization step. Two-step experiments were carried out as detailed in the materials and methods. **C**, Plot of mean nucleation rates for v6.1 and v6.2 with respect to the +S control at nucleation temperatures, where bars are only shown for temperatures where a gel band could be measured above the gel detection limit. There are ~100-fold fewer spontaneous nuclei for v6.2 versus v6.1, with variations at different nucleation temperatures. All error bars are the ±SD for triplicate (v6.1) and duplicate (v6.2) experiments.

Supplementary Figure 40: Crisscross polymerization to sense nucleic acid sequences

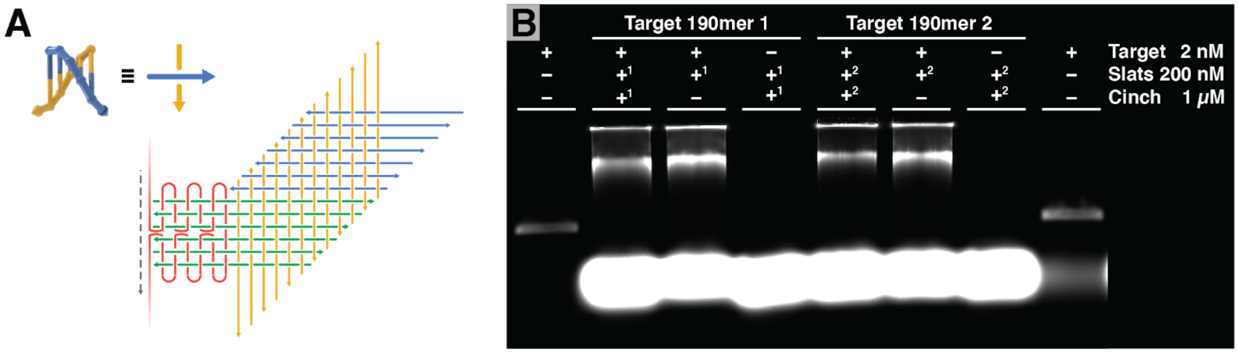

Supplementary Figure 40. We generalize the seed architecture from a DNA origami structure (**Supplementary Figure 13**) to a 190 nt region comprising six loops[1*] of the M13 p8064 scaffold. We designed two different sets of nuc-x-slats (6 different length slat sequences) that respectively are able to use two specific regions of 190 nt length from the p8064 scaffold as a seed and subsequently trigger DNA slat ribbon growth with v6.3 x- and y-slats (Note that in this figure x- and y-slat DNA sequences are swapped, i.e. x-slat DNA sequences act as y-slats). We show that nuc-x-slats are able to trigger the ribbon polymerization with or without a specially designed cinch strand. Most importantly, we show that without the target p8064 ssDNA sequence we see no spurious ribbon assembly—i.e. no signal—on the agarose gel. **A**, Abstract strand diagram depicting how nuc-x-slats (green) bind to a 190 nt short region of the p8064 scaffold target (red), to seed crisscross ribbon polymerization (gold y-slats and blue x-slats). The optional cinch strand is shown in dashed gray. **B**, Agarose gel showing crisscross polymerization seeded by two 190 nt long target regions of the M13 p8064 scaffold with and without a cinch strand. Superscripts on the + sign denote that certain strands in the pool (i.e. nuc-x slats or the cinch strand) were customized to target a specific region of the target. '$+^1$' denotes target region 1 of the p8064 scaffold, and '$+^2$' denotes target region 2 of the p8064 scaffold. In the future different sensors could be designed as pre-formed crisscross seeds that first are incubated with the analyte solution for target capture. Each sensor seed would be programmed to disintegrate during a subsequent timed destruction phase in the case that did not remain bound to the target for the full duration. Thus surviving seeds and subsequently polymerized ribbons would represent positive identifications. This experiment was performed once.

[1*] Xiao, S.-J. Oral presentation. *24th International Conference on DNA Computing and Molecular Programming* (2018). *Note: From his presentation in the same session as one of us (William Shih), it was apparent that Prof. Xiao had, independently from our group, discovered that single-stranded tiling could be achieved with y-tiles that crossover every half-turn. Also see Figure 4B of ref.* [30]*, for a further example of an array of half-duplex domains.*

Supplementary Figures 41–46: Twisted ribbons and tubes

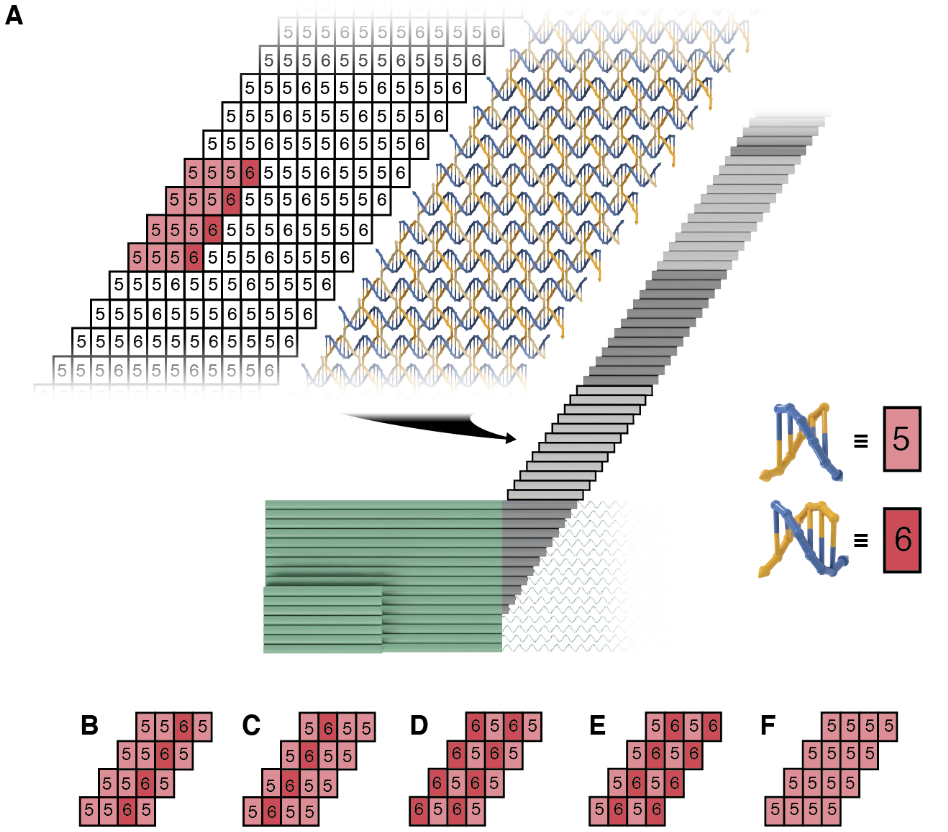

Supplementary Figure 41. A DNA slat consists of multiples of four consecutive binding domains (i.e. *n = 6; 3 × 4 = 12* binding domains ***or*** *n = 8; 4 × 4 = 16* binding domains) that are composed of 5 and/or 6 bp binding domains. Each four binding domain segment has three 5 bp and one 6 bp binding domain (totalling 21 bp or 10.5 bp/turn), two 5 bp and two 6 bp binding domains (totalling 22 bp or 11 bp/turn underwound DNA), or four 5 bp binding domains (i.e. 20 bp or 10 bp/turn overwound DNA). The order of the 5 or 6 bp binding domain arrangement within the four binding domain long segments gives rise to ribbons with different coiled morphologies. We tested a total of six different binding domain arrangements. **A**, Pattern of rectangles with corresponding binding domains of length 5 or 6 bp are displayed next to the 3D DNA ball and stick model. The pattern of four sequential binding domains constituting of 20, 21, or 22 bp per two helical turns is highlighted in red. The 5556 pattern is used in v6.1, v6.2, v6.3, v8.2, and v8.5, all with 10.5 bp/turn. **B**, Binding domain pattern in v8.1 with 10.5 bp/turn. **C**, Binding domain pattern in v8.4 with 10.5 bp/turn. **D**, Binding domain pattern in v8.6 with 11 bp/turn. **E**, Binding domain pattern in v8.3 with 11 bp/turn. **F**, Binding domain pattern in v8.7 with 10 bp/turn.

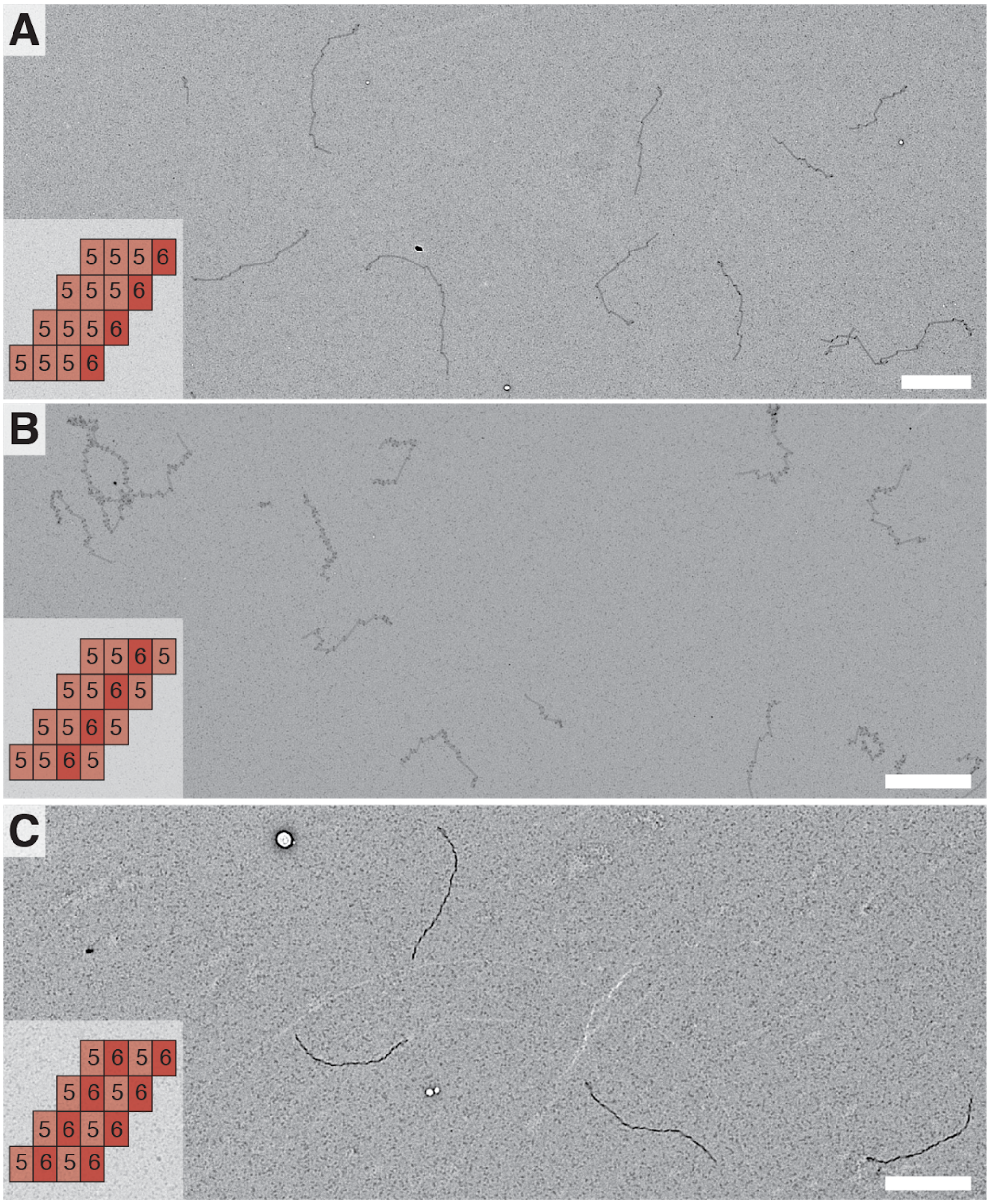

Supplementary Figure 42. Negative-stain TEM micrographs showing DNA slat variants v8.1, v8.2, and v8.3 without sticky ends. **A**, Binding domain pattern in v8.2. The "flat" ribbons grown exhibit minimal coiling. We hypothesize that v8.2 still has some inherent coiling in solution, which results in kinked ribbons once deposited onto a carbon grid for TEM imaging. However, the kinks are of a much lesser extent in this variant when compared to all of the other tested designs. **B**, Binding domain pattern in v8.1. This variant displayed loosely coiled ribbons on the TEM micrographs. **C**, Binding domain pattern in v8.3. This variant displayed tightly coiled ribbons on the TEM micrographs. Both variants shown in panels ***A*** and ***B*** were used to assemble tubes. Scale bars are 1 µm.

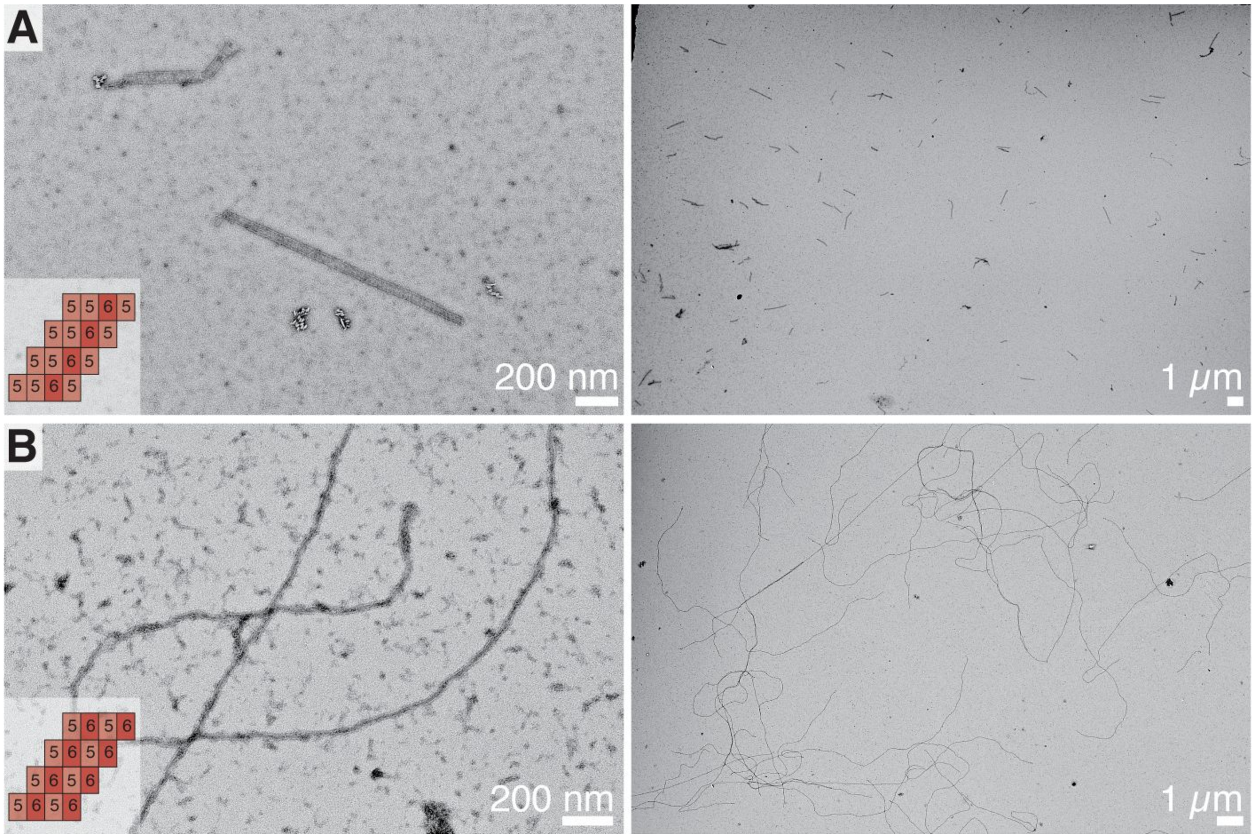

Supplementary Figure 43. Negative-stain TEM micrographs showing DNA slats v8.1 and v8.3 with 2 nt sticky ends along the edge of the ribbon. **A**, v8.1 forms thick tubes of varied diameters in a single reaction. We hypothesize that the loosely coiled nature of v8.1 combined with 2 nt sticky ends gives the ribbons the ability to close with one of many possible diameters. Once a specific diameter is initially realized at the start of the reaction, the ribbons follow this template to continue growing with a relatively constant diameter. However, we also observed tubes with slight diameter variations along the axial of the tube. **B**, By contrast, v8.3 forms thin tubes of more constant diameter. We hypothesize that the tightly coiled nature of v8.3 combined with 2 nt sticky ends, lessened the spread of diameters with which the ribbons could close into tubes.

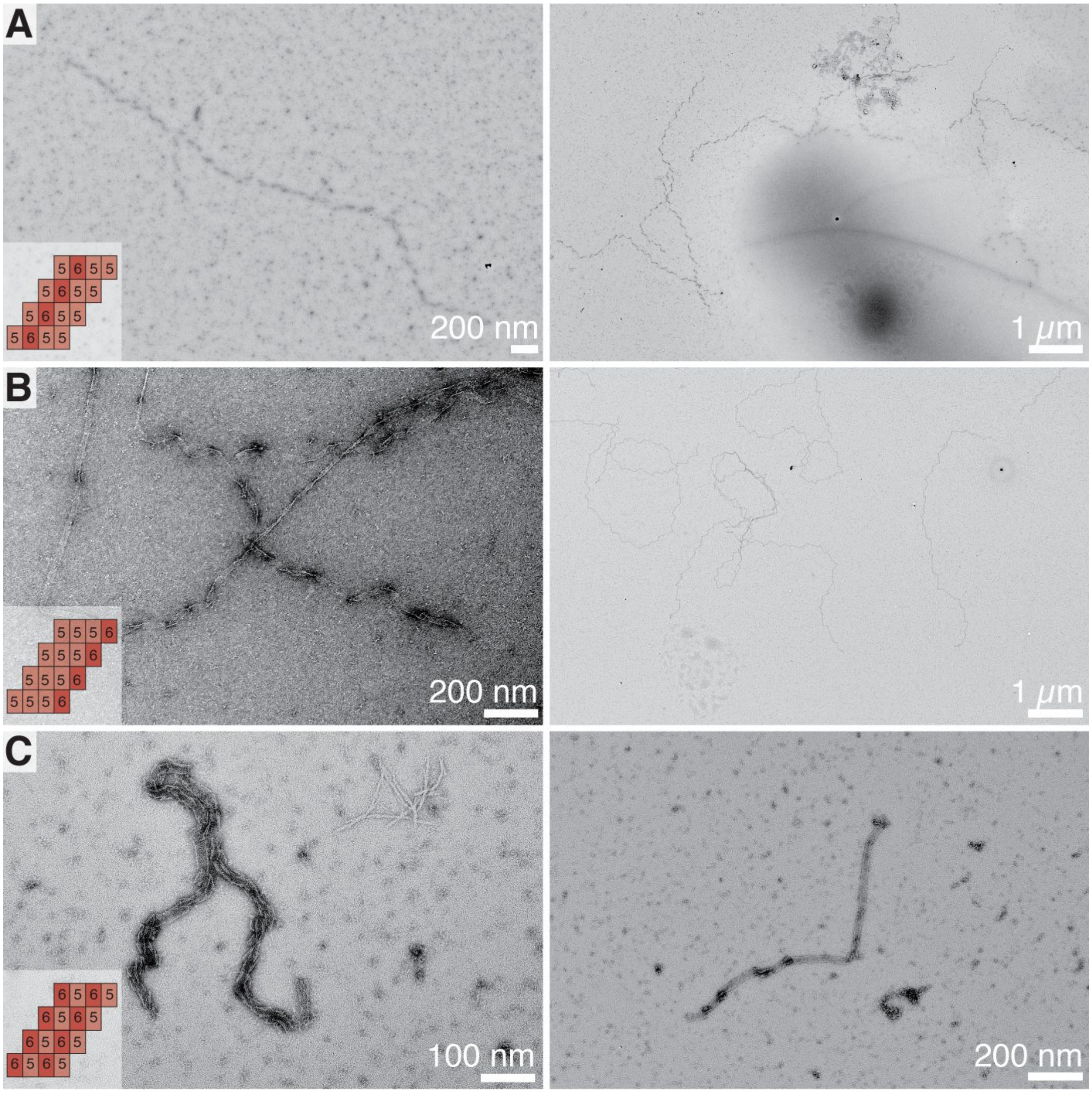

Supplementary Figure 44. Negative-stain TEM micrographs showing DNA slats v8.4, v8.5, and v8.6 in **A**, **B**, and **C** respectively with 2 nt sticky ends along the edge of the ribbon. Compared to v8.1 and v8.3 in **Supplementary Figure 43**, these "tubes" were all misformed. DNA slat v8.4, v8.5, and v8.6 were screened on the TEM once.

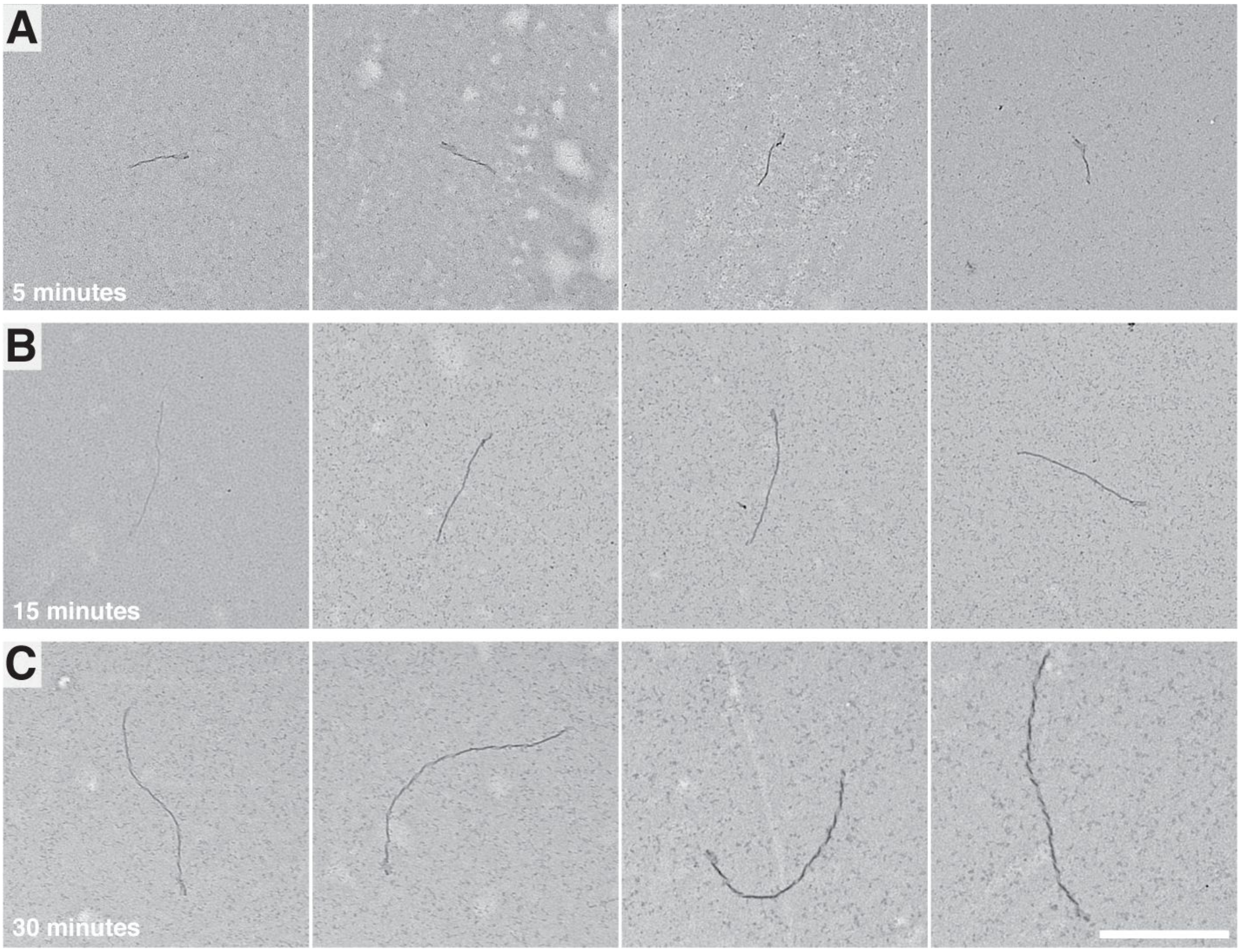

Supplementary Figure 45. Negative-stain TEM micrographs for sequence v8.3 without sticky ends. DNA slat at 0.5µM were assembled with 0.02 nM seed in 14 mM Mg^{2+} at 52ºC. **A**, 5 minutes of assembly yield 275 nm ± 44 nm in length. **B**, 15 minutes of assembly yield 770 nm ± 55 nm in length. **C**, 30 minutes of assembly yield 1460 nm ± 207 nm in length. We estimated the second-order rate constant to be approx. 10^6 $M^{-1}s^{-1}$. Mean ± SD for N=4 independent ribbon lengths, was computed. Scale bar is 800 nm.

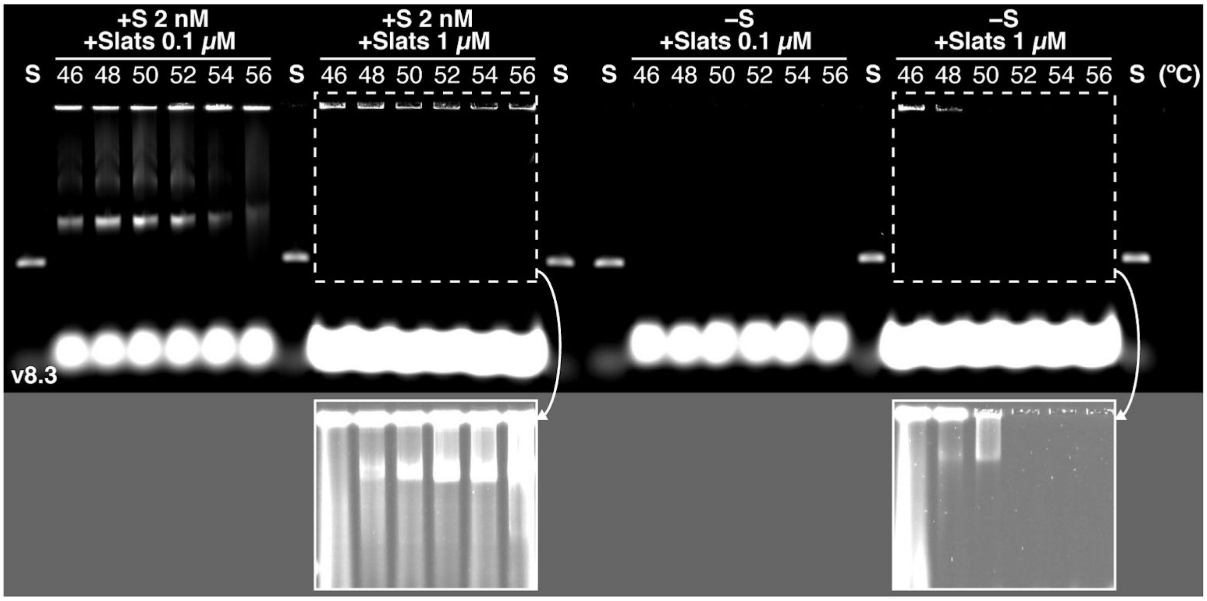

Supplementary Figure 46. Agarose gel characterization of seeded (left) and unseeded (right) growth of DNA v8.3 slats. Reactions with either 0.1 or 1.0 µM each slat were incubated isothermally at 46ºC–56ºC in 16 mM $MgCl_2$ for ~16 hours. Notably, unseeded reactions for 0.1 µM each slat yielded no observable assembly at all conditions tested. Unseeded reactions for 1 µM each slat yielded in spurious assembly (as shown in inserts where contrast and brightness were adjusted for better visualization) only for 50ºC and below. (S) is the seed only control. Optimal growth conditions were especially hard to determine for high (i.e. 1 µM each) slat concentrations, since the v8.3 ribbons grow so long (over the course of 16 hours) that they aggregate in the well of each lane. For 0.1 µM slat concentration we estimate the optimal growth condition to be between 52ºC–54ºC. The v8.3 seeded and unseeded temperature and slat concentration screen was performed once.

Supplementary Tables 1–5

square-tile (ST) nanotubes<br>e.g. $n = 2$, $k_{max} = 8$, $G_{mc} = 7.3$, $\varepsilon = 2$	example: spurious nucleation of ST nanotubes versus CS ribbons of the equivalent programmed width ($k_{max} = 8$) under the same rapid, irreversible growth conditions (1 μM each monomer, growth/shrinkage = 100)	crisscross-slat (CS) ribbons<br>e.g. $n = 8$, $k_{max} = 8$, $G_{mc} = 7.3$, $\varepsilon = 2$
no	non-nearest-neighbor contacts allowed?	yes
relatively small<br>2	n<br>monomer half coordination number<br>i.e. number of pairs of coordination sites per monomer	relatively large<br>8
relatively large<br>112×2/2 = 112	$B\varepsilon/n$ for $k = k_{max}$<br>excess credits from bonded coordination sites stabilizing compact assembly of width k_{max}	relatively small<br>64×2/8 = 16
relatively small<br>$k_{max}G_{mc} - B\varepsilon/n$ =<br>8×7.3 – 112 = –53.6	$G(A) = (nN - B)(G_{mc}/n) - B(\varepsilon/n)$<br>free energy for compact assembly of width k_{max}	relatively large<br>$k_{max}G_{mc} - B\varepsilon/n$ =<br>8×7.3 – 16 = 42.4
ε < transitional ε: $k^* = k_{max}$<br>ε > transitional ε:<br>$k^* = (G_{mc} + \varepsilon)/2\varepsilon$<br>(7.3 + 2)/4 = 2.33	k^*<br>width of critical nucleus	$k^* = k_{max}/(1 + \varepsilon/G_{mc})$<br>8/(1 + 2/7.3) = 6.28
relatively large for an assembly of width k*<br>for $k^* = 2.33$,<br>6.2×1 = 6.2	$B\varepsilon/n$ for $k = k^*$<br>excess credits from bonded coordination sites stabilizing compact assembly of width k^*	relatively small for an assembly of width k*<br>for $k^* = 6.28$,<br>39.4×0.25 = 9.85
relatively small<br>$k^*G_{mc} - B\varepsilon/n$ =<br>2.33×7.3 – 6.2 = 10.8	$G(A) = (nN - B)(G_{mc}/n) - (B)(\varepsilon/n)$<br>free energy for compact assembly A of width k^*	k^*G_{mc} = 6.28×7.3 = 45.8<br>relatively large<br>61.0×0.913 – 9.85 = 45.8

Supplementary Table 1. Comparison of spurious nucleation for ST nanotubes versus CS ribbons. This table illustrates how non-nearest neighbor contacts lead to a large monomer coordination number and therefore smaller stabilizing excess credits (i.e. that lower the nucleation barrier) from bonded coordination sites in the critical nucleus. Shown in red is an example case of fast, irreversible growth corresponding to 1 μM each monomer and a ratio of rates of growth to shrinkage of 100, where $k_{max} = 8$ for both ST nanotubes and CS ribbons.

DNA slat sequence variant	Stronger base-stacking in y- over x-direction?
v6.1	Yes
v6.2	No
v6.3	Yes
v8.1	Yes
v8.2	No

Supplementary Table 2. DNA slat sequence variants designed to have stronger base-stacking in the y-over x-direction. DNA slat sequences for v6 and v8 were designed using custom Python scripts. We assessed base-pairing energies using Unafold[32] and designed all sequences to have minimal self-structure. We further designed DNA slat sequences to have stronger base-stacking in the y-direction than in the x-direction (using Unafold[32] or base-stacking energies reported in Protozanova et al.[33]), which resulted in more uniform length distributions of assembled ribbons, coupled with greater spurious nucleation (see **Supplementary Figures 13–21** for experimental results). This behavior was puzzling to us, as we initially hypothesized that stronger base-stacking in the x-direction than in the y-direction would give rise to more robust growth, and yet we observed the opposite trend. Currently we are unable to rationalize the basis for these trends. It is noteworthy that we were not able to resolve helical directionality using our negative-stain TEM imaging conditions; this difficulty in discriminating individual helices may arise from the dense crossover patterns within the ribbons holding those helices very close together. As a consequence, we were unable to resolve whether the stacking direction has the ability to switch spontaneously along the length of ribbons or whether the intended design of stronger base-stacking in y- over x-direction actually gave rise to a preferred stacking direction in the assembled ribbons. In the future, this should be resolvable by designing a contrast-heavy marker feature into the ribbon structure that clearly reports on the directionality of the helices in negative-stain TEM. Looking to the future, we believe that tuning base-stacking energies is one of potentially many parameters that can be optimized for DNA slat sequences. However, due to time and cost limitations we were unable to explore further the large DNA slat sequence design space in this initial study.

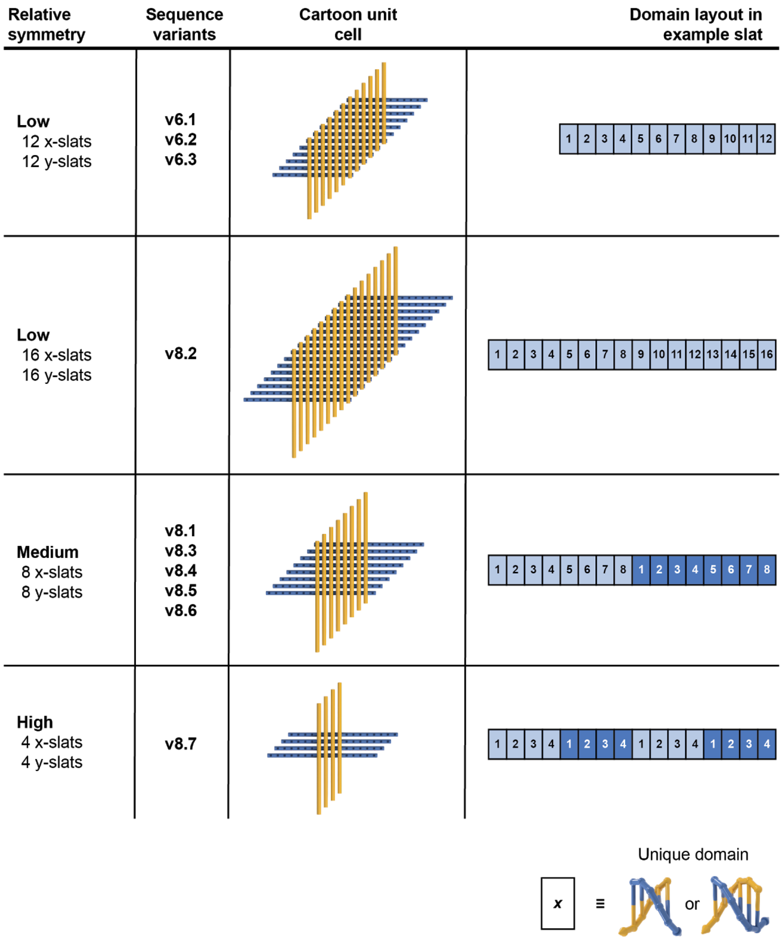

Supplementary Table 3. Sequence symmetry of specific variants for v6 and v8 designs. Increasing the sequence symmetry allows growth to be attained with fewer unique x- and y-slats. The concept of symmetry in slat design is illustrated in the rightward domain layout diagram. Each boxed cell is a unique half-turn binding sequence that is programmed to bind a specific perpendicular slat. The numbering '*x*' in each box refers to some specific complementary slat in the opposing orientation. Growth of ribbons is perpetuated by sequential binding of repeating sets of x and y-slats, as introduced in **Figure 2**. The domains (i.e. ssDNA sequences) overhanging from slats at the terminus of a growing ribbon determine the specific slats that bind next. The number of repeating slats in the set for a given variant is arbitrary and programmed into the sequence design of the slats themselves. We exploited sequence symmetry in certain variants so that ribbon growth was attainable with fewer unique x- and y-slats. We were interested in decreasing the number of unique slats per design because purchasing fewer slats per design tested made it more economically feasible to test a larger number of designs. Symmetry, in context of a given x- or y-slat, refers to the number of copies of a specific perpendicular slat to which it is bound in the final ribbon. The "low" sequence symmetries for v6 and v8 had 12 and 16 unique pairs of x- and y-slats, respectively. For v8, we also tested "medium" and "high" sequence symmetry so that that ribbon growth could be attained with 8 and 4 unique pairs of x- and y-slats, respectively.

Assembly time [s]	Mean length measured [nm]	SD of lengths [nm]	Mean # of slat additions*	SD # of slat additions*	# slat additions assuming $\tau = 2$ and $k = 10^6 M^{-1} s^{-1}$
300	275	44	183.33	29.33	150
900	770	55	513.33	36.67	450
1800	1460	207	973.33	138	900

* We are assuming 1.5 nm length increase per single slat addition.

Supplementary Table 4. Length measurements from **Supplementary Figure 44** used to approximate the second-order rate constant of v8.3 DNA slat assembly. We estimate the second-order rate constant with: $\tau = t_{1/2}/ln(2) = 1/(kC_0)$, where τ is the mean dwell time, $t_{1/2}$ is the half-life, C_0 the initial DNA slats concentration, and k is the second-order rate constant. If we assume $k = 10^6 M^{-1} s^{-1}$ and set $C_0 = 0.5 \times 10^{-6} M$ (as per experimental results shown in **Supplementary Figure 44**), $\tau = 1/(10^6 M^{-1} s^{-1} \times 0.5 \times 10^{-6} M) = 2\ s$. Thus, with the assumption of 1 slat addition every 2 seconds we get a close approximation of the $10^6 M^{-1} s^{-1}$ second-order rate constant.

DNA slat sequence variant	Morphology of assembled slat structure	Location/length of the poly-T brushes and method of addition
v6.1	flat ribbon	● nuc-y-slats 0–11: no brushes<br>● x-slats 0–11: 8T on 5′ and 3′-ends<br>● y-slats 0–11: no brushes<br>● Sequence entirely purchased from IDT with no modification
v6.2	flat ribbon	● nuc-y-slats 0–11: no brushes<br>● x-slats 0–11: 8T on 5′ and 3′- ends<br>● y-slats 0–11: no brushes<br>● Sequence entirely purchased from IDT with no modification
v6.3	flat ribbon	● nuc-y-slats 0–11: 8T on 5′-ends<br>● x-slats 0–11: 8T on 5′-ends<br>● y-slats 0–11: 8T on 5′-ends<br>● Sequence entirely purchased from IDT with no modification
v8.1	twisted ribbon	● nuc-y-slats 0–15: 6T on 5′-ends<br>● x-slats 0–7: 6T on 5′-ends and TdT addition to 3′-ends<br>● y-slats 0–7: 6T on 5′-ends and TdT addition to 3′-ends<br>● 5′-end as purchased from IDT, 3′-ends via TdT addition
v8.2	flat ribbon	● nuc-y-slats 0–15: no brushes<br>● x-slats 0–15: 6T on 5′-ends and TdT addition to 3′-ends<br>● y-slats 0–15: 6T on 5′-ends and TdT addition to 3′-ends<br>● 5′-end as purchased from IDT, 3′-ends via TdT addition
v8.7	twisted ribbon	● No brushes added to any of the slats

Supplementary Table 5. Poly-T brush passivation details for specific slat designs. These modifications were necessary to prevent aggregation of the flat and twisted ribbons. The enzymatic TdT addition of poly-T brushes is detailed in the Materials and Materials *"Enzymatic addition of poly-T brushes for v8.1 and v8.2 on 3′-ends of slats."*

Supplementary References

1. Zhao, D. & Moore, J. S. Nucleation–elongation: a mechanism for cooperative supramolecular polymerization. *Organic & biomolecular chemistry* **1**, 3471–3491 (2003).
2. De Greef, T. F. A. *et al.* Supramolecular Polymerization. *Chem. Rev.* **109**, 5687–5754 (2009).
3. Huang, Z. *et al.* Supramolecular Polymerization from Controllable Fabrication to Living Polymerization. *Macromol. Rapid Commun.* **38**, 1700312 (2017).
4. Adelizzi, B., Van Zee, N. J., de Windt, L. N., Palmans, A. R. & Meijer, E. W. Future of supramolecular copolymers unveiled by reflecting on covalent copolymerization. *Journal of the American Chemical Society* **141**, 6110–6121 (2019).
5. Wehner, M. & Würthner, F. Supramolecular polymerization through kinetic pathway control and living chain growth. *Nat Rev Chem* **4**, 38–53 (2020).
6. Hartlieb, M., Mansfield, E. D. H. & Perrier, S. A guide to supramolecular polymerizations. *Polym. Chem.* **11**, 1083–1110 (2020).
7. Jacobs, W. M., Reinhardt, A. & Frenkel, D. Rational design of self-assembly pathways for complex multicomponent structures. *Proc Natl Acad Sci USA* **112**, 6313–6318 (2015).
8. Reinhardt, A., Ho, C. P. & Frenkel, D. Effects of co-ordination number on the nucleation behaviour in many-component self-assembly. *Faraday Discuss.* **186**, 215–228 (2016).
9. Ogi, S., Sugiyasu, K., Manna, S., Samitsu, S. & Takeuchi, M. Living supramolecular polymerization realized through a biomimetic approach. *Nature Chem* **6**, 188–195 (2014).
10. Ogi, S., Stepanenko, V., Sugiyasu, K., Takeuchi, M. & Würthner, F. Mechanism of Self-Assembly Process and Seeded Supramolecular Polymerization of Perylene Bisimide Organogelator. *J. Am. Chem. Soc.* **137**, 3300–3307 (2015).
11. Kang, J. *et al.* A rational strategy for the realization of chain-growth supramolecular polymerization. *Science* **347**, 646–651 (2015).
12. Dirks, R. M. & Pierce, N. A. Triggered amplification by hybridization chain reaction. *Proceedings of the National Academy of Sciences* **101**, 15275–15278 (2004).
13. Yin, P., Choi, H. M. T., Calvert, C. R. & Pierce, N. A. Programming biomolecular self-assembly pathways. *Nature* **451**, 318–322 (2008).
14. Bi, S., Yue, S. & Zhang, S. Hybridization chain reaction: a versatile molecular tool for biosensing, bioimaging, and biomedicine. *Chem. Soc. Rev.* **46**, 4281–4298 (2017).
15. Lanier, L. A. & Bermudez, H. Living Supramolecular Polymerization of DNA. *Macromol. Rapid*

Commun. **39**, 1800342 (2018).
16. Ang, Y. S. & Yung, L.-Y. L. Rational design of hybridization chain reaction monomers for robust signal amplification. *Chemical communications* **52**, 4219–4222 (2016).
17. Liu, J. *et al.* Applications of Catalytic Hairpin Assembly Reaction in Biosensing. *Small* **15**, 1902989 (2019).
18. Yang, H. *et al.* In situ hybridization chain reaction mediated ultrasensitive enzyme-free and conjugation-free electrochemical genosensor for BRCA-1 gene in complex matrices. *Biosensors and Bioelectronics* **80**, 450–455 (2016).
19. Miao, P., Tang, Y. & Yin, J. MicroRNA detection based on analyte triggered nanoparticle localization on a tetrahedral DNA modified electrode followed by hybridization chain reaction dual amplification. *Chemical Communications* **51**, 15629–15632 (2015).
20. Wang, B., Thachuk, C., Ellington, A. D., Winfree, E. & Soloveichik, D. Effective design principles for leakless strand displacement systems. *Proceedings of the National Academy of Sciences* **115**, E12182–E12191 (2018).
21. Winfree, E. Algorithmic self-assembly of DNA. (California Institute of Technology, 1998).
22. Schulman, R. & Winfree, E. Programmable Control of Nucleation for Algorithmic Self-Assembly. *SIAM J. Comput.* **39**, 1581–1616 (2010).
23. Evans, C. G. & Winfree, E. Physical principles for DNA tile self-assembly. *Chemical Society Reviews* **46**, 3808–3829 (2017).
24. Barish, R. D., Schulman, R., Rothemund, P. W. & Winfree, E. An information-bearing seed for nucleating algorithmic self-assembly. *Proceedings of the National Academy of Sciences* **106**, 6054–6059 (2009).
25. Schulman, R. & Winfree, E. Synthesis of crystals with a programmable kinetic barrier to nucleation. *Proceedings of the National Academy of Sciences* **104**, 15236–15241 (2007).
26. Woods, D. *et al.* Diverse and robust molecular algorithms using reprogrammable DNA self-assembly. *Nature* **567**, 366 (2019).
27. SantaLucia Jr, J. & Hicks, D. The thermodynamics of DNA structural motifs. *Annu. Rev. Biophys. Biomol. Struct.* **33**, 415–440 (2004).
28. Gilroy, J. B. *et al.* Monodisperse cylindrical micelles by crystallization-driven living self-assembly. *Nature Chem* **2**, 566–570 (2010).
29. Rothemund, P. W. K. *et al.* Design and Characterization of Programmable DNA Nanotubes. *J. Am. Chem. Soc.* **126**, 16344–16352 (2004).
30. Bai, X., Martin, T. G., Scheres, S. H. & Dietz, H. Cryo-EM structure of a 3D DNA-origami object.

Proceedings of the National Academy of Sciences **109**, 20012–20017 (2012).

31. Douglas, S. M. *et al.* Rapid prototyping of 3D DNA-origami shapes with caDNAno. *Nucleic acids research* **37**, 5001–5006 (2009).

32. Markham, N. R. & Zuker, M. UNAFold. in *Bioinformatics* 3–31 (Springer, 2008).

33. Protozanova, E., Yakovchuk, P. & Frank-Kamenetskii, M. D. Stacked–unstacked equilibrium at the nick site of DNA. *Journal of molecular biology* **342**, 775–785 (2004).
