# Supplementary material for: Robust nucleation control via crisscross polymerization of highly coordinated DNA slats
Source: Nat Commun. 2021 Mar 19;12:1741. doi: 10.1038/s41467-021-21755-7 (PMC7979912; doi:10.1038/s41467-021-21755-7)
Supplement: Supplementary file 2 — Description of Additional Supplementary Files [file 41467_2021_21755_MOESM2_ESM.docx]

**Description of Additional Supplementary Files**

File Name: Supplementary Data 1

Description: Sequence information for DNA origami seed staples, DNA slats v6 and v8 variants, M13 p8064 scaffold, and additional sequences used in Supplementary Fig. 40 and 22.
