# Supplementary material for: Robust nucleation control via crisscross polymerization of highly coordinated DNA slats
Source: Nat Commun. 2021 Mar 19;12:1741. doi: 10.1038/s41467-021-21755-7 (PMC7979912; doi:10.1038/s41467-021-21755-7)
Supplement: Supplementary file 4 — Reporting Summary [file 41467_2021_21755_MOESM4_ESM.pdf]

nature research

Corresponding author(s): William Shih

Last updated by author(s): Jan 12, 2021

Reporting Summary

Nature Research wishes to improve the reproducibility of the work that we publish. This form provides structure for consistency and transparency in reporting. For further information on Nature Research policies, see our Editorial Policies and the Editorial Policy Checklist.

Statistics

For all statistical analyses, confirm that the following items are present in the figure legend, table legend, main text, or Methods section.

n/a	Confirmed	
☐	☒	The exact sample size (n) for each experimental group/condition, given as a discrete number and unit of measurement
☐	☒	A statement on whether measurements were taken from distinct samples or whether the same sample was measured repeatedly
☒	☐	The statistical test(s) used AND whether they are one- or two-sided<br>*Only common tests should be described solely by name; describe more complex techniques in the Methods section.*
☐	☒	A description of all covariates tested
☒	☐	A description of any assumptions or corrections, such as tests of normality and adjustment for multiple comparisons
☐	☒	A full description of the statistical parameters including central tendency (e.g. means) or other basic estimates (e.g. regression coefficient) AND variation (e.g. standard deviation) or associated estimates of uncertainty (e.g. confidence intervals)
☒	☐	For null hypothesis testing, the test statistic (e.g. F, t, r) with confidence intervals, effect sizes, degrees of freedom and P value noted<br>*Give P values as exact values whenever suitable.*
☒	☐	For Bayesian analysis, information on the choice of priors and Markov chain Monte Carlo settings
☒	☐	For hierarchical and complex designs, identification of the appropriate level for tests and full reporting of outcomes
☒	☐	Estimates of effect sizes (e.g. Cohen's d, Pearson's r), indicating how they were calculated

Our web collection on statistics for biologists contains articles on many of the points above.

Software and code

Policy information about availability of computer code

Data collection	AMT Image Capture Engine Software Version 7.0.0.255, Typhoon FLA 9500 Control Software Version 1.1 Build 1.1.0.187
Data analysis	Adobe Illustrator 2019, Unafold (Version 3.8), ImageJ (v2.0.0-rc-69/1.52i), Python 3 (code was written in either Spyder or Jupyter Notebook)

For manuscripts utilizing custom algorithms or software that are central to the research but not yet described in published literature, software must be made available to editors and reviewers. We strongly encourage code deposition in a community repository (e.g. GitHub). See the Nature Research guidelines for submitting code & software for further information.

Data

Policy information about availability of data

All manuscripts must include a data availability statement. This statement should provide the following information, where applicable:

- Accession codes, unique identifiers, or web links for publicly available datasets
- A list of figures that have associated raw data
- A description of any restrictions on data availability

The authors declare that [the/all other] data supporting the findings of this study are available within the paper [and its supplementary information files].

Field-specific reporting

April 2020

Life sciences study design

All studies must disclose on these points even when the disclosure is negative.

Sample size	Ribbon length measurements: No sample-size calculation was performed. Sample size of N=113–168 (exact N for each time point reported in the figure legend of Figs. 3F,G, S29–S32, S35) measured ribbons from ~5–40 TEM micrographs where only complete ribbons (visible seed and ribbon end as well as no overlapping of ribbons) were measured manually with the line segment tool in ImageJ (v2.0.0-rc-69/1.52i). This sample size resulted in sufficient data points to calculate the mean length and standard deviation for different ribbon growth conditions.
Data exclusions	No data was excluded.
Replication	Experiments where agarose gel electrophoresis was used to quantify seeded or spurious ribbon growth were performed in triplicate experiments and in some cases as duplicate experiments. All attempts at replication were successful.
Randomization	Not relevant to this study. We characterized seeded and unseeded polymerization of DNA ribbons, for various monomer and salt concentrations and assembly temperatures.
Blinding	Not relevant to this study. The authors used protocols outlined in the methods section to conduct the experiments and reliably reproduce the results reported in the manuscript and supplementary information. For this, blinding was not necessary.

Reporting for specific materials, systems and methods

We require information from authors about some types of materials, experimental systems and methods used in many studies. Here, indicate whether each material, system or method listed is relevant to your study. If you are not sure if a list item applies to your research, read the appropriate section before selecting a response.

Materials & experimental systems

n/a	Involved in the study	
☒	☐	Antibodies
☒	☐	Eukaryotic cell lines
☒	☐	Palaeontology and archaeology
☒	☐	Animals and other organisms
☒	☐	Human research participants
☒	☐	Clinical data
☒	☐	Dual use research of concern

Methods

n/a	Involved in the study	
☒	☐	ChIP-seq
☒	☐	Flow cytometry
☒	☐	MRI-based neuroimaging
